# Timing and Extent of Inbreeding in African Goats

**DOI:** 10.3389/fgene.2019.00537

**Published:** 2019-06-04

**Authors:** Wilson Nandolo, Gábor Mészáros, Liveness Jessica Banda, Timothy N. Gondwe, Doreen Lamuno, Henry Aaron Mulindwa, Helen N. Nakimbugwe, Maria Wurzinger, Yuri T. Utsunomiya, M. Jennifer Woodward-Greene, Mei Liu, George Liu, Curtis P. Van Tassell, Ino Curik, Benjamin D. Rosen, Johann Sölkner

**Affiliations:** ^1^Division of Livestock Sciences, Department of Sustainable Agricultural Systems, University of Natural Resources and Life Sciences, Vienna, Vienna, Austria; ^2^Department of Animal Science, Faculty of Agriculture, Lilongwe University of Agriculture and Natural Resources, Lilongwe, Malawi; ^3^Animal Genomics and Improvement Laboratory, United States Department of Agriculture, Agriculture Research Service, Beltsville, MD, United States; ^4^National Livestock Resources Research Institute, Tororo, Uganda; ^5^Department of Agriculture, Kyambogo University, Kyambogo, Uganda; ^6^School of Agricultural and Veterinarian Sciences, Jaboticabal, São Paulo State University (UNESP), São Paulo, Brazil; ^7^Department of Animal Science, Faculty of Agriculture, University of Zagreb, Zagreb, Croatia

**Keywords:** inbreeding, age-based inbreeding, runs of homozygosity, local inbreeding, goats, Africa, copy number variation

## Abstract

Genetic characterization of African goats is one of the current priorities in the improvement of goats in the continent. This study contributes to the characterization effort by determining the levels and number of generations to common ancestors (“age”) associated with inbreeding in African goat breeds and identifies regions that contain copy number variation mistyped as being homozygous. Illumina 50k single nucleotide polymorphism genotype data for 608 goats from 31 breeds were used to compute the level and age of inbreeding at both local (marker) and global levels (F_G_) using a model-based approach based on a hidden Markov model. Runs of homozygosity (ROH) segments detected using the Viterbi algorithm led to ROH-based inbreeding coefficients for all ROH (F_ROH_) and for ROH longer than 2 Mb (F_ROH > 2Mb_). Some of the genomic regions identified as having ROH are likely to be hemizygous regions (copy number deletions) mistyped as homozygous regions. Although the proportion of these miscalled ROH is small and does not substantially affect estimates of levels of inbreeding for individual animals, the inbreeding metrics were adjusted by removing these regions from the ROH. All the inbreeding metrics varied widely across breeds, with overall means of 0.0408, 0.0370, and 0.0691 and medians of 0.0125, 0.0098, and 0.0366 for F_ROH_, F_ROH > 2Mb_, and F_G_, respectively. Several breeds (including Menabe and Sofia from Madagascar) had high proportions of recent inbreeding, while Small East African, Ethiopian, and most of the West African breeds (including West African Dwarf) had more ancient inbreeding.

## Introduction

Goats play a key role in the livelihoods of many communities in Africa. Besides being used for food and as income sources, they play crucial socio-cultural roles and are an integral part of the production system (Devendra, 2013). Goats are also key players in the resilience and adaptability of farmers to adverse conditions (Ribeiro et al., 2018). There is, therefore, a need to genetically improve goats for higher productivity. This change requires understanding the genetic architecture of goats, starting from the underlying genetic variation up to identification of causal mutations or quantitative trait loci. Much research has been carried out toward the characterization of African goat breeds, including the studies of Tucho and Tesfaye (2004) cataloging Ethiopian breeds; Traoré A. et al. (2012) looking at West African goats and Nguluma et al. (2018) understanding East African breeds. These and similar studies dealing with characterization of African goats were based on relatively few markers, usually microsatellites or allozymes, and these studies used limited samples. One of the pioneering works based on a large number of samples and a wide geographical representation using 50K single nucleotide polymorphism (SNP) markers for genomic characterization in addition to phenotypic description of up to 46 breeds from 12 African countries was by Huson et al. (2014). Knowledge gaps remain in this area, including little understanding of the levels of inbreeding and the dynamics of breeding decisions in various goat breeds, limited gene annotation, and a sparse map of CNV.

Traditionally, inbreeding has been estimated from pedigrees (Speed and Balding, 2015). This method is prone to errors caused by parentage recording mistakes as well as deviations from expected Mendelian sampling: animals born from the same parents have the same expected inbreeding level, while the exact level of inbreeding varies from animal to animal, even for full sibs. Methods of estimating inbreeding from high throughput genomic markers include estimation based on runs of homozygosity (ROH) (F_ROH_), as explained by McQuillan et al. (2008), excess homozygosity (Purcell et al., 2007), variance of additive genotypes (VanRaden, 2008), and a maximum-likelihood method that takes marker dependencies into account (Leutenegger et al., 2003). Currently, the most popular of these approaches is the use of ROH, which are continuous stretches of homozygous loci in the genome (Purfield et al., 2012). The proportion of an individual’s genome in ROH runs is an approximate measure of inbreeding (Curik et al., 2014), and it has been demonstrated that ROH inbreeding coefficients (F_ROH_) are closer to true inbreeding than pedigree inbreeding coefficient estimates (Keller et al., 2011). Wang (2016) demonstrated that if many markers are used (above 10,000), genomic inbreeding estimates can be more powerful in detecting variability and the effects of inbreeding.

The size of ROH are related to the age of inbreeding, with longer ROH runs most likely deriving from more recent common ancestors (Sölkner et al., 2010) and shorter ones deriving from older common ancestors. The distribution of ROH provides information about population history in terms of selection and other significant events in the past. However, some of these ROH could be copy number variant regions mistyped as homozygous regions (Nandolo et al., 2018). CNV are structural rearrangements of the genome involving from one thousand (kilo) base-pairs (kb) to several million (mega) base-pairs (Mb) (Redon et al., 2006). With Illumina SNP arrays, the GenTrain algorithm is used to determine the alleles at each position depending on the intensity of signals emitted by a probe at a specific marker compared to expected intensity [log R ratio (LRR)], and the proportion of hybridized sample that carries the B allele as designated by the hybridization assay (B-allele frequency, BAF), usually normalized to 0.0, 0.5, and 1.0 (Ritchie et al., 2011). The Illumina genotyping algorithm does not explicitly call deletions, and a hemizygous region is most likely to be typed as a homozygous one and consequently detected as such by ROH detection algorithms.

Recently, Druet and Gautier (2017) presented a model-based approach for estimating inbreeding at both genome-wide and local (SNP) levels. The approach implements a HMM that assumes that the genome is a mosaic of homozygous-by-descent (HBD) and non-HBD segments, with each segment having an HBD state probability. The HBD state probabilities can then be used to compute global inbreeding (F_G_) for each individual and for the population. The approach has been demonstrated to estimate inbreeding coefficients and consequently inbreeding levels with the distance to their reference populations accurately, even at low SNP density (Solé et al., 2017).

The main goals of this study were to provide fine-scale estimates of inbreeding levels in African goats and to correctly identify genomic regions with increased autozygosity which often arise because of recent selection. This is the first study where, both, recent improvements in the estimation of inbreeding levels (Druet and Gautier, 2017; Solé et al., 2017) and CNV hemizygote correction in estimating local autozygosity (Nandolo et al., 2018) were implemented. Our results are expected to contribute to the conservation and breeding management of African goat populations.

## Materials and Methods

**FIGURE 1:**
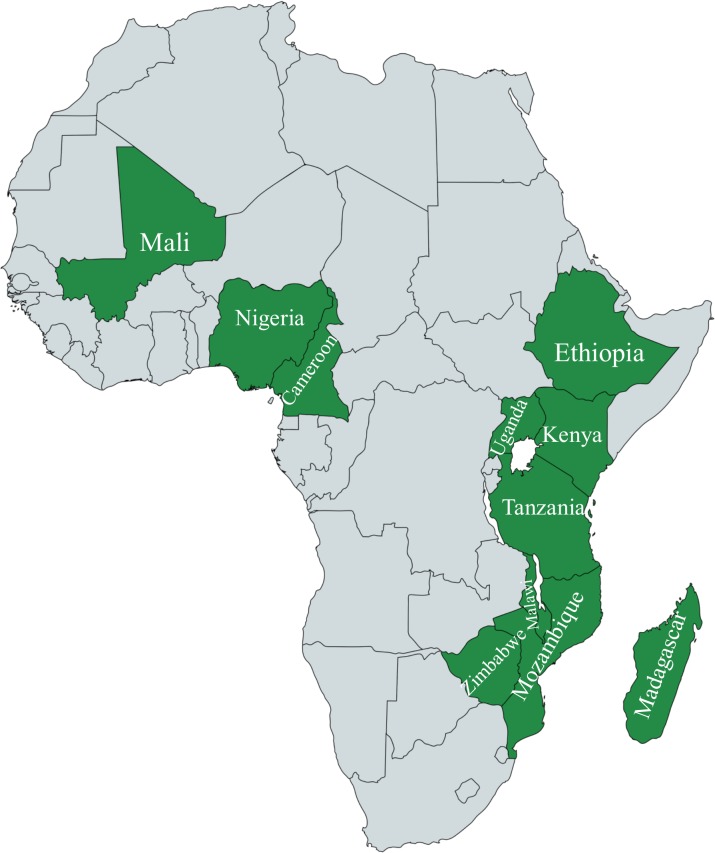
Countries from which data were collected on the African Continent. Created with MapChart (www.mapchart.net).

The data used in this study were a subset of the AdaptMap dataset (Stella et al., 2018) comprising 608 goats belonging to 31 breeds from 11 Sub-Saharan African countries as shown in Figure 1 (Cameroon, Ethiopia, Kenya, Madagascar, Malawi, Mali, Mozambique, Nigeria, Tanzania, Uganda, and Zimbabwe).

The animals were genotyped using the Illumina Goat 50k BeadChip (Tosser-Klopp et al., 2014), containing 52,295 SNP loci. Preliminary quality control (QC) included filtering for individual call rate (≤4%) and SNP call rate (≤2%), leading to a total number of 48,827 markers, with all the animals passing QC. Additionally, we filtered out markers that significantly deviated from the Hardy-Weinberg equilibrium (*p* < 0.001). Supplementary Table 1 shows the breeds and number of animals used in the study and the QC statistics. The animals were also used for detection of CNV as reported by Liu et al. (2018).

### Inbreeding Analysis

Computation of inbreeding was done using ZooRoH software (Druet and Gautier, 2017). The software implements a HMM which uses marker allele frequencies, genetic distances, genotyping error rates, and the sequences of observed genotypes to determine the probability of each locus being HBD. The initial step in the analysis is the splitting of the genome into *K* HBD classes and one non-HBD class. The basic model is where a genome segment is either HBD or non-HBD (*K* = 1). The estimated number of recombination events to a common ancestor is used to estimate the rate of ancestry change (*R*), which defines the distribution of segments in each of the *K* classes. If no recombination occurs in both paths to a common ancestor, the rate for that diploid segment is *R* = *2G* where *G* is the number of generations to the common ancestor. *R* depends on time to common ancestors (Leutenegger et al., 2003; Vieira et al., 2016; Druet and Gautier, 2017), and is directly related to the age (in generations) of the common ancestors for each class (Solé et al., 2017). The smaller the value of *R*, the more recent the segments. The rates can be estimated from the model, but an alternative approach is to use predetermined rates and estimate the mixing proportions (the proportions of segments belonging to each of the *K* predetermined classes).

In this study, we used 11 HBD classes with predetermined rates (equivalent to number of generations) of *G* = 2, 4, 8, …, 2048 and 2048 for the non-HBD class. We chose 12 classes to efficiently capture both genome-wide and local (SNP-level) inbreeding while at the same time obtaining as much information about the inbreeding history of the animals as possible. Other parameters used in the model in this study included genotype error rate and minimum minor allele frequency (MAF), which were set to 0.001 and 0.01, respectively. A 12-by-12 identity matrix was used as the transition matrix defining the allowable HBD state changes, implying that each genomic segment could change from one class to any other class.

The major outputs from the model were HBD state probability values for each marker. The HBD state probabilities were averaged across individuals to get local inbreeding for each marker. Similarly, HBD state probabilities for segments belonging to class K_i_ were averaged across the whole genome to get the inbreeding estimate for that class. Averaging HBD probabilities of all loci across the genome led to global (genome-wide) inbreeding (F_G_). A Viterbi algorithm (Eddy, 2004) implemented in the ZooRoH software was used to determine which markers were in contiguous segments large enough to be considered ROH segments based on the local HBD state probabilities, with a cut-off inbreeding level of 0.99. The ROH were used to compute a ROH-based coefficient of inbreeding for all ROH (F_ROH_) and for ROH longer than 2 Mb (F_*ROH* > 2Mb_) for comparability with other studies. Proportions of each HBD class for local and genome-wide (global) inbreeding (F_G_) were plotted within and across breeds using R (R Development Core Team, 2016).

### CNV Analysis

Copy number variation analysis results for the animals were obtained from a companion study aimed at mapping CNV in the goat genome (Liu et al., 2018). The CNV were detected using a HMM implemented in PennCNV software (Wang et al., 2007) with LRR standard deviation, absolute (genomic) wave factor, B-allele frequency drift, and maximum number of CNV per sample equal to 0.3, 0.05, 0.01, and 100, respectively.

### Intersections Between ROH and CNV With Copy Loss

Intersections between ROH and CNV with copy losses (abbreviated as ROH-CL in this study) in each animal were computed using BEDTools (Quinlan and Hall, 2010). BAF and LRR values were plotted for the regions with ROH and ROH-CL. Regions with mean LRR less than −0.3 were considered as copy losses. The lengths of these regions were subtracted from F_ROH,_ F_ROH > 2*Mb*,_ and F_G_ to adjust the inbreeding values for the existence of the CNV that had been mistakenly called as ROH.

### Comparison of Adjusted F_ROH_, F_ROH > 2Mb_, and F_G_

Differences in levels of inbreeding based on the three metrics F_ROH_, F_ROH > 2Mb_, and F_G_ were tested using a general linear model with breed as the other source of variation. Pairwise comparisons between the inbreeding metrics and between the breeds were adjusted using Bonferroni correction.

## Results

### Distribution of HBD Segments

**FIGURE 2:**
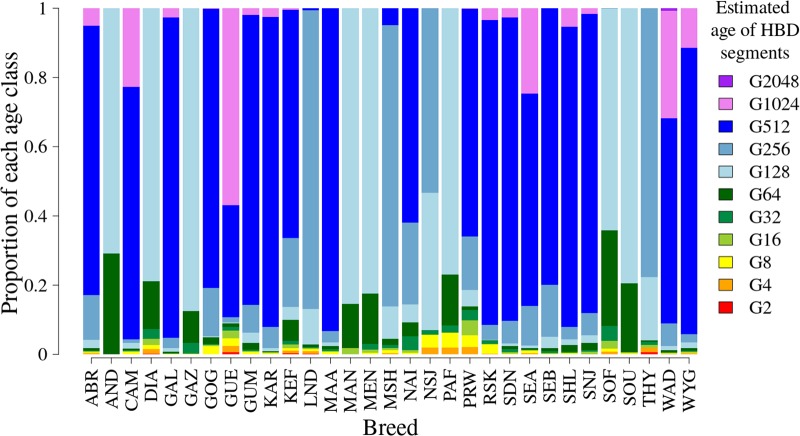
The distribution of the mean proportion of number of segments for the 11 HBD classes. Each HBD class corresponds to the generation from which the segments originated, starting from the second generation (G2) to generation number 2048 (G2048). Breed codes: ABR, Abergelle (Ethiopia); AND, Androy (Madagascar); CAM, Cameroon (Cameroon); DIA, Diana (Madagascar); GAL, Galla (Kenya); GAZ, Gaza (Mozambique); GOG, Gogo (Tanzania); GUE, Guéra (Mali); GUM, Gumez (Ethiopia); KAR, Karamoja (Uganda); KEF, Keffa (Ethiopia); LND, Landim (Mozambique); MAA, Maasai (Tanzania); MAN, Manica (Mozambique); MEN, Menabe (Madagascar); MSH, Mashona (Zimbabwe); NAI, Naine (Mali); NSJ, Nsanje (Malawi); PAF, Pafuri (Mozambique); PRW, Pare White (Tanzania); RSK, Red Sokoto (Nigeria); SDN, Soudanaise (Mali); SEA, Small East African (Kenya and Mozambique); SEB, Sebei (Uganda); SHL, Sahel (Nigeria); SNJ, Sonjo (Tanzania); SOF, Sofia (Madagascar); SOU, SudOuest (Madagascar); THY, Thyolo (Malawi); WAD, West African Dwarf (Cameroon and Nigeria); WYG, Woyito Guji (Ethiopia).

Most of the genome segments identified by ZooRoH (99.1%) were non-HBD. The distribution of the HBD segments across the 11 HBD-classes for each breed is shown in Figure 2. For most of the breeds, a large proportion of the segments originated from approximately 500 generations ago. Breeds with more recent segments included the Madagascar breeds (Androy, Diana, Menabe, Sofia, and SudOuest) and three of the Mozambique breeds (Gaza, Manica, and Pafuri). Several breeds had segments originating from approximately 1000 generations ago, including CAM, GUE, Small East African (Kenya, Uganda, and Mozambique), and WAD.

### Timing of Inbreeding

#### Local Inbreeding

The distribution of local inbreeding for each breed is given in Supplementary Figure 1. Chromosome-wise, there was a lot of variation in the distribution of the proportions of local inbreeding attributable to each of the 11 HBD classes. Some of the breeds had large proportions of ancient inbreeding with a few genomic regions with more recent inbreeding. Typical examples in this category were the CAM and GAL goats, with proportions of segments aged 512 to 2048 generations amounting up to 96 and 89%, respectively. Breeds such as Androy and Sofia (both from Madagascar) had significant proportions of more recent inbreeding, with proportions of recent segments (up to 64 generations ago) of between 15 and 40%.

The Madagascar breeds had HBD segments mostly originating from 2 to 128 generations ago with variable proportions of genomic regions having HBD segments attributable to the last 2 to 4 generations.

An example of breeds with intermediate age of inbreeding was THY, with a mixture of both very recent (2–32 generations) and moderately distant (approximately 256) generations ago. In the Thyolo breed, chromosomes 8 and 29 predominantly had segments from approximately 256 generations ago. Most of the other breeds had HBD segments originating from moderately distant (256) to very distant generations ago with a few genomic regions having HBD segments attributable to recent generations. The Guéra breed (Mali) stood out in having significant proportions of both ancient and recent inbreeding.

#### Global Inbreeding

Detailed plots of F_G_ within each breed are given in Supplementary Figure 2. Menabe and Sofia showed recent inbreeding going back 8 to 64 generations ago. Malawian, LND, Mashona and Matebele breeds (Zimbabwe) had intermediate age of inbreeding (128 to 256 generations ago). Small East African, Abergelle, Gumez, Keffa, and WYG and most of the West African breeds (including West African Dwarf) had large proportions of their inbreeding resulting from common ancestry back to 128 to 2048 generations ago, which is the approximate time that the first goat domestication event took place approximately 10,000 years ago (Zeder and Hesse, 2000).

### ROH-CNV Loss Intersections (ROH-CL)

A total of 3,969 CNV were detected in the 608 animals, corresponding to an average of 6.53 CNV for each animal. The distribution of the copy numbers 0, 1, 3, and 4 was 59, 2958, 942, and 10, where copy numbers 0 and 1 represent deletions and 3 and 4 are duplications. Out of the 608 goats, 484 goats had ROH (using the definition of ROH in this study) and CNV with copy losses, and 139 had ROH-CL on 205 unique regions across breeds (Supplementary Table 2). Only 50 of the 205 regions were shared by two or more animals. ROH-CL covered 57.4 Mb across all the 608 animals, including shared regions. This coverage represented about 2.3% of the goat genome. The size of the regions varied widely between individuals, with mean, median, minimum, and maximum values of 123, 0, 0, and 13688 kb, respectively. On average, ROH-CL accounted for 0.129 and 0.049% of unadjusted F_ROH_ and F_G_, respectively.

**FIGURE 3:**
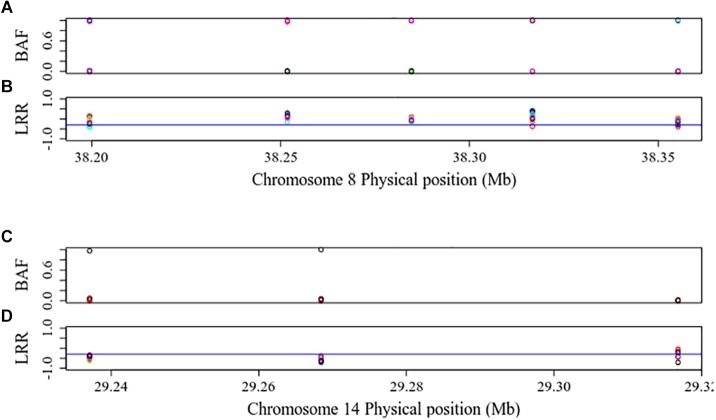
BAF and LRR in a ROH existing in 28 individuals from 15 breeds (Abergelle, Diana, Guéra, Gumez, Keffa, Landim, Menabe, Naine, Nsanje, Red Sokoto, Small east African, Sofia, SudOuest, Thyolo, and West African Dwarf) on CHI8 (38,199,425 to 38,355,253 bp) **(A,B)** and in a ROH-CL in 10 individuals from 6 breeds (Cameroon, Diana, Karamoja, Menabe, Small east African, and West African Dwarf) on CHI14 (29,237,079 to 29,316,817 bp) **(C,D)**. The blue lines are LRR cut-off points of –0.3, below which the signal intensity may mean that there is only one allele, implying copy loss.

**FIGURE 4:**
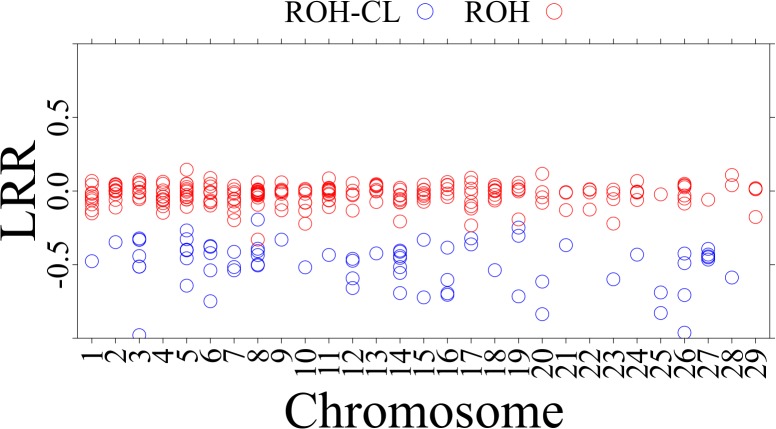
Distribution of mean LRR values in the sampled ROH and in the ROH-CL. The red dots are for ROH while the blue dots are for ROH-CL intersections.

Figure 3 shows the distribution of BAF and LRR in an ROH existing in 28 individuals from 15 breeds (Abergelle, Diana, Guéra, Gumez, Keffa, Landim, Menabe, Naine, Nsanje, Red Sokoto, Small East African, Sofia, SudOuest, Thyolo, and West African Dwarf) on CHI8 (38,199,425 to 38,355,253 bp) and a ROH-CL in 10 individuals from 6 breeds (Cameroon, Diana, Karamoja, Menabe, Small East African, and West African Dwarf) on CHI14 (29,237,079 to 29,316,817 bp). The BAF values are consistent with the regions being homozygous (Alkan et al., 2011), but the LRR values for the ROH-CL are mostly below −0.3, consistent with copy losses. Figure 4 shows the distribution of mean LRR values in randomly sampled ROH and in all the ROH-CL. The figure suggests that the ROH-CL are indeed CNV regions with copy loss miscalled as ROH, most likely because of hemizygosity.

### Distribution of ROH Lengths

**FIGURE 5:**
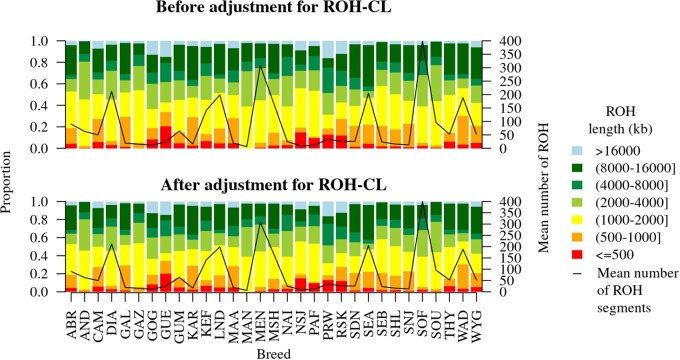
Distribution of ROH sizes before and after adjusting for ROH-CNV copy loss intersections. The two plots differ by from 0 to 0.0168 depending on the breed.

The distribution of the sizes of ROH as identified by the Viterbi algorithm based on the HBD state probabilities in each breed before and after adjusting for ROH-CNV intersections is shown in Figure 5. Proportions of different ROH size categories increased or decreased from 0 up to 0.0168 after adjusting for the ROH-CNV intersections. Overall, breeds that stood out were GUE and WAD, both of which had more than 30% ROH shorter than 1 Mb compared to the other breeds. Ten breeds had relatively higher proportions of ROH longer than 8 Mb (from just about 30% in the Mashona up to 42% in the Small East African breed).

### Inbreeding Adjusted for ROH-Copy Loss Intersections ROH-CL

**FIGURE 6:**
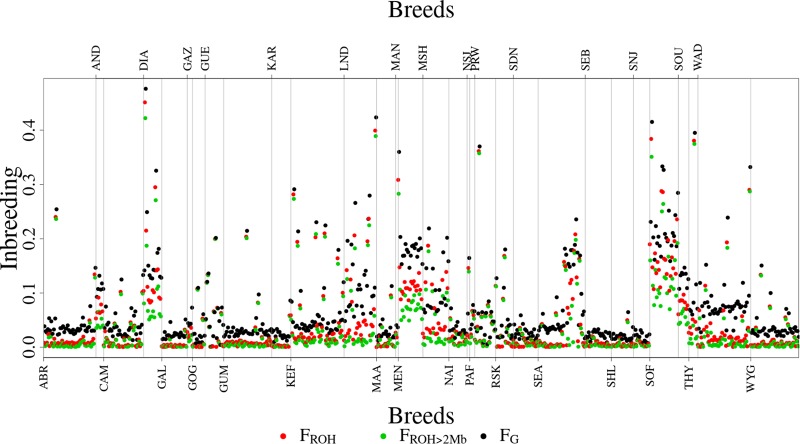
Distribution of the inbreeding metrics (F_G_, F_ROH_, and F_ROH >2Mb_) for all the 608 animals by breed.

**FIGURE 7:**
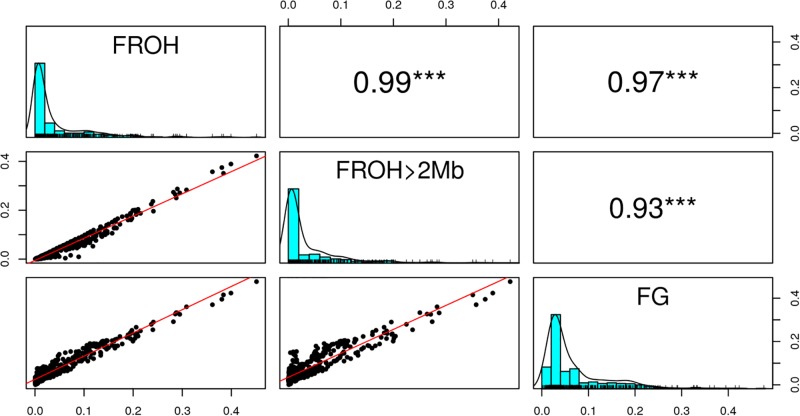
Overall correlation between the inbreeding metrics (F_ROH_, F_ROH >2Mb_, and F_G_. ^∗∗∗^Significant at *p* < 0.001).

Figure 6 shows the distribution of F_ROH_, F_ROH > 2Mb_ and F_G_ values for each of the 31 breeds. Descriptive statistics of the metrics for the breeds are given in Supplementary Table 3. As expected, inbreeding values increased from F_ROH > 2Mb_ through F_ROH_ to F_G_. The metrics averaged 0.0408 (0.0708), 0.0367 (0.0642), and 0.0691 (0.0618) across breeds, as given in Table 1. The overall correlations between F_ROH_ and the other metrics (F_ROH > 2Mb_ and F_G_) were 0.99 and 0.97, respectively, while the correlation between F_ROH > 2Mb_ and F_G_ was 0.93. In the correlation between F_ROH > 2Mb_ and F_G,_ small values deviated significantly from the linear plot (Figure 7).

**Table 1:** Overall descriptive statistics of F_ROH_, F_ROH > 2Mb_, and F_G_ metrics (*N* = 60).

Metric	Minimum	Q1	Median	Mean	Standard deviation	Q3	Maximum
F_G_	0.0012	0.0249	0.0366	0.0691	0.0708	0.0818	0.4762
F_ROH_	0.0002	0.0045	0.0125	0.0408	0.0642	0.0446	0.4512
F_ROH > 2Mb_	0.0006	0.0032	0.0097	0.0370	0.0618	0.0486	0.4221

The top three most inbred breeds were from Madagascar: Sofia (F_ROH_ = 17.68%, F_ROH > 2Mb_ = 14.17%, and F_G_ = 22.61%), Diana (F_ROH_ = 15.12%, F_ROH > 2Mb_ = 11.79%, and F_G_ = 18.77%) and Menabe (F_ROH_ = 12.09%, F_ROH > 2Mb_ = 8.56%, and F_G_ = 18.64%). Additional breeds that were highly inbred were NSJ, THY, LND, and SOU. The least inbred goats were the Galla from Kenya (F_ROH_ = 0.46%, F_ROH > 2Mb_ = 0.43%, and F_G_ = 2.39%). Additional breeds that had low inbreeding were SNJ, SEB, and SHL.

**FIGURE 8:**
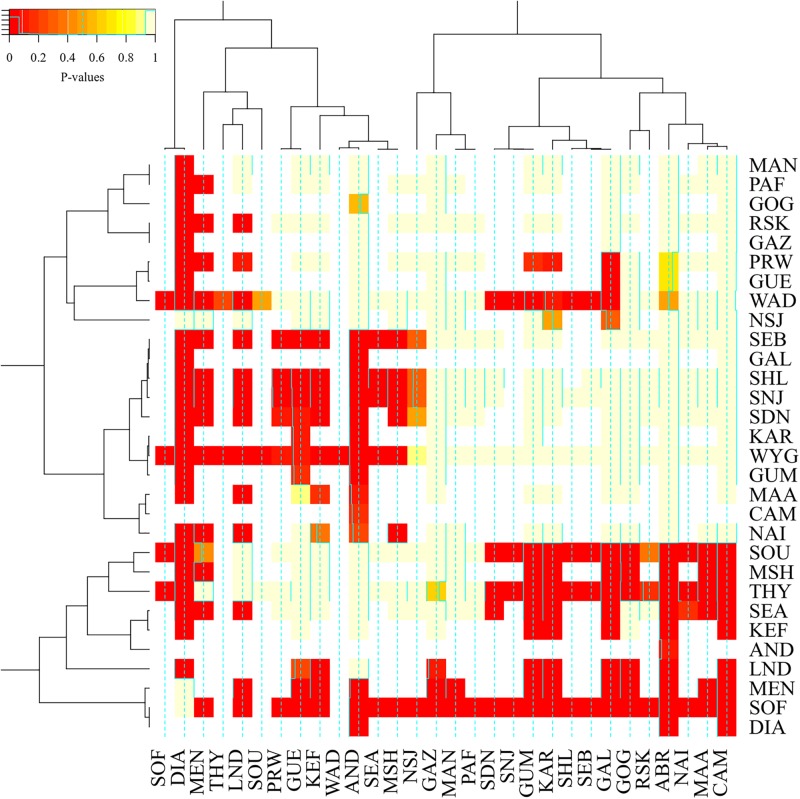
A heat-map plot of the *p*-values for the pair-wise comparison of inbreeding values between breeds. The darker the red color, the more different the breeds are in terms of inbreeding.

We also ran a general linear model to test the differences in levels of inbreeding based on the three metrics (F_ROH_, F_ROH > 2Mb_, and F_G_) and breeds. The differences between the breeds in inbreeding level were significant (*p*-value = 2.2e-16). Generally, breeds from Madagascar (Androy, Diana, Menabe, Sofia, and SudOuest) were highly inbred and grouped together with MSH, THY, SEA, and LND breeds, as shown in the heat-map of *p*-values of pairwise breed differences in inbreeding coefficients in Figure 8. On the other hand, West African breeds were generally less inbred than the other breeds. There were also significant differences between F_G_ and the other two metrics, F_ROH_ (*P* = 3.8e-13) and F_ROH > 2Mb_ (*P* = 1.6e-15) while F_ROH_ and F_ROH > 2Mb_ were not different (*P* = 0.98).

## Discussion

This study presents a comprehensive report on the level of inbreeding in goat breeds across the African continent. Information about the level of inbreeding of a population has practical applications in management and improvement of the population. The primary need for knowing the inbreeding level of a population is that inbreeding may lead to the depression of traits by as much as 0.137% for every 1% of inbreeding (Leroy, 2014). Such knowledge would then inform on appropriate animal breeding and genetic resources management and conservation.

There are many regions on the goat genome where ROH intersect with copy losses. The distribution of BAF and LRR values in those regions are highly consistent with the possibility that they are hemizygous, which would suggest that the copy losses in those regions are being miscalled as ROH. At a mean of 123 kb per individual, these miscalled regions are too small to affect inbreeding coefficients, however, they could impact analysis of specific regions of interest in the genome and should therefore be taken into consideration for more targeted analyses.

The results indicate that there is a wide variation in the distribution of the number of genomic segments by age, which gives insight into the history of the breeds. Breeds with large proportions of segments in older generations (Cameroon, Guéra, Small East African, West African Dwarf, Woyito Guji, and Abergelle) have their common ancestors in the distant past. The results show that the distributions of the sizes of the ROH in individuals were highly variable across breeds.

There is apparent relationship between location of the goats and the level of inbreeding. Goats from Madagascar and Southeastern Africa appear to be more highly inbred than goats from Western Africa. This may be a reflection on differences in goat management and or differences in breed histories. Goats in Madagascar are mostly raised in the Southwestern region (Feldt et al., 2016). It is possible that the goats are secluded in the region with the highlands to northeast acting as barriers, thereby leading to higher levels of inbreeding. Bertolini et al. (2018) and Cardoso et al. (2018) also noted high levels of inbreeding in the Madagascar goats.

F_G_ values are higher than F_ROH_ and F_ROH > 2Mb_ values, especially significant for breeds with low inbreeding in relative terms. This could be because with medium density SNP chip data, the Viterbi algorithm has difficulties detecting very short ROH, although the probability of the markers in those regions belonging to any of the HBD classes can be estimated, leading to F_G_ values higher than F_ROH_ values. The correlations among the metrics were high and positive.

Global inbreeding values were only slightly higher than the ROH-based inbreeding values in the Guéra from Mali and Pare White from Tanzania. Guéra goats in Mali were imported from Mauritania (Traoré D. et al., 2012), where they were originally derived from Spanish goats (Missohou et al., 2006). Recent inbreeding in the imported populations may have led to large ROH segments, leading to the similarity in the values of the three metrics. Pare White goats may also have long ROH segments due to intentional inbreeding as farmers try to preserve the white coat color of the breed (Nguluma et al., 2016).

It is also worthwhile to note that the standard deviations of the inbreeding metrics (especially F_G_) within breeds were high. On the other hand, the overall mean values of the metrics across breeds (0.0408, 0.0370, and 0.0691 for F_ROH_, F_ROH > 2Mb_, and F_G_, respectively) compare reasonably well with those reported by other researchers for the species. Brito et al. (2017) found that F_ROH_ averages around 0.038 in some common breeds (Alpine, Boer, LaMancha, Nubian, Saanen and Toggenburg, and Cashmere and Rangeland). The F_ROH_ values found in this study for the Ethiopian goats are similar to F_ROH > 1*Mb*_ values found by Nandolo et al. (2017) using a clustering algorithm (Zhang et al., 2013) in Abergelle (0.02), Gumez (0.02), Keffa (0.05), and Woyito Guji (0.02). The F_ROH > 2Mb_ results from this study are lower than the F_ROH > 2*Mb*_ results reported by Onzima et al. (2018) for Karamoja, Sebei, and Small East African (Kenya, Uganda, and Mozambique) goat breeds.

The Viterbi algorithm can reliably detect ROH shorter than 1 Mb from 52k SNP, which is an improvement on algorithms that use the clustering approach to detect ROH. Although Ferenčaković et al. (2013) has shown that ROH < 4 Mb are biased at this SNP density, the FROH results from this study can be relied on because ZooRoH HBD segments are centered on markers with total HBD probabilities higher than 0.999, with each segment encompassing all surrounding markers with a total HBD probability higher than 0.99 and stopping with the first marker with a probability below 0.99. This could also explain why F_*ROH* > 2Mb_ results from this study are lower than values from those found by Onzima et al. (2018). Additionally, the ZooRoH model incorporates population marker frequencies when computing marker HBD state probabilities.

The F_ROH_ results are also lower than those reported by Cardoso et al. (2018) for the Madagascar and Zimbabwe breeds using the same data but a different ROH-calling algorithm. The difference could be due to the reasons explained above, but also because one heterozygous SNP was allowed per ROH in the other study, possibly leading to longer ROH. The high proportion of ROH shorter than 1 Mb in breeds such as Guéra and Nsanje is likely the reason that the F_G_ values for these breeds are among the lowest, despite the age of inbreeding being low to moderate. Colli et al. (2018) observed that there has been significant introgression of other breeds into African goat breeds, leading to lower levels of inbreeding. Additionally, Colli et al. (2018) found that the African breeds do not seem to have gone through the same significant reduction in effective population sizes as breeds in other regions during the last 50 generations, possibly leading to shorter HBD segments.

Short BD segments may also point to a lot of outcrossing in the African goat breeds. This is possible considering the extensive management systems of goats in the region, where goats are on free range or are herded with other flocks for some part of the year, although some researchers have argued that such extensive systems may lead to inbreeding (Tefera et al., 2004; Gwaze et al., 2009). Manunza et al. (2016) suggested that the lower inbreeding levels in African goats could be due to the openness of the breeding systems in most of Africa due to the loose definition of livestock breeds in the region. The high proportion of ROH longer than 8 Mb in the Small East African breed may be an indication of less outcrossing.

## Conclusion

African goats show a wide range of inbreeding properties, both in the level and estimated generations to common ancestors associated with inbreeding. Contrary to popular expectation, many of the African goat breeds have low levels of inbreeding, and some of them have large proportions of HBD genome segments that date back to about the beginning of domestication of goats, implying that most of these breeds have had minimal contact with distantly related breeds.

## Data Availability

The data is available on Dryad, https://doi.org/10.5061/dryad.v8g21pt.

## Author Contributions

BR and CVT prepared the preliminary data. WN, GM, BR, CVT, and JS analyzed the data. WN produced the draft manuscript. LB, TG, DL, HM, HN, MW, BR, MW-G, and CVT reviewed and revised the manuscript. All authors read and approved the final draft.

## Conflict of Interest Statement

The authors declare that the research was conducted in the absence of any commercial or financial relationships that could be construed as a potential conflict of interest. The reviewer CF declared a past co-authorship with one of the authors, JS, to the handling Editor.

## Abbreviations

ABR: Abergelle (Ethiopia)
AND: Androy (Madagascar)
CAM: Cameroon (Cameroon)
CHI: Capra hircus
CNV: copy number variation
DIA: Diana (Madagascar)
GAL: Galla (Kenya)
GAZ: Gaza (Mozambique)
GOG: Gogo (Tanzania)
GUE: Guéra (Mali)
GUM: Gumez (Ethiopia)
HMM: hidden Markov model
KAR: Karamoja (Uganda)
KEF: Keffa (Ethiopia)
LND: Landim (Mozambique)
MAA: Maasai (Tanzania)
MAN: Manica (Mozambique)
MEN: Menabe (Madagascar)
MSH: Mashona (Zimbabwe)
NAI: Naine (Mali)
NSJ: Nsanje (Malawi)
PAF: Pafuri (Mozambique)
PRW: Pare White (Tanzania)
RSK: Red Sokoto (Nigeria)
SDN: Soudanaise (Mali)
SEA: Small East African (Kenya and Mozambique)
SEB: Sebei (Uganda)
SHL: Sahel (Nigeria)
SNJ: Sonjo (Tanzania)
SOF: Sofia (Madagascar)
SOU: SudOuest (Madagascar)
THY: Thyolo (Malawi)
WAD: West African Dwarf (Cameroon and Nigeria)
WYG: Woyto Guji (Ethiopia)
