# Supplementary material for: Timing and Extent of Inbreeding in African Goats
Source: Front Genet. 2019 Jun 4;10:537. doi: 10.3389/fgene.2019.00537 (PMC6558083; doi:10.3389/fgene.2019.00537)
Supplement: Supplementary file 1 [file Data_Sheet_1.pdf]

Timing and extent of inbreeding in African goats

Wilson Nandolo, Gábor Mészáros, Liveness Jessica Banda, Timothy N. Gondwe, Doreen Lamuno, Henry Aaron Mulindwa, Helen N. Nakimbugwe, Maria Wurzinger, Yuri T. Utsunomiya, Benjamin D. Rosen*, M. Jennifer Woodward-Greene, Mei Liu, George Liu, Curt P. van Tassell, Ino Curik, Johann Sölkner

*** Correspondence:** Dr. Benjamin D. Rosen: ben.rosen@ars.usda.gov

1 Supplementary Tables

Supplementary Table 1: Breeds, number of animals and data quality control statistics

Breed code	Breed Name	Countries	Number of Animals	SNPs after preliminary QC	Autosomal SNPs	SNPs filtered due to HWE correction	SNPs which passed QC
ABR	Abergelle	Ethiopia	49	48827	46654	33	46621
AND	Androy	Madagascar	6	48827	46654	0	46654
CAM	Cameroon	Cameroon	37	48827	46654	21	46633
DIA	Diana	Madagascar	14	48827	46654	15	46639
GAL	Galla	Kenya	23	48827	46654	7	46647
GAZ	Gaza	Mozambique	4	48827	46654	0	46654
GOG	Gogo	Tanzania	12	48827	46654	9	46645
GUE	Guera	Mali	16	48827	46654	3	46651
GUM	Gumez	Ethiopia	39	48827	46654	25	46629
KAR	Karamoja	Uganda	19	48827	46654	14	46640
KEF	Keffa	Ethiopia	44	48827	46654	38	46616
LND	Landim	Mozambique	29	48827	46654	47	46607
MAA	Maasai	Tanzania	18	48827	46654	15	46639
MAN	Manica	Mozambique	3	48827	46654	0	46654
MEN	Menabe	Madagascar	19	48827	46654	11	46643
MSH	Mashona	Zimbabwe	22	48827	46654	17	46637
NAI	Naine	Mali	14	48827	46654	10	46644
NSJ	Nsanje	Malawi	6	48827	46654	0	46654
PAF	Pafuri	Mozambique	4	48827	46654	0	46654
PRW	Pare White	Tanzania	19	48827	46654	14	46640

Supplementary Material

**Breed code**	**Breed Name**	**Countries**	**Number of Animals**	**SNPs after preliminary QC**	**Autosomal SNPs**	**SNPs filtered due to HWE correct ion**	**SNPs which passed QC**
RSK	Red Sokoto	Nigeria	19	48827	46654	19	46635
SDN	Soudanaise	Mali	22	48827	46654	14	46640
SEA	Small East African	Kenya, Mozambique	48	48827	46654	127	46527
SEB	Sebei	Uganda	21	48827	46654	7	46647
SHL	Sahel	Nigeria	19	48827	46654	17	46637
SNJ	Sonjo	Tanzania	20	48827	46654	16	46638
SOF	Sofia	Madagascar	22	48827	46654	28	46626
SOU	SudOuest	Madagascar	8	48827	46654	0	46654
THY	Thyolo	Malawi	9	48827	46654	0	46654
WAD	West African Dwarf	Cameroon, Nigeria	50	48827	46654	95	46559
WYG	Woyito Guji	Ethiopia	39	48827	46654	19	46635

Supplementary Table 2: Intersections between CNV with copy loss and ROH

Chromosome	Start	End	Length	Number of animals in intersection
1	132391673	132501677	110004	2
1	10841457	10935098	93641	1
1	11709355	11773962	64607	1
1	12631904	12706152	74248	1
1	15133823	15243578	109755	1
1	89497319	89623592	126273	1
1	109966002	110442812	476810	1
1	112348625	112439038	90413	1
1	140468003	140708692	240689	1
1	153778090	153934330	156240	1
1	156977603	157299677	322074	1
2	1758747	2042704	283957	2
2	32998411	33072221	73810	1
2	52750539	53135362	384823	1
2	53678790	53978017	299227	1
2	54249365	54319168	69803	1
2	55105878	55191169	85291	1
2	69487940	69678079	190139	1
2	86457515	86765696	308181	1
2	92655933	92753615	97682	1
2	95107360	95188662	81302	1
2	99294741	99399810	105069	1
2	106396405	106796502	400097	1
2	124241334	124821874	580540	1
2	125789320	125859734	70414	1
2	133557185	133676327	119142	1
3	80113767	80835877	722110	8
3	95491550	95583652	92102	4
3	56318669	56519568	200899	3
3	2273298	2431029	157731	1
3	3026943	3161053	134110	1
3	5125312	5705846	580534	1
3	75223843	75401597	177754	1
3	82142013	82516080	374067	1
3	83013095	83511297	498202	1

Supplementary Material

Chromosome	Start	End	Length	Number of animals in intersection
4	211217	469254	258037	1
4	4944393	5122279	177886	1
4	8320649	8390076	69427	1
4	36633924	36715697	81773	1
4	41432593	41543705	111112	1
4	55713921	55819278	105357	1
4	57538222	57607573	69351	1
4	90483172	90572038	88866	1
4	92205915	92349001	143086	1
4	94198160	94721263	523103	1
4	96191967	96327511	135544	1
4	100720109	100851020	130911	1
4	109049997	109136676	86679	1
5	75604240	77148831	1544591	7
5	11659559	13201538	1541979	2
5	36576428	36651321	74893	2
5	65573063	65798311	225248	2
5	13500683	13889737	389054	1
5	16542410	16665848	123438	1
5	57922192	58333613	411421	1
5	118593642	118885547	291905	1
6	116345392	117571225	1225833	3
6	75555962	75747770	191808	2
6	85946704	85994156	47452	2
6	114512108	114896769	384661	2
6	28261769	28441193	179424	1
6	37898813	38091505	192692	1
6	50860004	51009257	149253	1
6	80047974	80176656	128682	1
6	104271586	104358436	86850	1
7	107678302	108372414	694112	6
7	106583259	106771982	188723	2
7	1227401	3004872	1777471	1
7	3581367	4029290	447923	1
7	15423801	15497158	73357	1
7	68013445	68478315	464870	1
7	68702875	68781064	78189	1

Supplementary Material

Chromosome	Start	End	Length	Number of animals in intersection
7	72834821	73050578	215757	1
7	88810208	89043687	233479	1
7	90119745	90322669	202924	1
8	636594	1109062	472468	3
8	111489684	112561993	1072309	3
8	2413253	2509109	95856	2
8	19101859	19296598	194739	1
8	30000978	30054120	53142	1
8	80244299	80395844	151545	1
8	109940432	110083255	142823	1
8	111379886	111416864	36978	1
9	15159499	15334673	175174	2
9	10228158	10311903	83745	1
9	16048162	16151766	103604	1
9	20737498	21435382	697884	1
9	33029974	33216654	186680	1
10	78819051	78893302	74251	2
10	2289488	2373609	84121	1
10	14767788	14840022	72234	1
10	14869937	14962981	93044	1
10	19055481	19152341	96860	1
10	47453159	47701890	248731	1
10	51848580	51927535	78955	1
10	57679258	57949639	270381	1
10	64695254	64811188	115934	1
10	80399500	80457410	57910	1
11	33916409	34109741	193332	2
11	36974191	37095363	121172	1
11	39225424	39502831	277407	1
11	82348757	82597945	249188	1
11	103005508	103216934	211426	1
11	105917369	106108732	191363	1
12	42976509	43300094	323585	3
12	57668107	57804796	136689	2
12	78555625	79016897	461272	2
12	1399006	1431541	32535	1
12	12830122	12972454	142332	1

Supplementary Material

Chromosome	Start	End	Length	Number of animals in intersection
12	41005376	41659094	653718	1
12	45680519	45896052	215533	1
12	48513908	48715730	201822	1
12	79645657	79850276	204619	1
12	81331464	81481916	150452	1
13	82859699	83017136	157437	2
13	45521775	45681815	160040	1
13	55874198	56703165	828967	1
14	29164525	29386286	221761	11
14	32404327	32573226	168899	2
14	78479966	78947459	467493	2
14	87999719	88237645	237926	2
14	8630351	8780447	150096	1
14	40410105	41216216	806111	1
14	72523575	72603732	80157	1
14	81146492	81409605	263113	1
15	71378428	71780467	402039	4
15	26634192	26705819	71627	1
15	46411275	46520625	109350	1
15	73340784	73927930	587146	1
15	77432122	77679672	247550	1
15	79128030	79511043	383013	1
16	34151967	34231258	79291	3
16	74622584	74705369	82785	3
16	77668969	79353771	1684802	3
16	28987486	29263185	275699	1
16	64812585	65050228	237643	1
17	38453012	38660495	207483	2
17	63012634	63391313	378679	2
17	26179439	26325287	145848	1
17	30853999	30953291	99292	1
17	32264686	32864269	599583	1
17	51875297	52228428	353131	1
17	66645508	66826746	181238	1
17	70777430	71114809	337379	1
18	57911618	57982792	71174	2
18	2529845	2712383	182538	1

Supplementary Material

Chromosome	Start	End	Length	Number of animals in intersection
18	3691395	3853184	161789	1
18	15106987	15464370	357383	1
18	63945579	64046329	100750	1
18	66837266	67182157	344891	1
19	61591203	62455789	864586	3
19	52200671	52832739	632068	2
19	3854325	4194143	339818	1
19	5915722	6033208	117486	1
19	12506519	12732195	225676	1
19	14788994	14957020	168026	1
19	46132346	46256386	124040	1
19	47379297	47511441	132144	1
19	55704333	55772253	67920	1
20	70680394	71615622	935228	3
20	39628946	39783357	154411	2
20	2904384	3020956	116572	1
20	32347911	32504267	156356	1
20	46424799	46495170	70371	1
20	46796250	46875702	79452	1
20	54153583	54241251	87668	1
21	36563095	36798916	235821	4
21	11963796	12449340	485544	1
21	19192398	19278590	86192	1
21	46838552	46987344	148792	1
21	62930218	63047107	116889	1
22	14604623	14689380	84757	1
22	23261616	23357142	95526	1
22	50103304	50272048	168744	1
22	52360808	52649658	288850	1
22	60024564	60193667	169103	1
23	2071807	2490261	418454	2
23	403349	876205	472856	1
24	60953858	62183185	1229327	2
24	2557469	2648534	91065	1
24	3315348	4054458	739110	1
24	9548764	9722054	173290	1
24	59199354	59684957	485603	1

Chromosome	Start	End	Length	Number of animals in intersection
25	42211613	42840304	628691	4
25	28064452	28195933	131481	1
25	38139396	39245687	1106291	1
26	460069	1082309	622240	3
26	2472972	2995869	522897	2
26	46005602	46247355	241753	2
26	58230	192283	134053	1
26	22631801	22907589	275788	1
26	24729713	25124200	394487	1
26	45450183	45532222	82039	1
26	48202778	48355001	152223	1
26	49722383	50076815	354432	1
26	51077585	51249587	172002	1
27	1564755	1790700	225945	6
27	25332572	25444255	111683	2
27	3465982	3733606	267624	1
27	5100808	5267033	166225	1
27	17534126	17683471	149345	1
27	26153207	26259879	106672	1
27	44465373	44588204	122831	1
28	40262942	40411181	148239	2
28	24128889	24498183	369294	1

Supplementary Table 3: Descriptive statistics of FROH, FROH>2Mb and FG metrics, per breed

Breed	N	Metric	Minimum	Q1	Median	Mean	Standard deviation	Q3	Maximum
ABR	49	F_G	0.0232	0.0298	0.0330	0.0429	0.0388	0.0387	0.2542
ABR	49	F_{ROH}	0.0022	0.0057	0.0081	0.0184	0.0407	0.0120	0.2401
ABR	49	$F_{ROH>2Mb}$	0.0006	0.0018	0.0036	0.0154	0.0431	0.0080	0.2364

Breed	N	Metric	Minimum	Q1	Median	Mean	Standard deviation	Q3	Maximum
AND	6	F_G	0.0929	0.1065	0.1073	0.1105	0.0130	0.1151	0.1318
AND	6	F_{ROH}	0.0531	0.0635	0.0652	0.0695	0.0135	0.0756	0.0915
AND	6	$F_{ROH>2Mb}$	0.0356	0.0373	0.0401	0.0451	0.0117	0.0495	0.0655
CAM	37	F_G	0.0132	0.0237	0.0332	0.0408	0.0278	0.0417	0.1331
CAM	37	F_{ROH}	0.0005	0.0021	0.0047	0.0168	0.0256	0.0172	0.1021
CAM	37	$F_{ROH>2Mb}$	0.0009	0.0016	0.0074	0.0180	0.0269	0.0158	0.0991
DIA	14	F_G	0.1268	0.1307	0.1423	0.1877	0.1004	0.1795	0.4762
DIA	14	F_{ROH}	0.0823	0.0899	0.1044	0.1502	0.1052	0.1430	0.4512
DIA	14	$F_{ROH>2Mb}$	0.0512	0.0568	0.0666	0.1179	0.1076	0.1110	0.4221
GAL	23	F_G	0.0148	0.0195	0.0216	0.0239	0.0089	0.0251	0.0512
GAL	23	F_{ROH}	0.0006	0.0014	0.0029	0.0046	0.0070	0.0044	0.0325
GAL	23	$F_{ROH>2Mb}$	0.0008	0.0009	0.0014	0.0043	0.0079	0.0027	0.0303
GAZ	4	F_G	0.0394	0.0423	0.0438	0.0500	0.0155	0.0515	0.0730
GAZ	4	F_{ROH}	0.0168	0.0208	0.0251	0.0257	0.0082	0.0301	0.0358
GAZ	4	$F_{ROH>2Mb}$	0.0108	0.0127	0.0168	0.0165	0.0053	0.0206	0.0217
GOG	12	F_G	0.0074	0.0120	0.0211	0.0292	0.0303	0.0246	0.1094
GOG	12	F_{ROH}	0.0002	0.0011	0.0030	0.0168	0.0337	0.0057	0.1076
GOG	12	$F_{ROH>2Mb}$	0.0008	0.0019	0.0037	0.0242	0.0402	0.0276	0.1060
GUE	16	F_G	0.0012	0.0187	0.0483	0.0595	0.0562	0.0725	0.2015

**Breed**	**N**	**Metric**	**Minimum**	**Q1**	**Median**	**Mean**	**Standard deviation**	**Q3**	**Maximum**
GUE	16	F_{ROH}	0.0007	0.0030	0.0375	0.0512	0.0601	0.0695	0.1996
GUE	16	$F_{ROH>2Mb}$	0.0009	0.0044	0.0502	0.0578	0.0612	0.0714	0.1996
GUM	39	F_G	0.0195	0.0243	0.0285	0.0352	0.0326	0.0319	0.2145
GUM	39	F_{ROH}	0.0014	0.0060	0.0075	0.0158	0.0340	0.0106	0.2037
GUM	39	$F_{ROH>2Mb}$	0.0008	0.0032	0.0041	0.0134	0.0355	0.0065	0.2009
KAR	19	F_G	0.0101	0.0193	0.0217	0.0257	0.0168	0.0282	0.0861
KAR	19	F_{ROH}	0.0004	0.0014	0.0028	0.0066	0.0138	0.0039	0.0586
KAR	19	$F_{ROH>2Mb}$	0.0008	0.0009	0.0019	0.0084	0.0174	0.0033	0.0569
KEF	44	F_G	0.0260	0.0362	0.0442	0.0703	0.0642	0.0598	0.2911
KEF	44	F_{ROH}	0.0037	0.0150	0.0216	0.0497	0.0657	0.0419	0.2816
KEF	44	$F_{ROH>2Mb}$	0.0021	0.0091	0.0143	0.0433	0.0660	0.0332	0.2734
LND	29	F_G	0.0483	0.0755	0.1067	0.1343	0.0884	0.1527	0.4234
LND	29	F_{ROH}	0.0128	0.0256	0.0415	0.0794	0.0924	0.0838	0.3992
LND	29	$F_{ROH>2Mb}$	0.0019	0.0103	0.0136	0.0618	0.0942	0.0689	0.3890
MAA	18	F_G	0.0122	0.0198	0.0234	0.0305	0.0233	0.0295	0.1127
MAA	18	F_{ROH}	0.0003	0.0017	0.0032	0.0123	0.0235	0.0085	0.0956
MAA	18	$F_{ROH>2Mb}$	0.0009	0.0035	0.0054	0.0177	0.0281	0.0138	0.0919
MAN	3	F_G	0.0368	0.0377	0.0387	0.0387	0.0027	0.0396	0.0406
MAN	3	F_{ROH}	0.0181	0.0203	0.0225	0.0225	0.0062	0.0247	0.0269

Breed	N	Metric	Minimum	Q1	Median	Mean	Standard deviation	Q3	Maximum
MAN	3	$F_{ROH>2Mb}$	0.0074	0.0114	0.0154	0.0154	0.0113	0.0194	0.0234
MEN	19	F_G	0.1464	0.1686	0.1797	0.1865	0.0447	0.1889	0.3599
MEN	19	F_{ROH}	0.0866	0.1035	0.1089	0.1209	0.0479	0.1205	0.3083
MEN	19	$F_{ROH>2Mb}$	0.0493	0.0681	0.0726	0.0856	0.0505	0.0905	0.2828
MSH	22	F_G	0.0641	0.0723	0.0820	0.1076	0.0486	0.1451	0.2189
MSH	22	F_{ROH}	0.0182	0.0304	0.0347	0.0634	0.0498	0.1106	0.1872
MSH	22	$F_{ROH>2Mb}$	0.0042	0.0098	0.0159	0.0442	0.0496	0.0884	0.1768
NAI	14	F_G	0.0173	0.0253	0.0309	0.0327	0.0134	0.0331	0.0718
NAI	14	F_{ROH}	0.0032	0.0082	0.0157	0.0185	0.0131	0.0246	0.0536
NAI	14	$F_{ROH>2Mb}$	0.0023	0.0051	0.0121	0.0154	0.0131	0.0222	0.0513
NSJ	6	F_G	0.0621	0.0877	0.1133	0.1133	0.0723	0.1388	0.1644
NSJ	6	F_{ROH}	0.0218	0.0529	0.0839	0.0839	0.0878	0.1150	0.1460
NSJ	6	$F_{ROH>2Mb}$	0.0093	0.0419	0.0744	0.0744	0.0919	0.1069	0.1394
PAF	4	F_G	0.0335	0.0398	0.0541	0.0549	0.0207	0.0692	0.0778
PAF	4	F_{ROH}	0.0184	0.0224	0.0413	0.0420	0.0246	0.0610	0.0671
PAF	4	$F_{ROH>2Mb}$	0.0072	0.0162	0.0359	0.0359	0.0271	0.0556	0.0645
PRW	19	F_G	0.0060	0.0276	0.0419	0.0621	0.0850	0.0637	0.3699
PRW	19	F_{ROH}	0.0024	0.0113	0.0343	0.0523	0.0857	0.0573	0.3614
PRW	19	$F_{ROH>2Mb}$	0.0021	0.0100	0.0316	0.0504	0.0850	0.0557	0.3574

Breed	N	Metric	Minimum	Q1	Median	Mean	Standard deviation	Q3	Maximum
RSK	19	F_G	0.0160	0.0189	0.0250	0.0479	0.0484	0.0438	0.1802
RSK	19	F_{ROH}	0.0008	0.0017	0.0067	0.0303	0.0496	0.0255	0.1684
RSK	19	$F_{ROH>2Mb}$	0.0014	0.0081	0.0212	0.0504	0.0578	0.0869	0.1655
SDN	22	F_G	0.0138	0.0181	0.0245	0.0264	0.0112	0.0318	0.0589
SDN	22	F_{ROH}	0.0007	0.0019	0.0073	0.0090	0.0087	0.0120	0.0358
SDN	22	$F_{ROH>2Mb}$	0.0020	0.0035	0.0078	0.0097	0.0086	0.0116	0.0328
SEA	48	F_G	0.0162	0.0349	0.0418	0.0808	0.0634	0.1503	0.2356
SEA	48	F_{ROH}	0.0007	0.0036	0.0076	0.0450	0.0611	0.0678	0.2080
SEA	48	$F_{ROH>2Mb}$	0.0009	0.0029	0.0094	0.0428	0.0609	0.0558	0.1979
SEB	21	F_G	0.0092	0.0198	0.0247	0.0230	0.0066	0.0283	0.0332
SEB	21	F_{ROH}	0.0016	0.0036	0.0056	0.0070	0.0051	0.0077	0.0202
SEB	21	$F_{ROH>2Mb}$	0.0010	0.0020	0.0031	0.0053	0.0053	0.0067	0.0191
SHL	19	F_G	0.0113	0.0153	0.0185	0.0218	0.0123	0.0225	0.0648
SHL	19	F_{ROH}	0.0013	0.0020	0.0039	0.0071	0.0113	0.0067	0.0499
SHL	19	$F_{ROH>2Mb}$	0.0009	0.0024	0.0030	0.0064	0.0114	0.0036	0.0461
SNJ	20	F_G	0.0076	0.0123	0.0205	0.0195	0.0082	0.0237	0.0321
SNJ	20	F_{ROH}	0.0016	0.0040	0.0054	0.0066	0.0041	0.0072	0.0156
SNJ	20	$F_{ROH>2Mb}$	0.0008	0.0026	0.0038	0.0052	0.0044	0.0057	0.0150
SOF	22	F_G	0.1587	0.1883	0.2043	0.2261	0.0630	0.2403	0.4151

Breed	N	Metric	Minimum	Q1	Median	Mean	Standard deviation	Q3	Maximum
SOF	22	F_{ROH}	0.1042	0.1356	0.1590	0.1768	0.0680	0.1941	0.3838
SOF	22	$F_{ROH>2Mb}$	0.0702	0.0993	0.1199	0.1417	0.0693	0.1604	0.3509
SOU	8	F_G	0.1077	0.1214	0.1337	0.1286	0.0138	0.1358	0.1467
SOU	8	F_{ROH}	0.0608	0.0754	0.0840	0.0807	0.0112	0.0863	0.0969
SOU	8	$F_{ROH>2Mb}$	0.0419	0.0443	0.0504	0.0538	0.0116	0.0633	0.0710
THY	9	F_G	0.0604	0.0680	0.0710	0.1229	0.1214	0.0990	0.3950
THY	9	F_{ROH}	0.0248	0.0301	0.0364	0.0857	0.1304	0.0491	0.3806
THY	9	$F_{ROH>2Mb}$	0.0069	0.0115	0.0160	0.0685	0.1354	0.0296	0.3746
WAD	50	F_G	0.0277	0.0643	0.0742	0.0830	0.0521	0.0821	0.3320
WAD	50	F_{ROH}	0.0006	0.0110	0.0169	0.0317	0.0526	0.0244	0.2901
WAD	50	$F_{ROH>2Mb}$	0.0009	0.0041	0.0066	0.0274	0.0566	0.0213	0.2870
WYG	39	F_G	0.0191	0.0259	0.0294	0.0361	0.0245	0.0348	0.1506
WYG	39	F_{ROH}	0.0010	0.0034	0.0050	0.0144	0.0253	0.0109	0.1335
WYG	39	$F_{ROH>2Mb}$	0.0008	0.0015	0.0029	0.0126	0.0257	0.0077	0.1321

2 Supplementary Figures

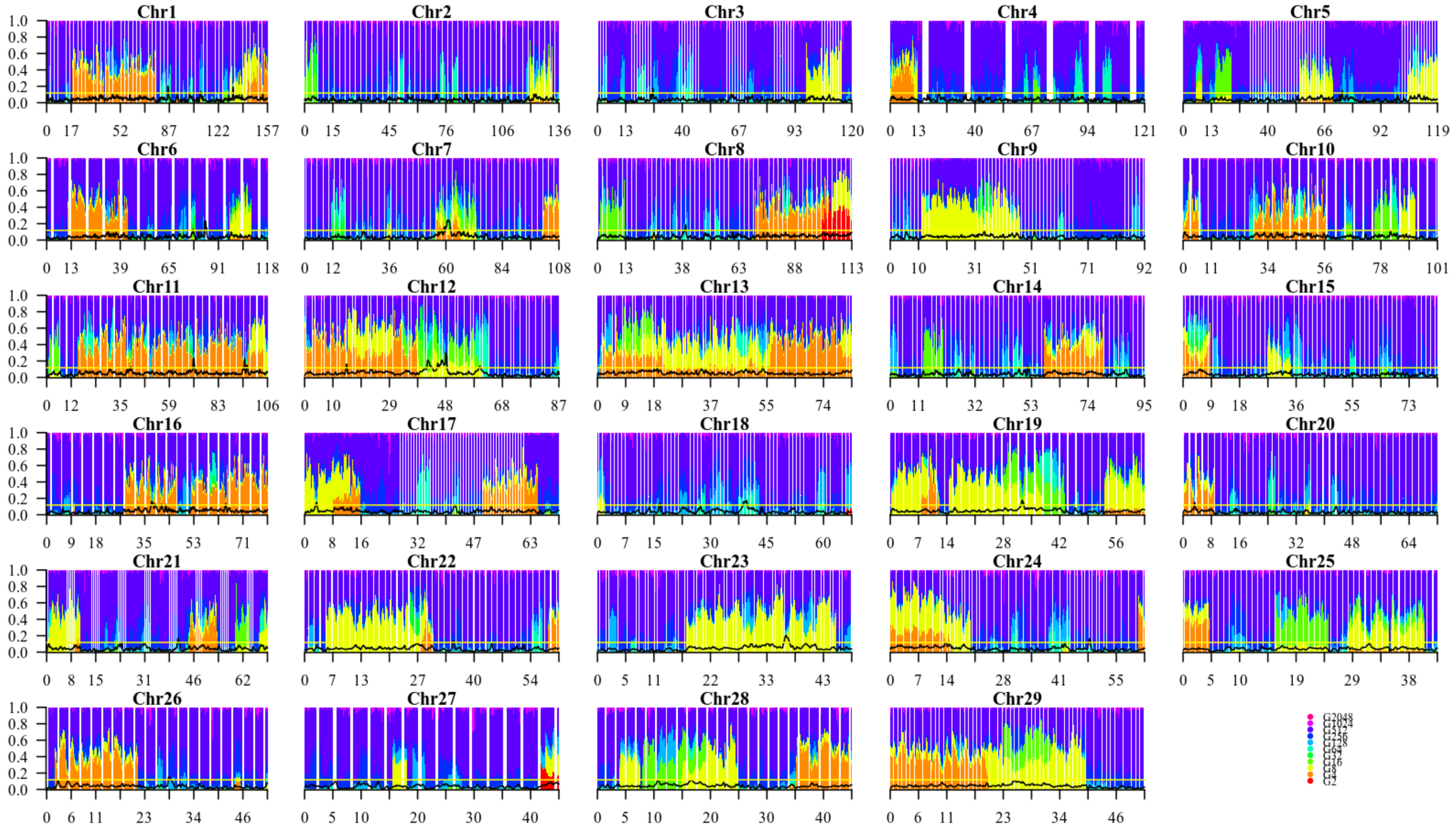

Supplementary Figure 1-1: Local homozygosity-by-descent (HBD) state probabilities for each chromosome for Abergelle. The black line is the mean HBD state probability at each marker. The yellow line is the 99th percentile of marker HBD state probabilities across the genome for the breed.

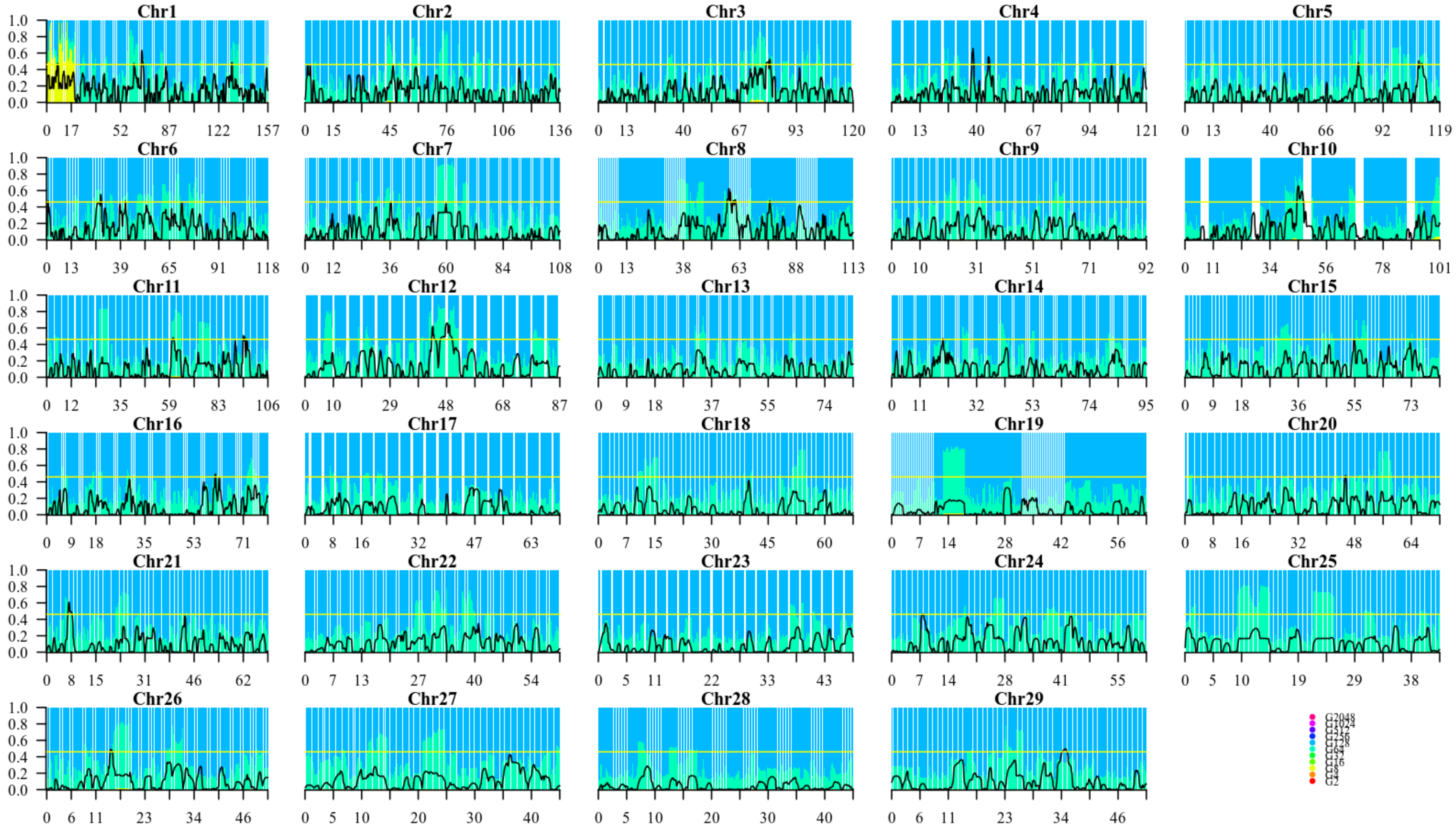

Supplementary Figure 1-2: Local homozygosity-by-descent (HBD) state probabilities for each chromosome for Androy The black line is the mean HBD state probability at each marker. The yellow line is the 99th percentile of marker HBD state probabilities across the genome for the breed.

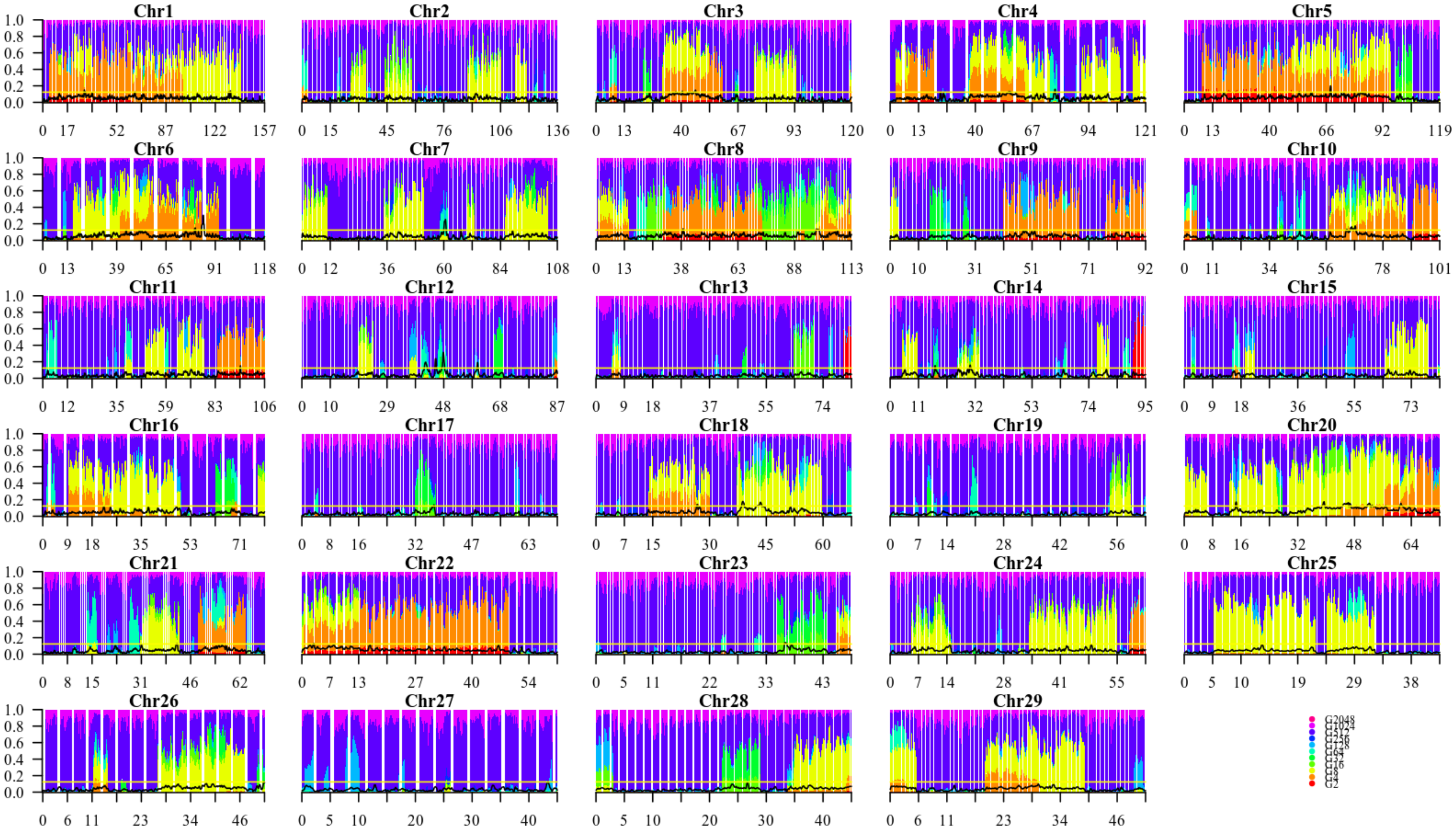

Supplementary Figure 1-3: Local homozygosity-by-descent (HBD) state probabilities for each chromosome for Cameroon. The black line is the mean HBD state probability at each marker. The yellow line is the 99th percentile of marker HBD state probabilities across the genome for the breed.

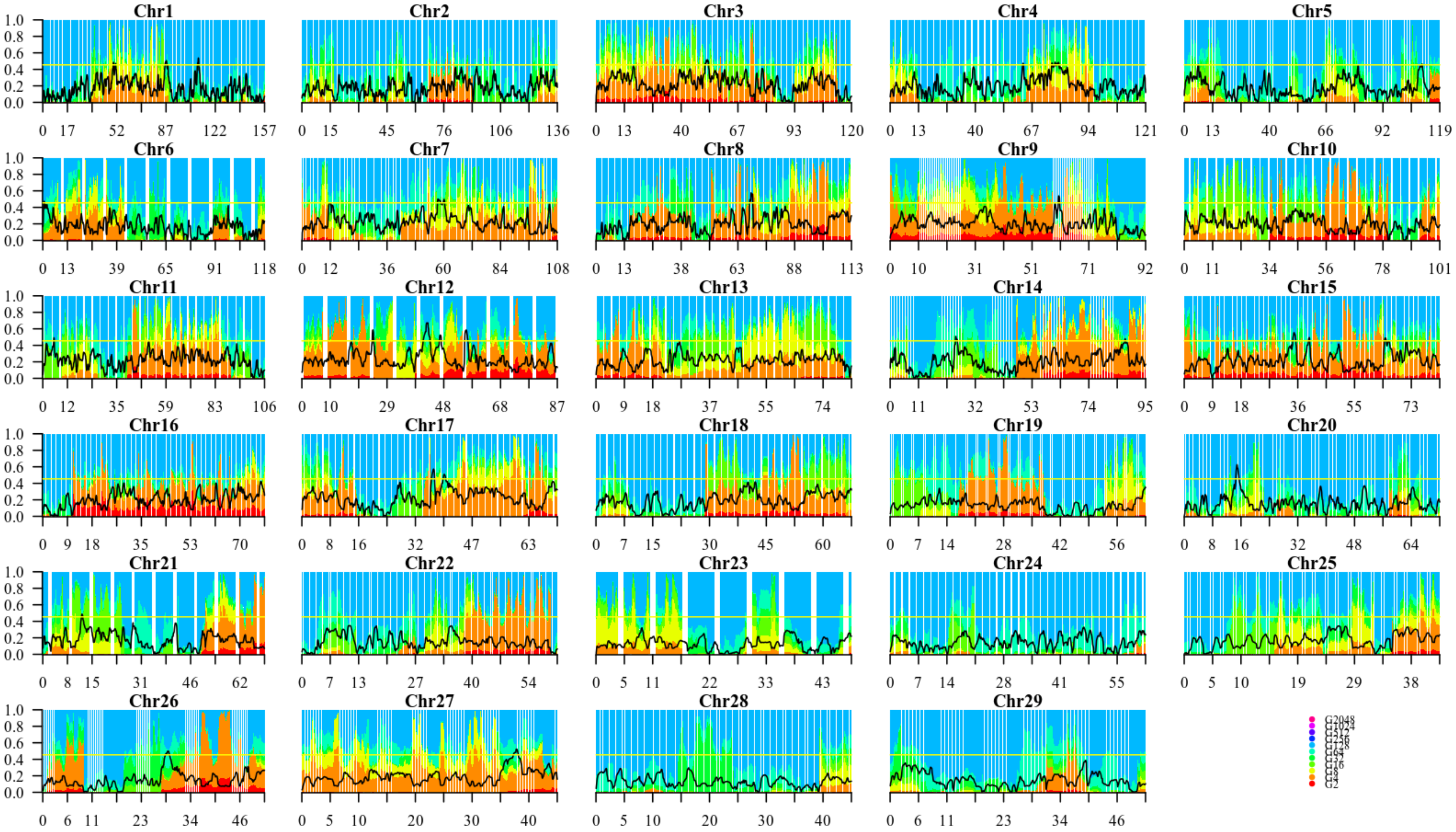

Supplementary Figure 1-4: Local homozygosity-by-descent (HBD) state probabilities for each chromosome for Diana. The black line is the mean HBD state probability at each marker. The yellow line is the 99th percentile of marker HBD state probabilities across the genome for the breed.

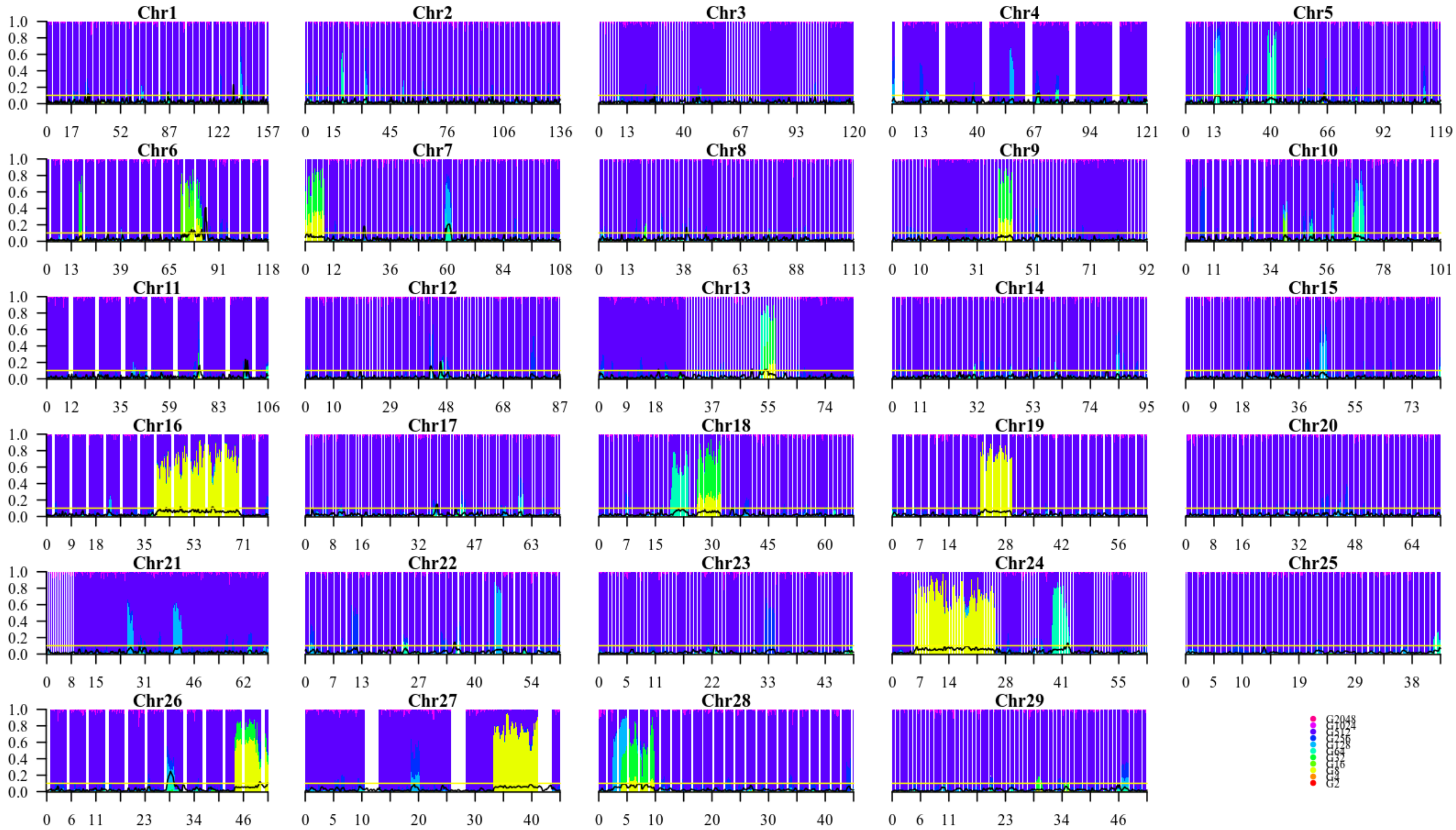

Supplementary Figure 1-5: Local homozygosity-by-descent (HBD) state probabilities for each chromosome for Galla. The black line is the mean HBD state probability at each marker. The yellow line is the 99th percentile of marker HBD state probabilities across the genome for the breed.

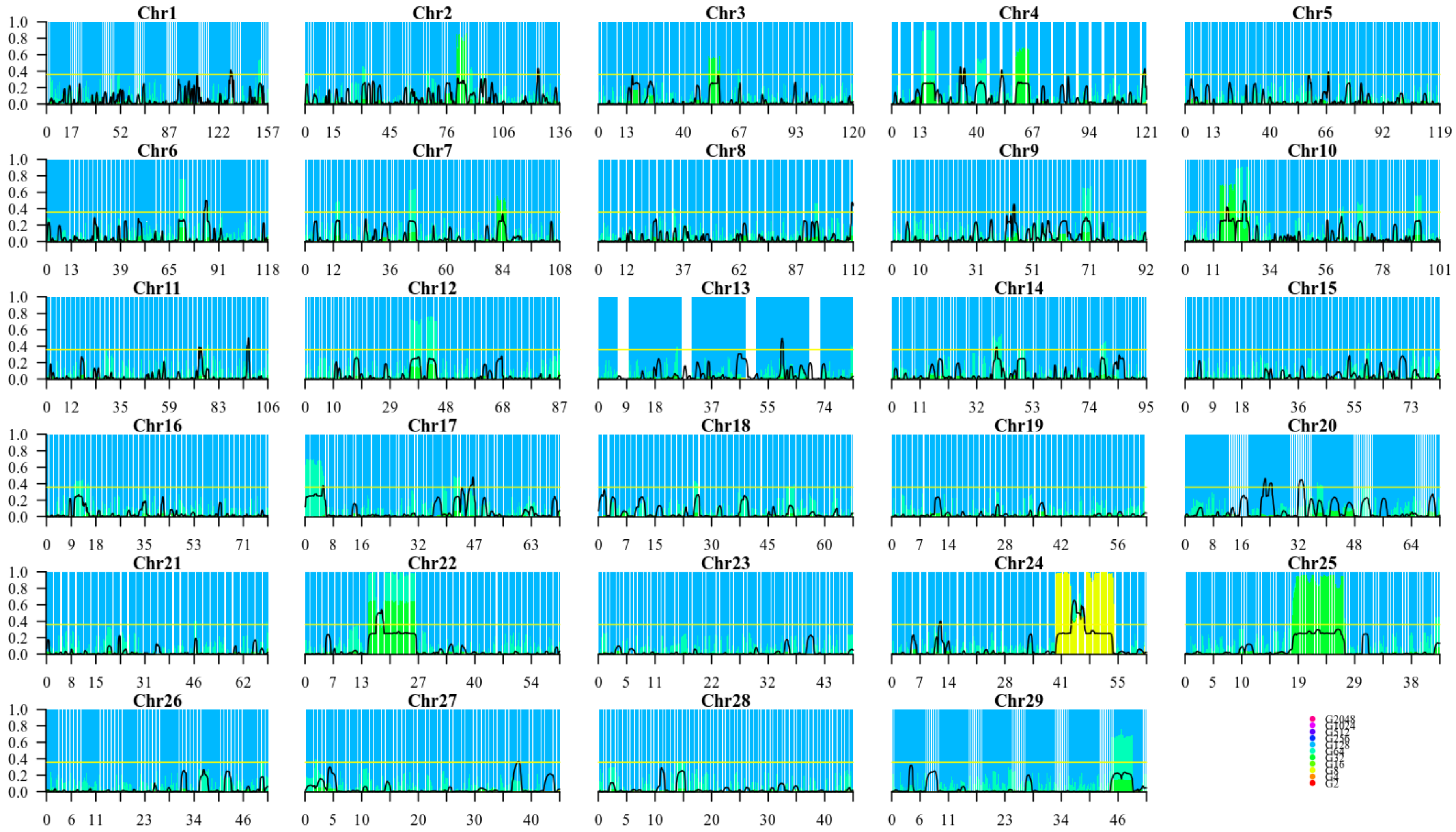

Supplementary Figure 1-6: Local homozygosity-by-descent (HBD) state probabilities for each chromosome for Gaza. The black line is the

mean HBD state probability at each marker. The yellow line is the 99th percentile of marker HBD state probabilities across the genome for the breed.

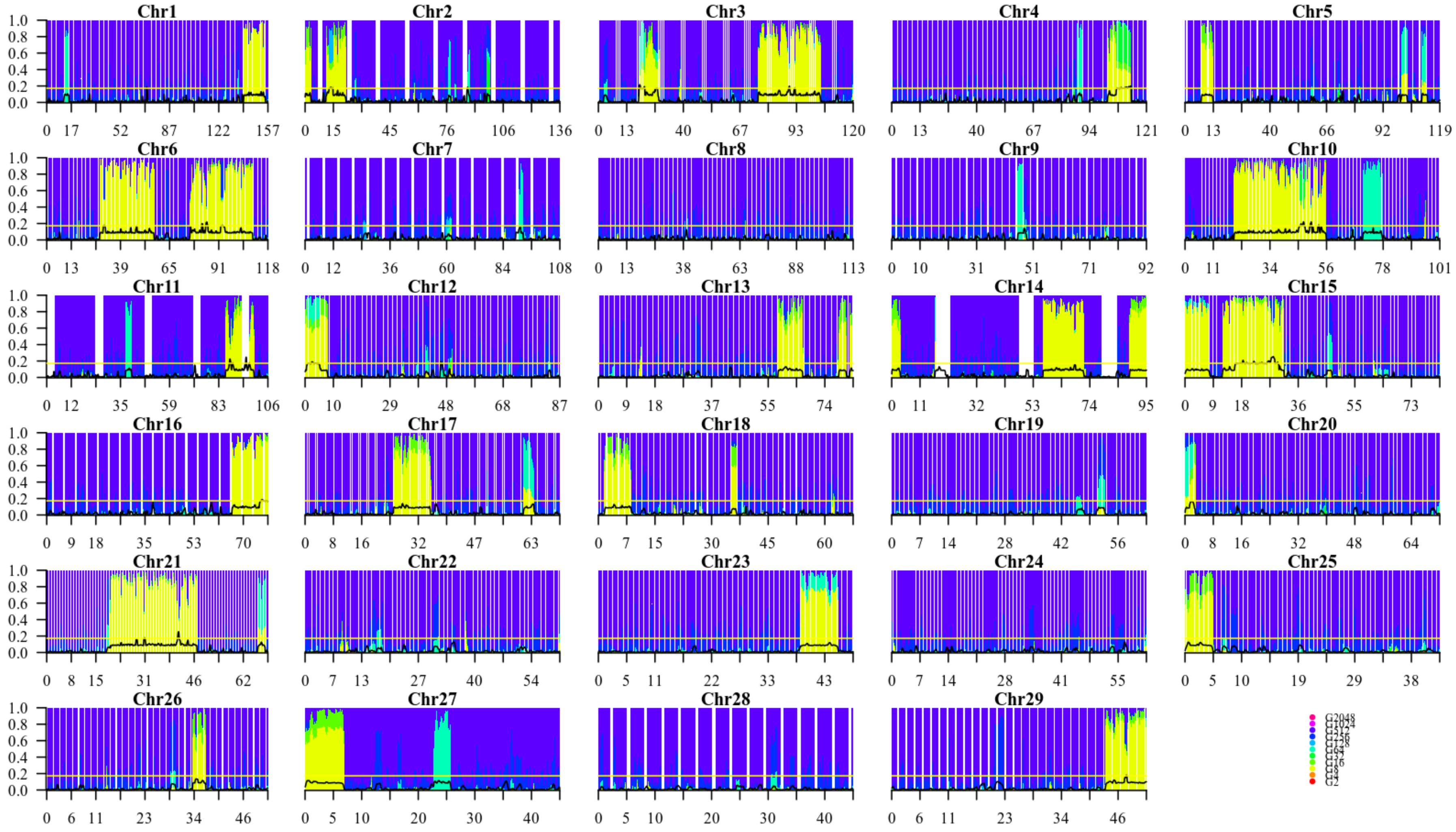

Supplementary Figure 1-7: Local homozygosity-by-descent (HBD) state probabilities for each chromosome for Gogo. The black line is the mean HBD state probability at each marker. The yellow line is the 99th percentile of marker HBD state probabilities across the genome for the breed.

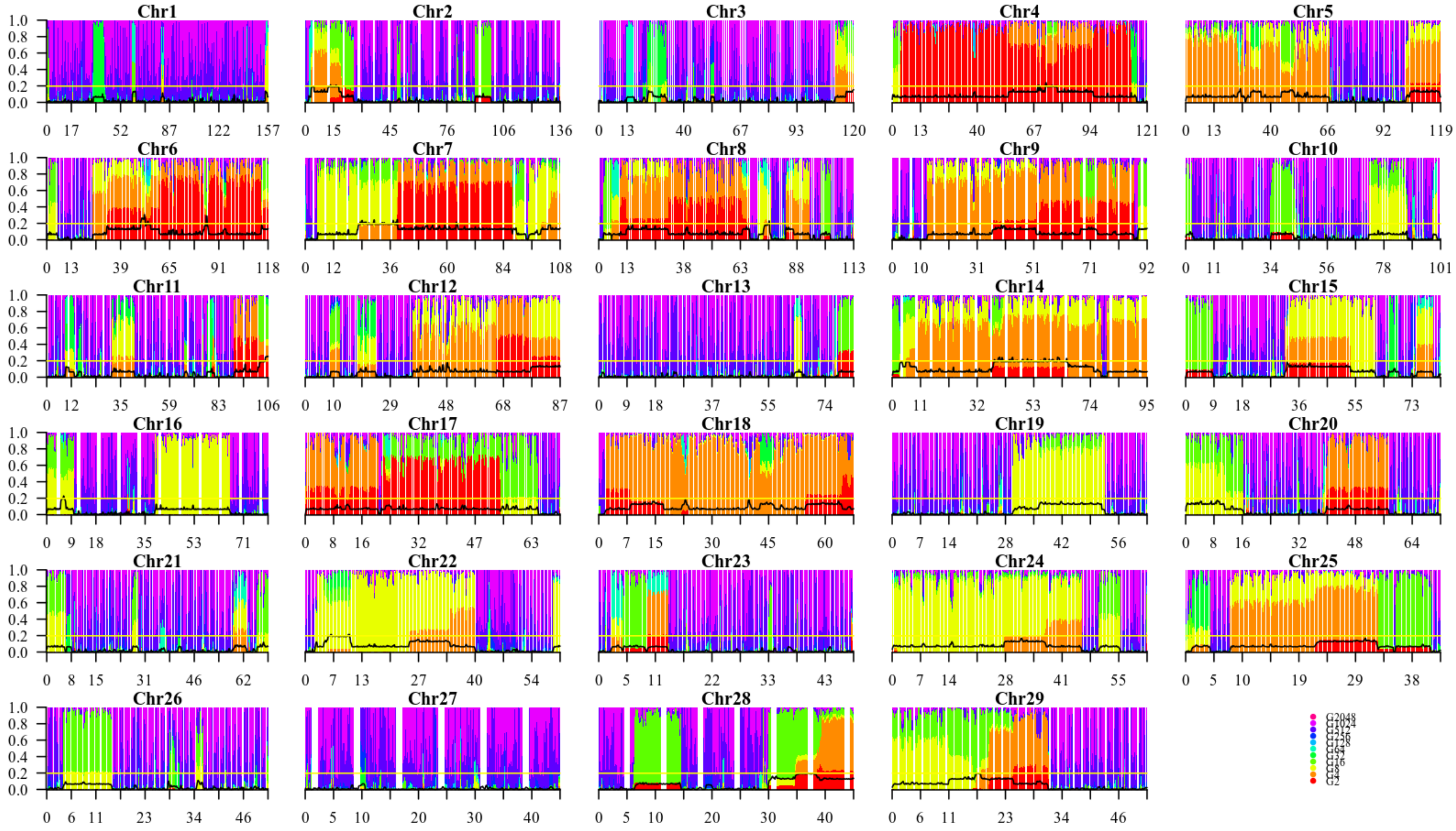

Supplementary Figure 1-8: Local homozygosity-by-descent (HBD) state probabilities for each chromosome for Guera. The black line is the mean HBD state probability at each marker. The yellow line is the 99th percentile of marker HBD state probabilities across the genome for the breed.

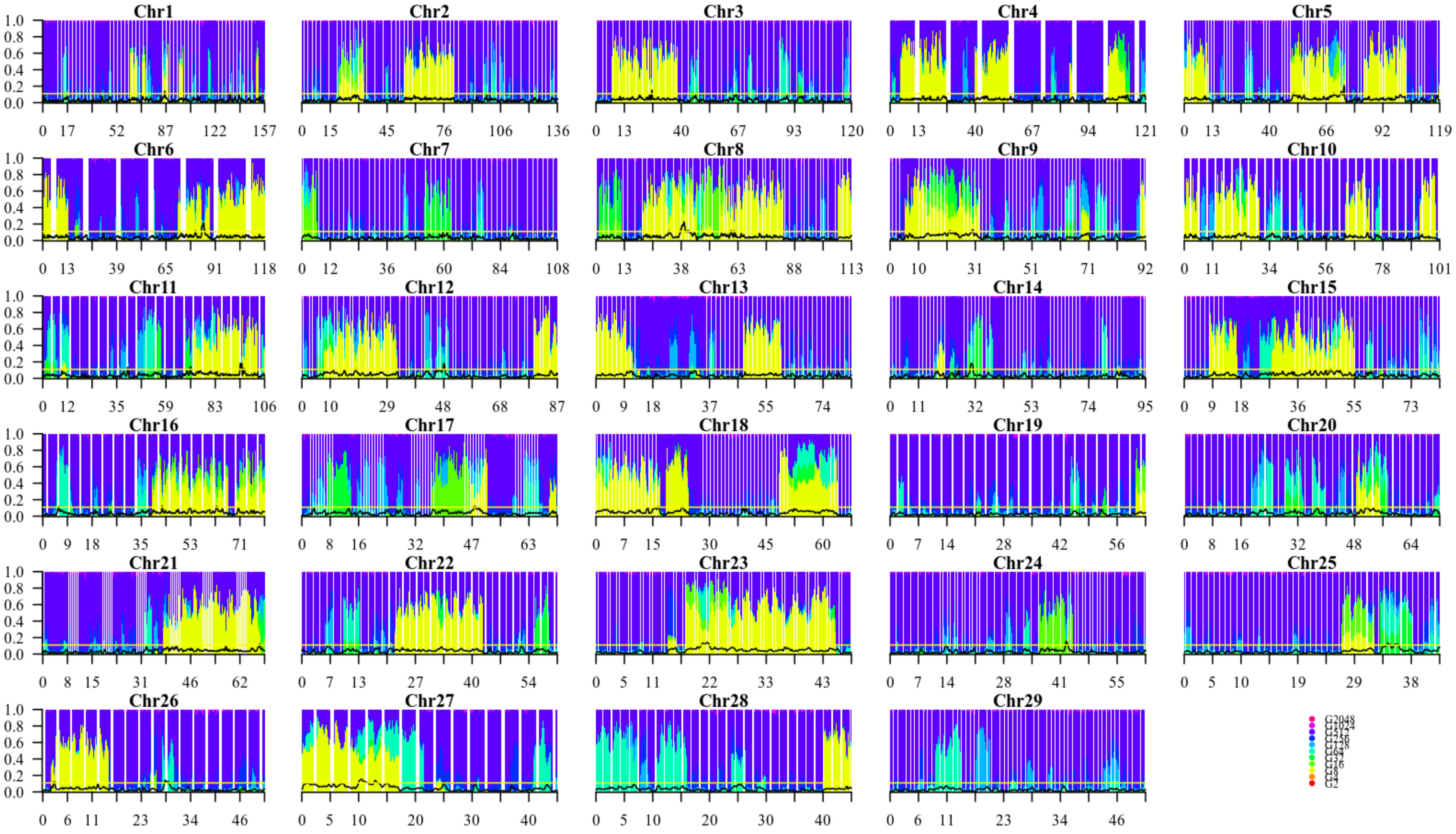

Supplementary Figure 1-9: Local homozygosity-by-descent (HBD) state probabilities for each chromosome for Gumez. The black line is the mean HBD state probability at each marker. The yellow line is the 99th percentile of marker HBD state probabilities across the genome for the breed.

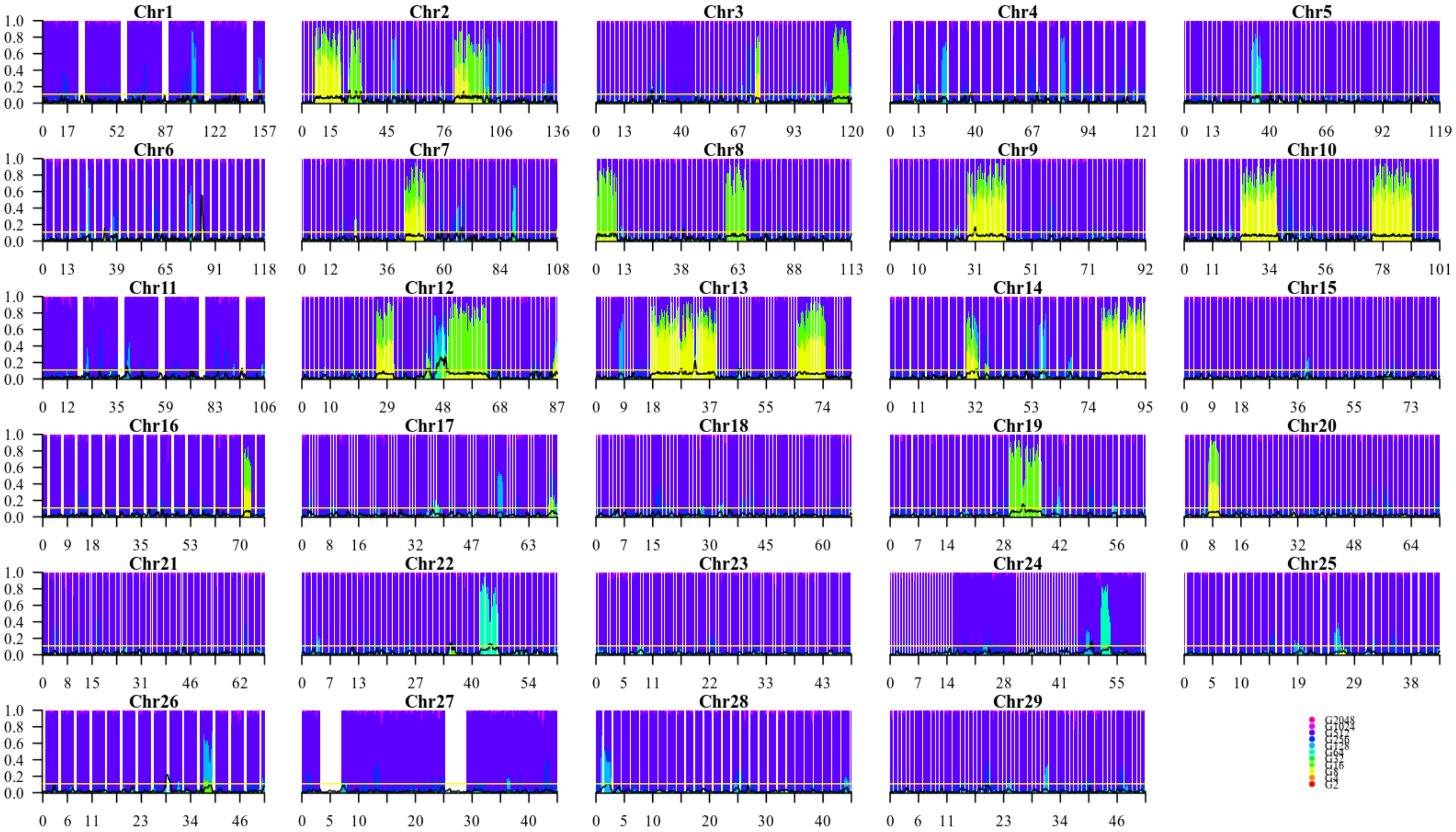

Supplementary Figure 1-10: Local homozygosity-by-descent (HBD) state probabilities for each chromosome for Karamoja. The black line is the mean HBD state probability at each marker. The yellow line is the 99th percentile of marker HBD state probabilities across the genome for the breed.

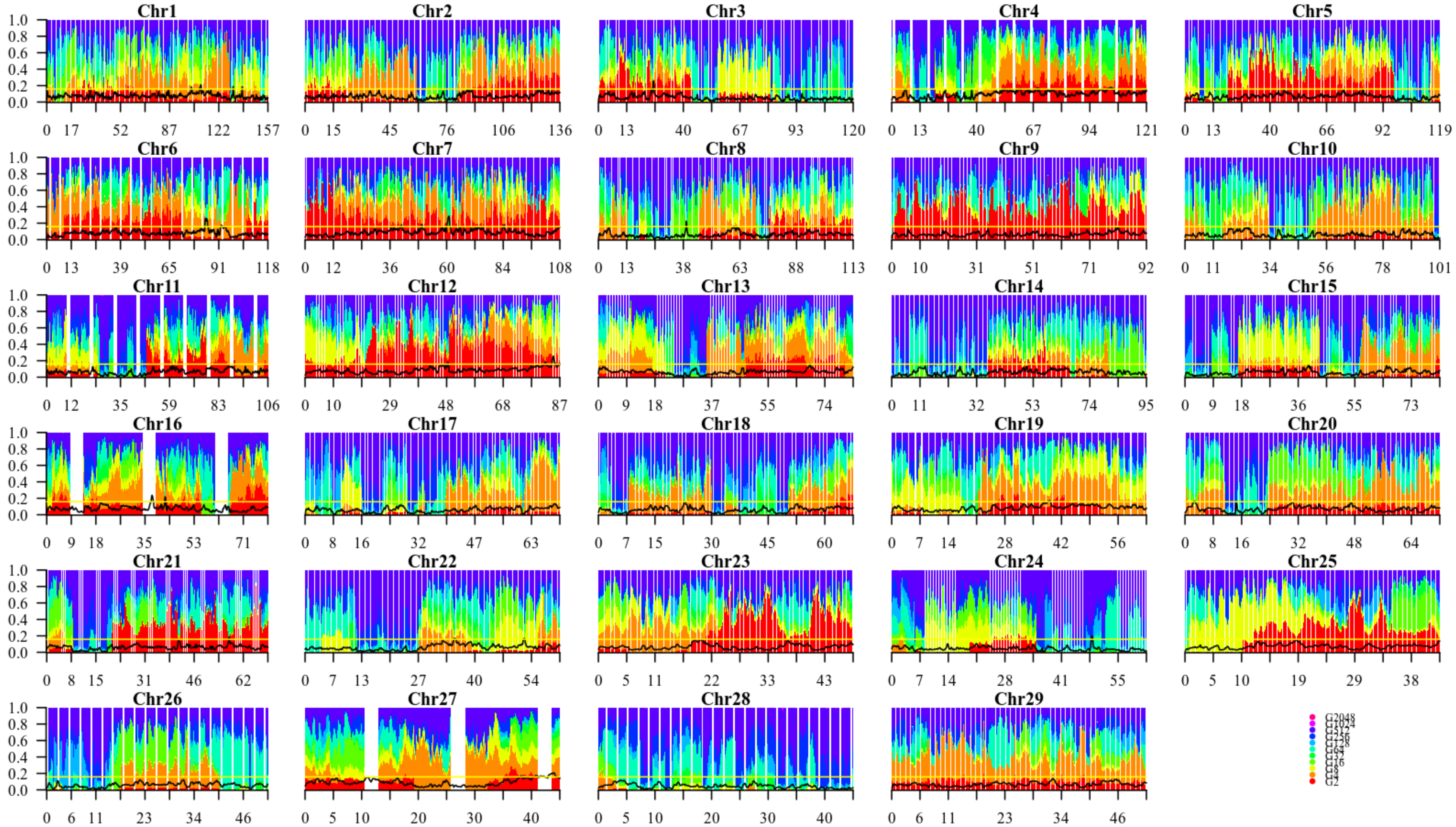

Supplementary Figure 1-11: Local homozygosity-by-descent (HBD) state probabilities for each chromosome for Keffa. The black line is the mean HBD state probability at each marker. The yellow line is the 99th percentile of marker HBD state probabilities across the genome for the breed.

Supplementary Material

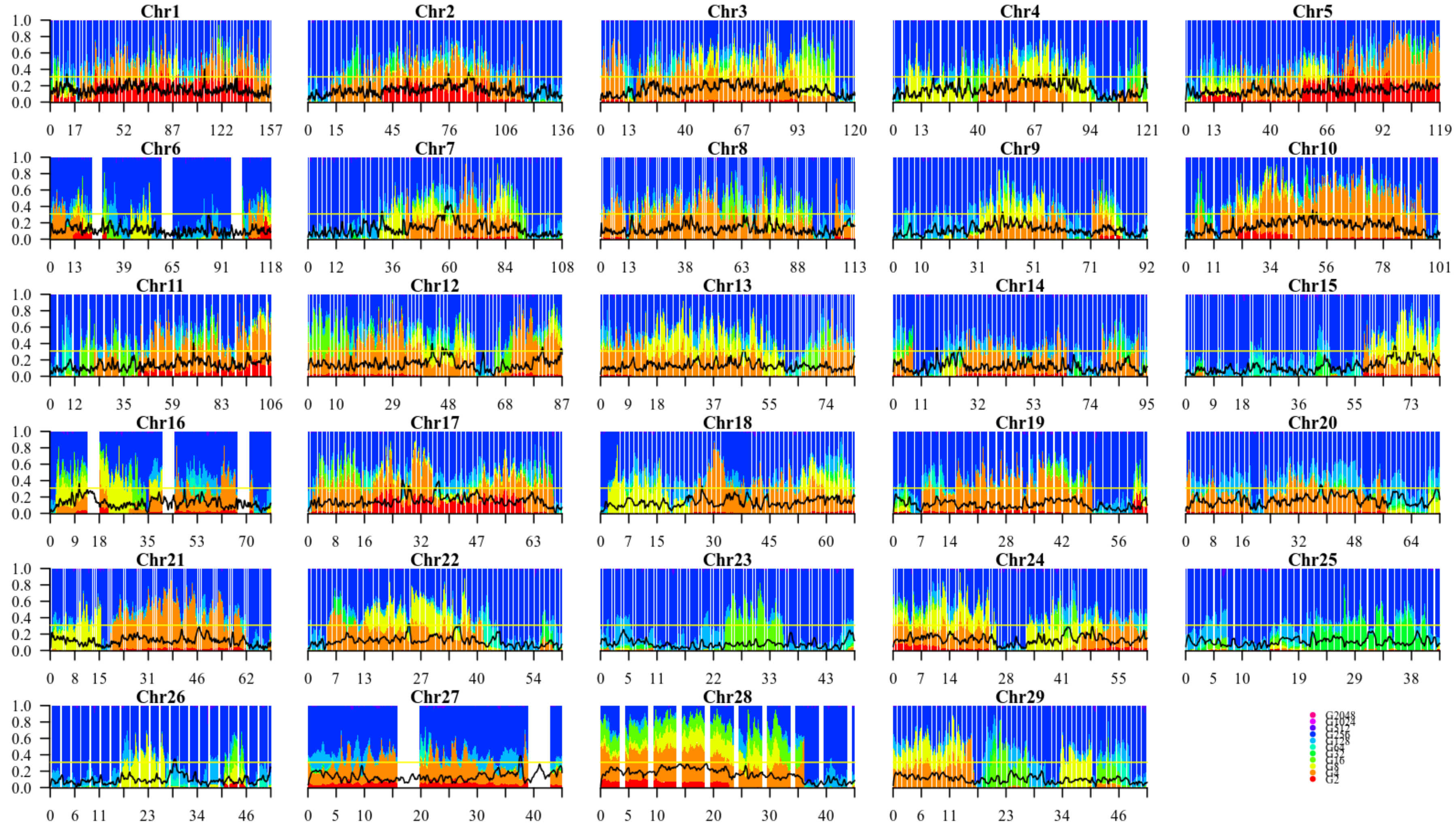

Supplementary Figure 1-12: Local homozygosity-by-descent (HBD) state probabilities for each chromosome for Landim. The black line is the mean HBD state probability at each marker. The yellow line is the 99th percentile of marker HBD state probabilities across the genome for the breed.

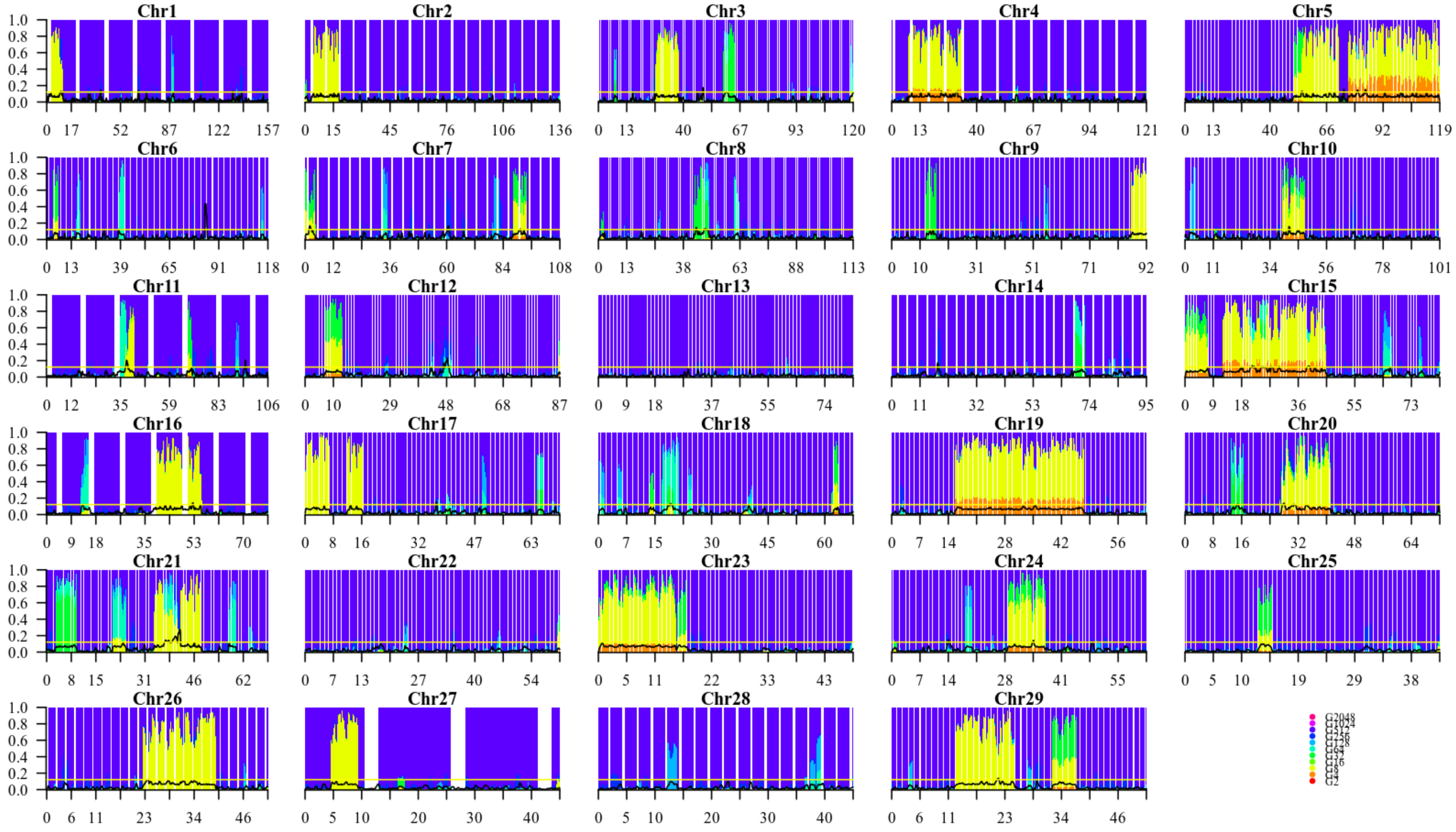

Supplementary Figure 1-13: Local homozygosity-by-descent (HBD) state probabilities for each chromosome for Maasai. The black line is the mean HBD state probability at each marker. The yellow line is the 99th percentile of marker HBD state probabilities across the genome for the breed.

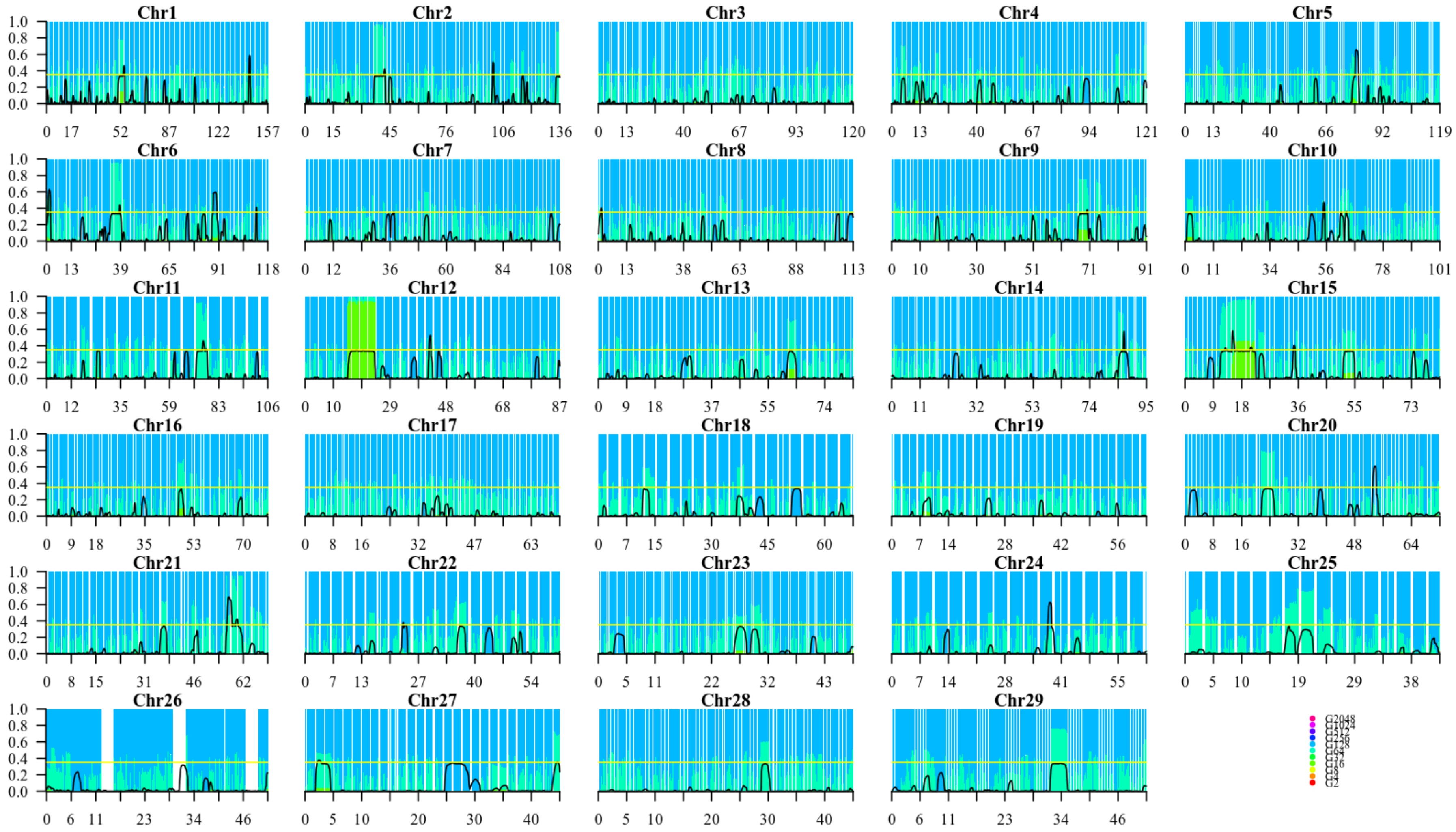

Supplementary Figure 1-14: Local homozygosity-by-descent (HBD) state probabilities for each chromosome for Manica. The black line is the mean HBD state probability at each marker. The yellow line is the 99th percentile of marker HBD state probabilities across the genome for the breed.

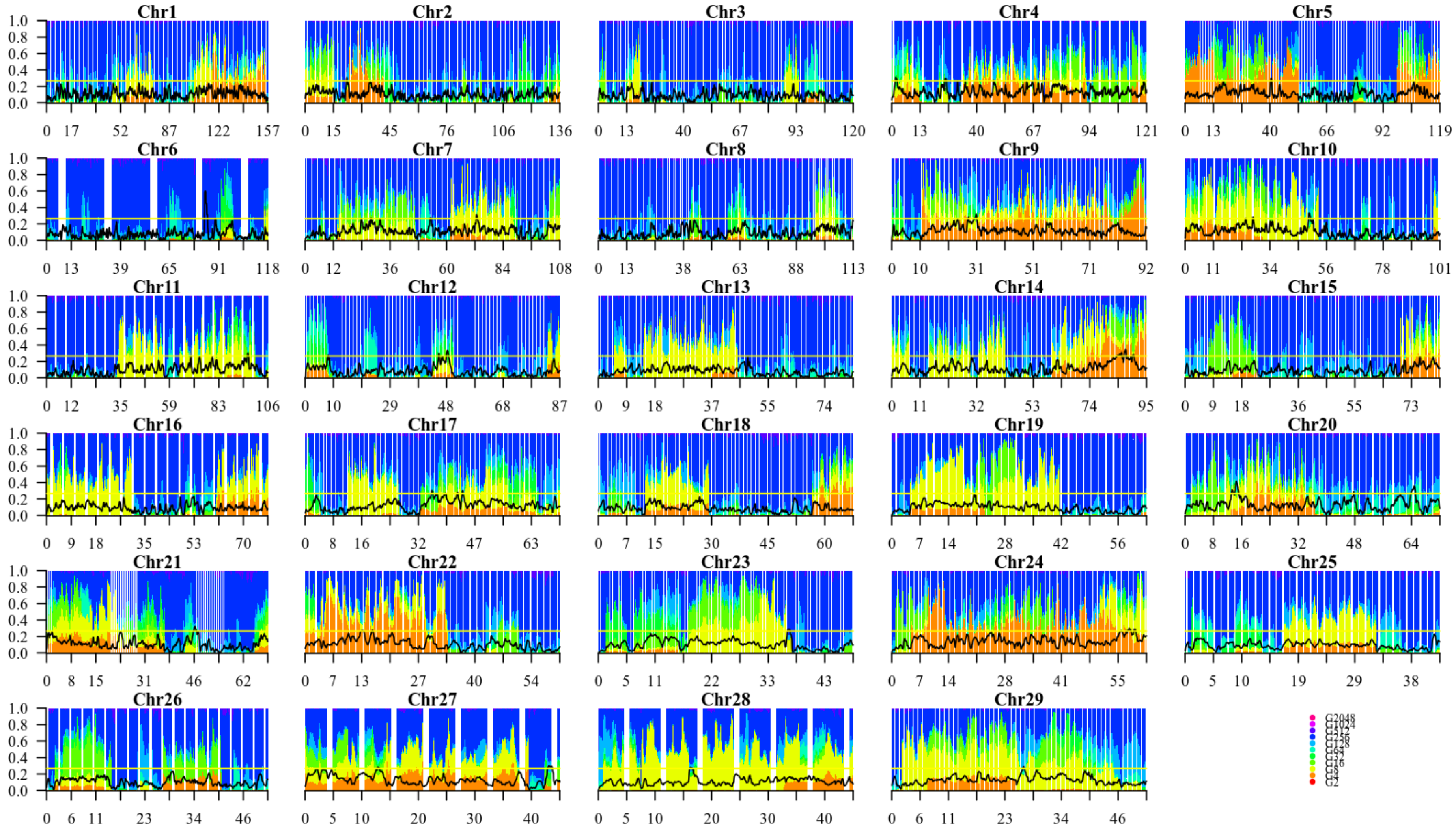

Supplementary Figure 1-15: Local homozygosity-by-descent (HBD) state probabilities for each chromosome for Mashona. The black line is the mean HBD state probability at each marker. The yellow line is the 99th percentile of marker HBD state probabilities across the genome for the breed.

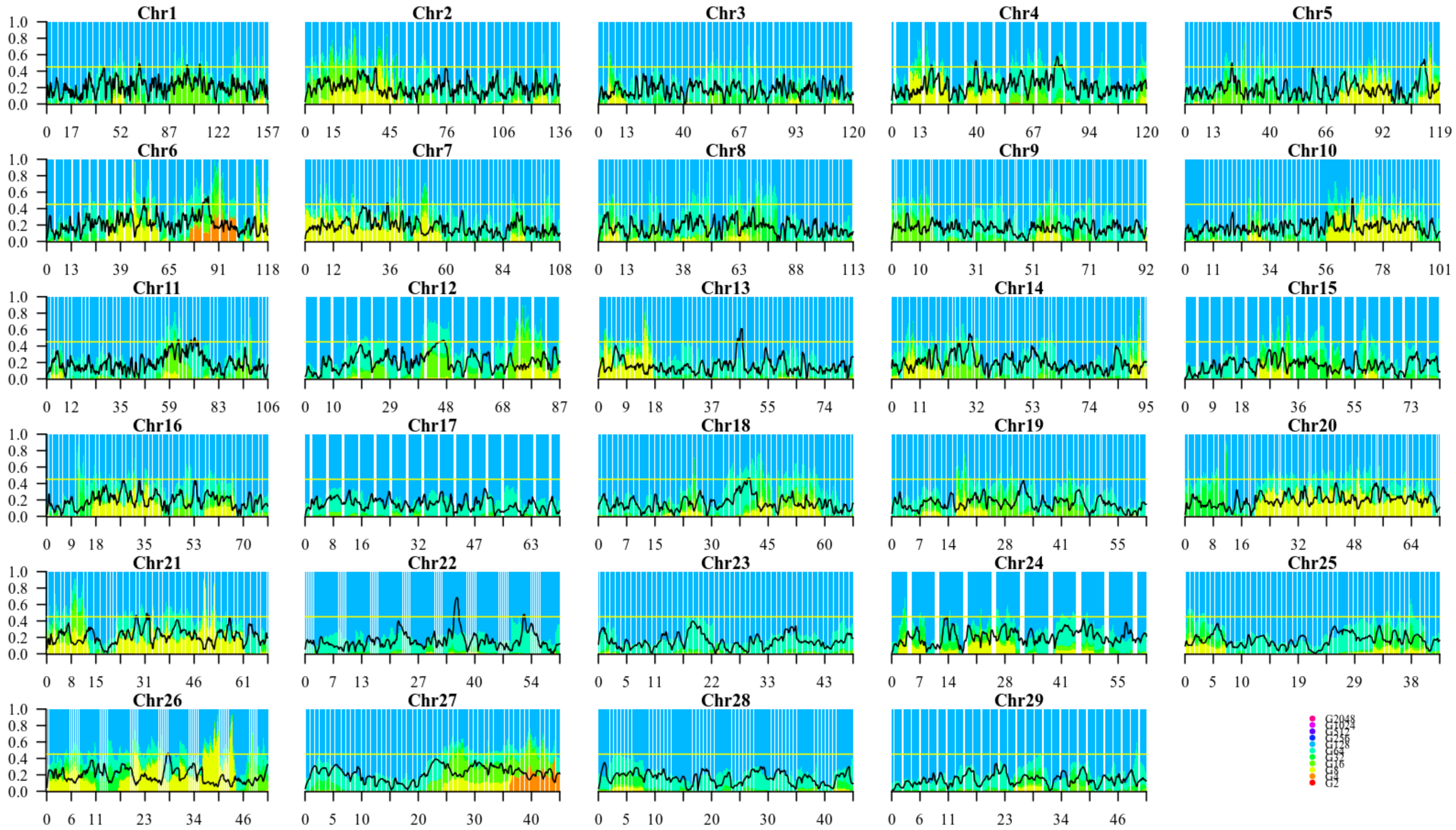

Supplementary Figure 1-16: Local homozygosity-by-descent (HBD) state probabilities for each chromosome for Menabe. The black line is

the mean HBD state probability at each marker. The yellow line is the 99th percentile of marker HBD state probabilities across the genome for the breed.

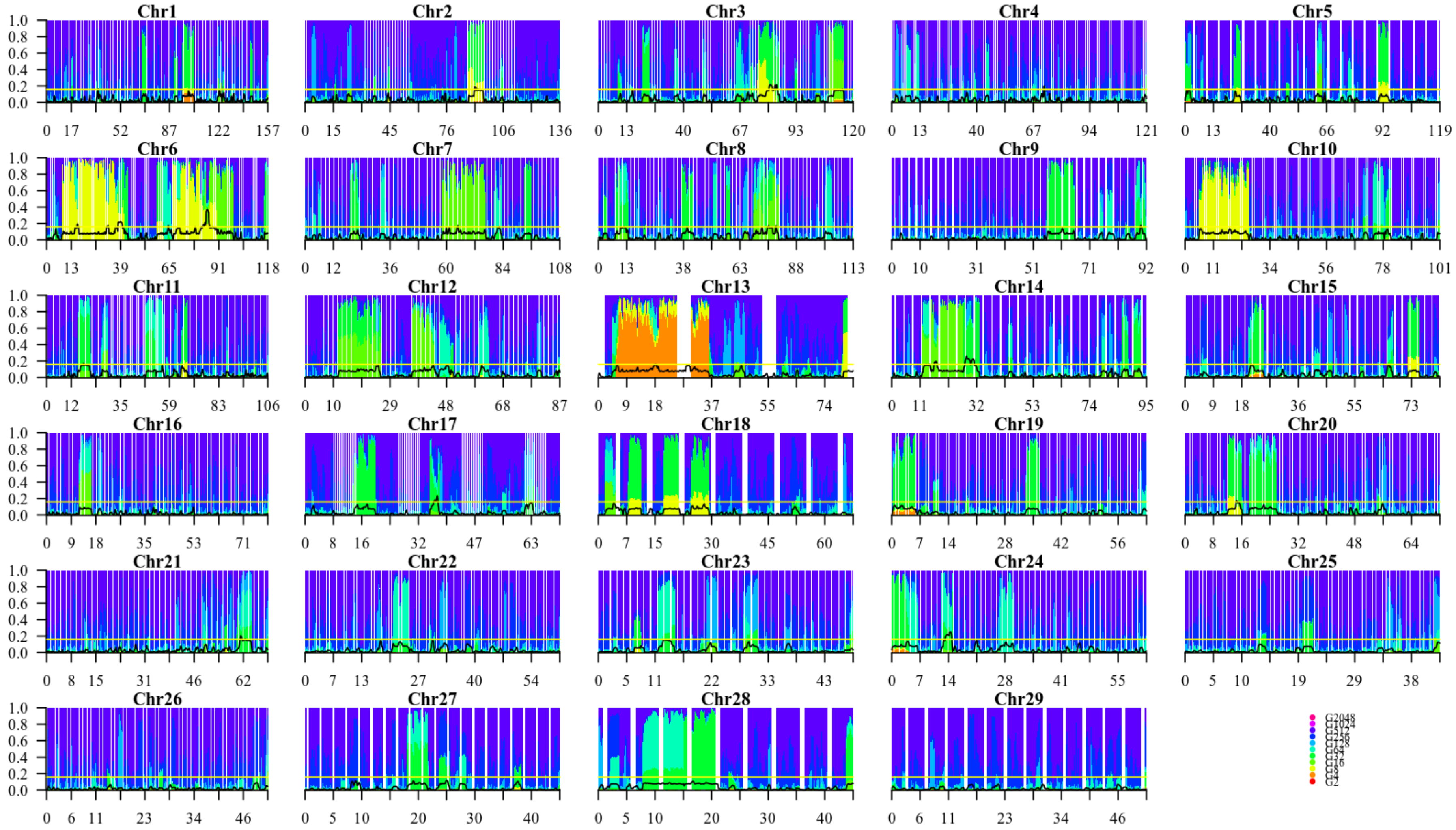

Supplementary Figure 1-17: Local homozygosity-by-descent (HBD) state probabilities for each chromosome for Naine. The black line is

the mean HBD state probability at each marker. The yellow line is the 99th percentile of marker HBD state probabilities across the genome for the breed.

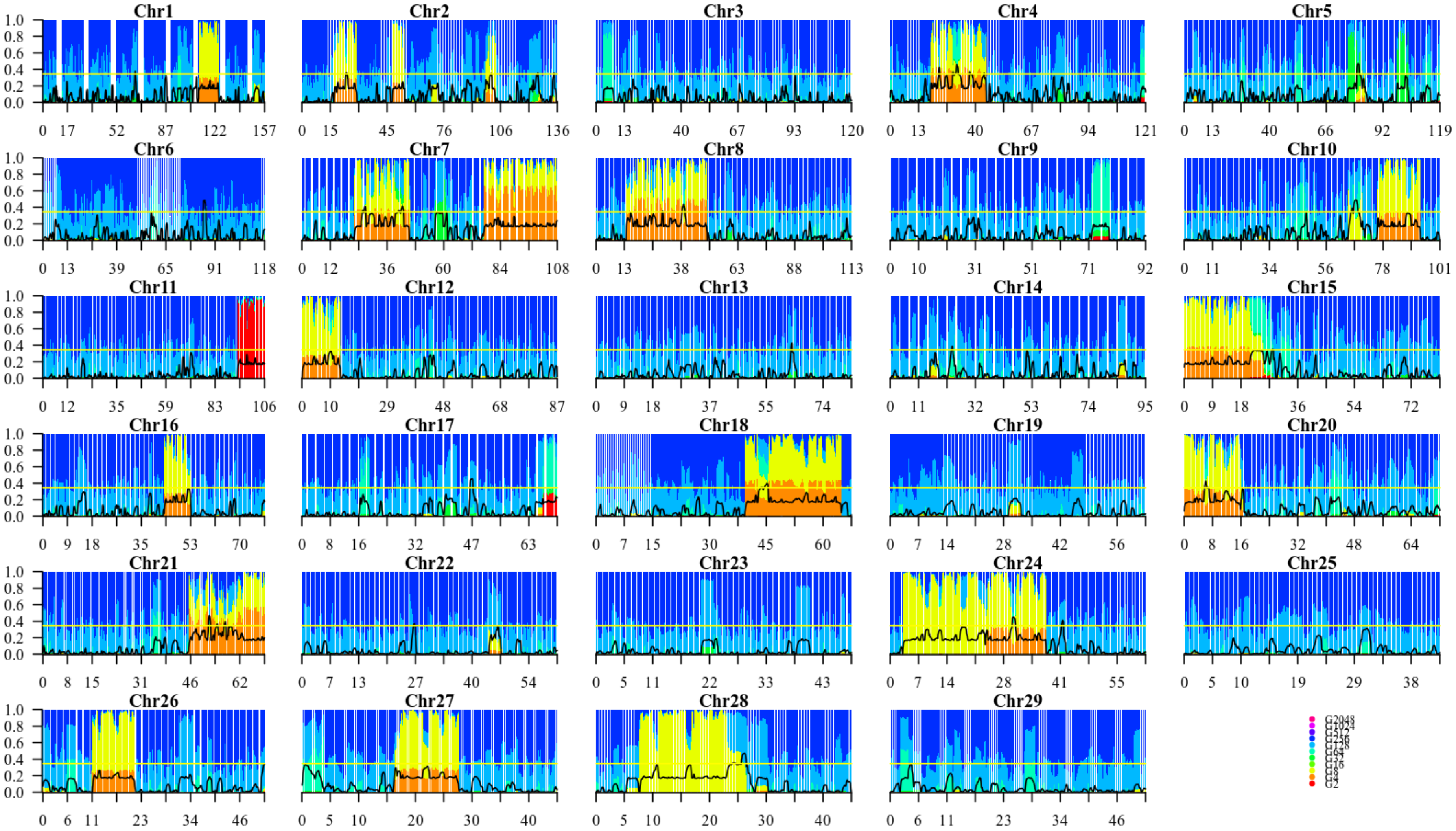

Supplementary Figure 1-18: Local homozygosity-by-descent (HBD) state probabilities for each chromosome for Nsanje. The black line is the mean HBD state probability at each marker. The yellow line is the 99th percentile of marker HBD state probabilities across the genome for the breed.

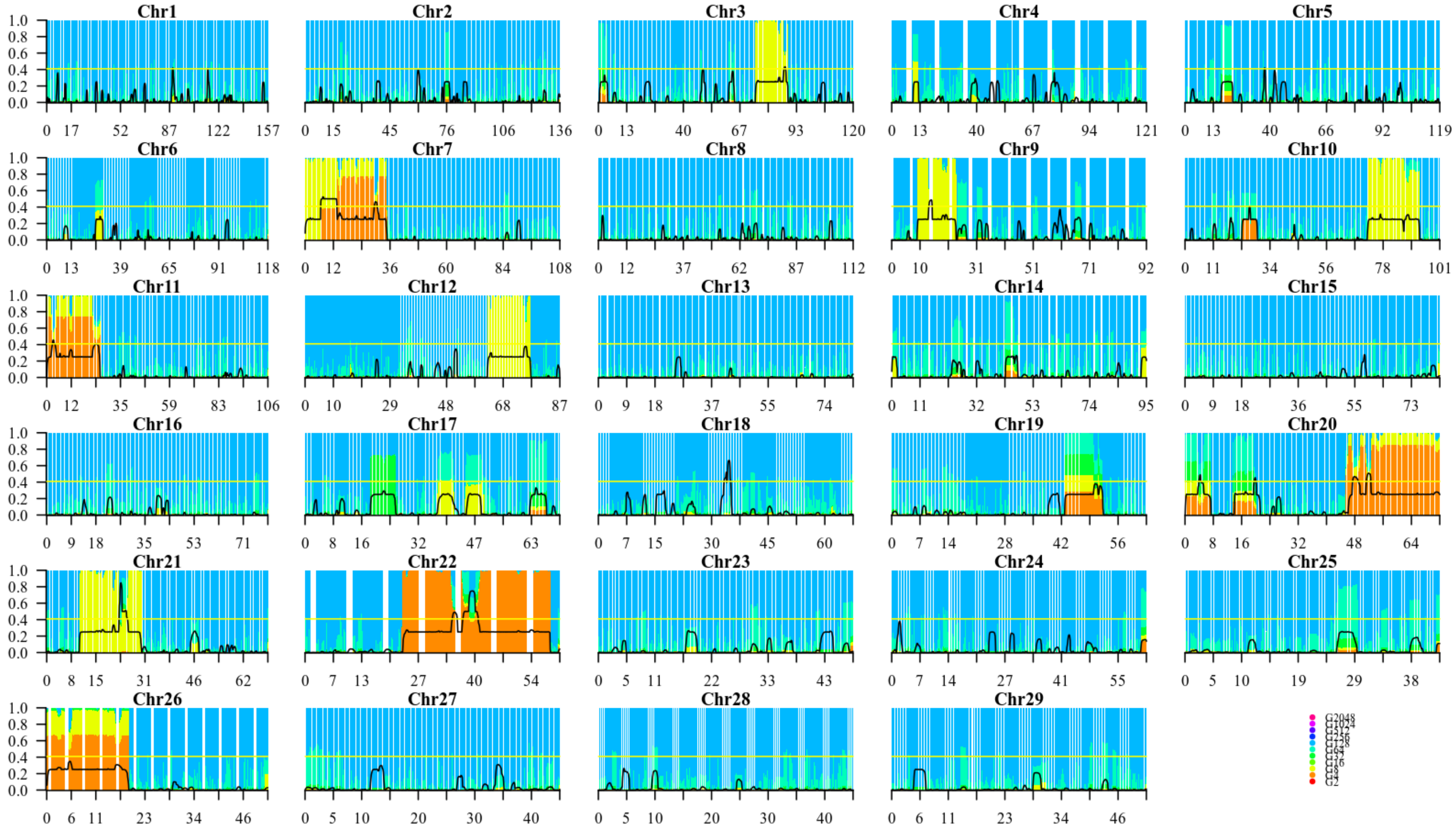

Supplementary Figure 1-19: Local homozygosity-by-descent (HBD) state probabilities for each chromosome for Pafuri. The black line is the mean HBD state probability at each marker. The yellow line is the 99th percentile of marker HBD state probabilities across the genome for the breed.

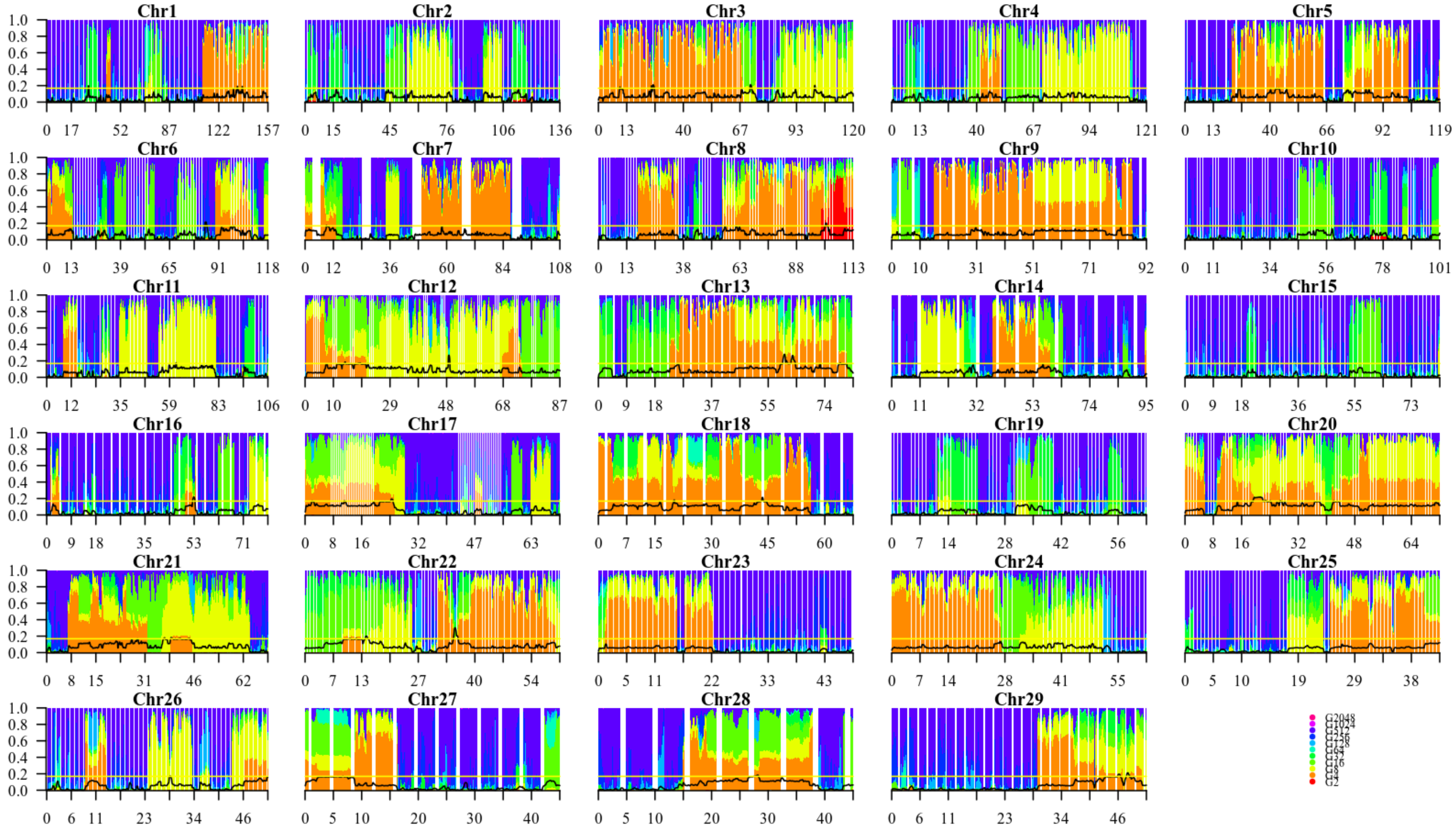

Supplementary Figure 1-20: Local homozygosity-by-descent (HBD) state probabilities for each chromosome for Pare White. The black line is the mean HBD state probability at each marker. The yellow line is the 99th percentile of marker HBD state probabilities across the genome for the breed.

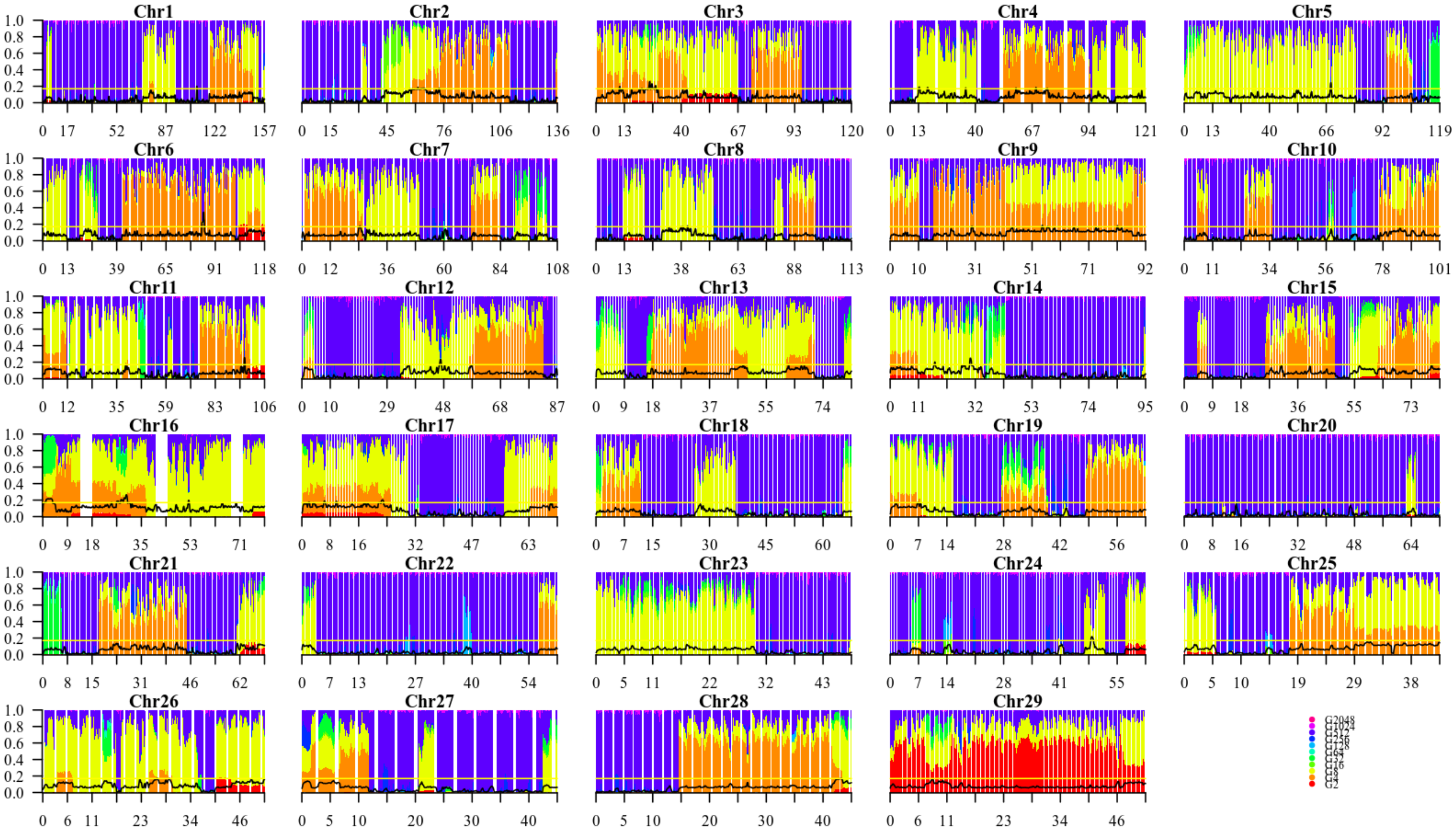

Supplementary Figure 1-21: Local homozygosity-by-descent (HBD) state probabilities for each chromosome for Red Sokoto. The black line is the mean HBD state probability at each marker. The yellow line is the 99th percentile of marker HBD state probabilities across the genome for the breed.

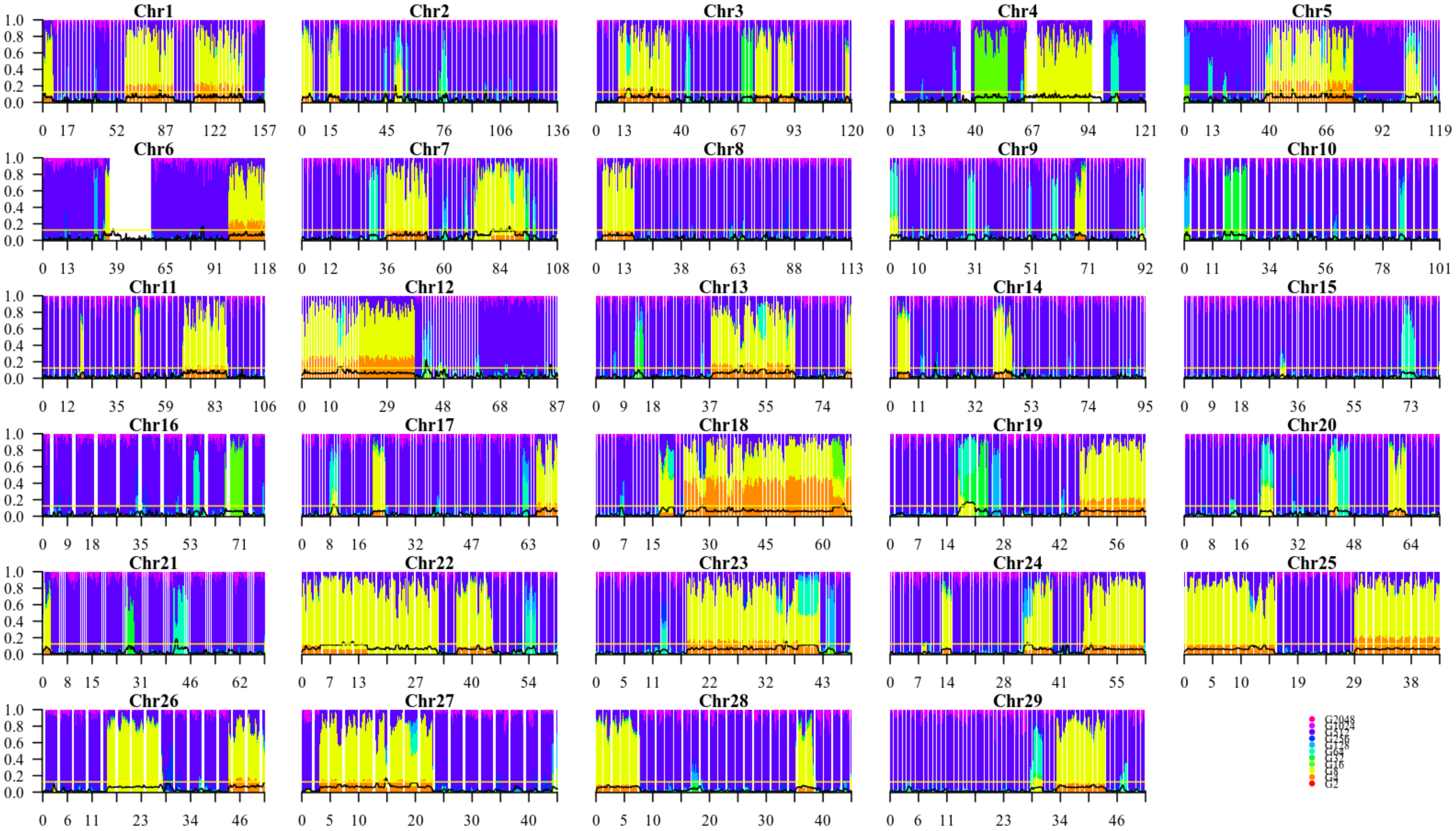

Supplementary Figure 1-22: Local homozygosity-by-descent (HBD) state probabilities for each chromosome for Sahel. The black line is the mean HBD state probability at each marker. The yellow line is the 99th percentile of marker HBD state probabilities across the genome for the breed.

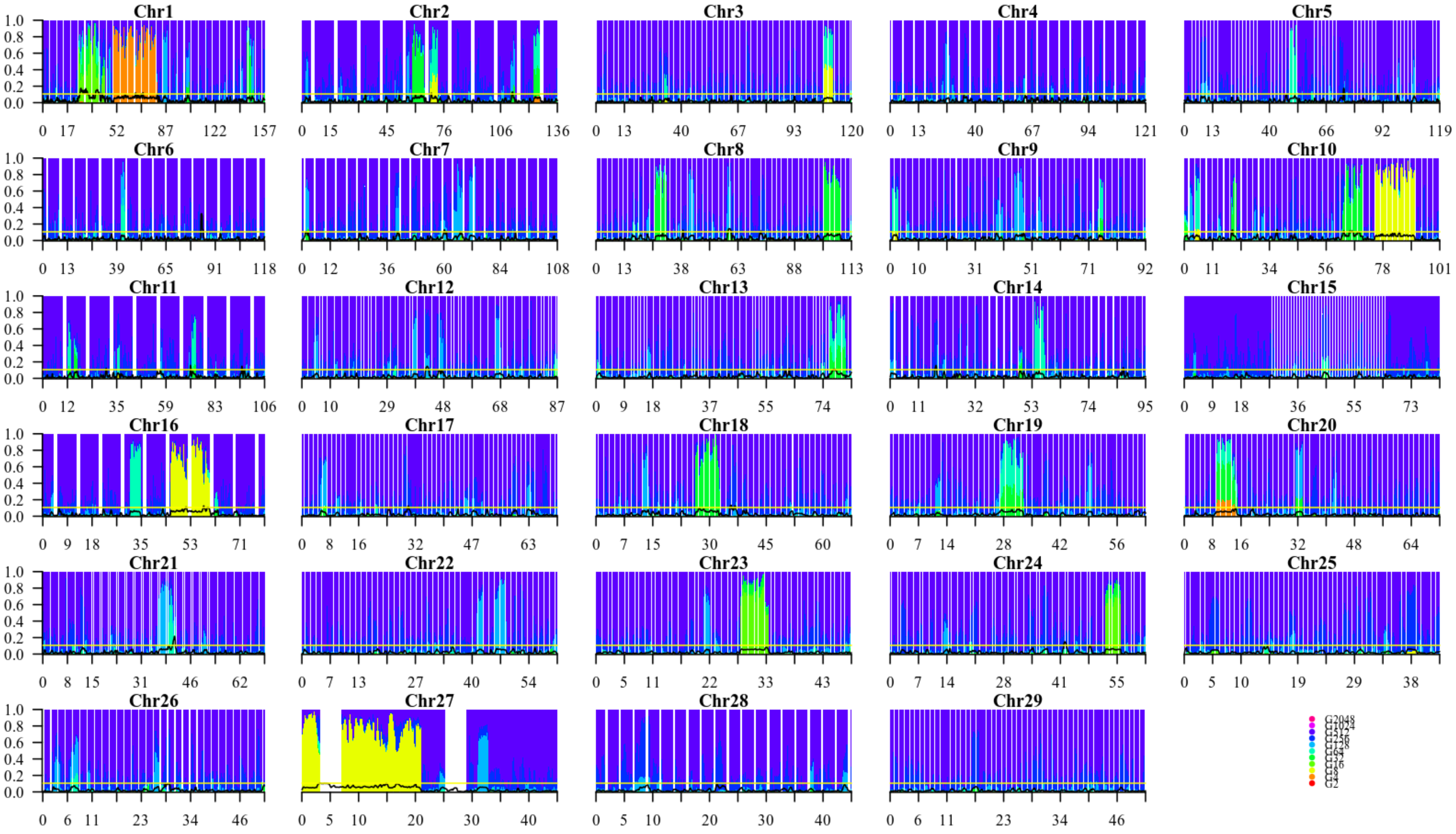

Supplementary Figure 1-23: Local homozygosity-by-descent (HBD) state probabilities for each chromosome for Sebei. The black line is the mean HBD state probability at each marker. The yellow line is the 99th percentile of marker HBD state probabilities across the genome for the breed.

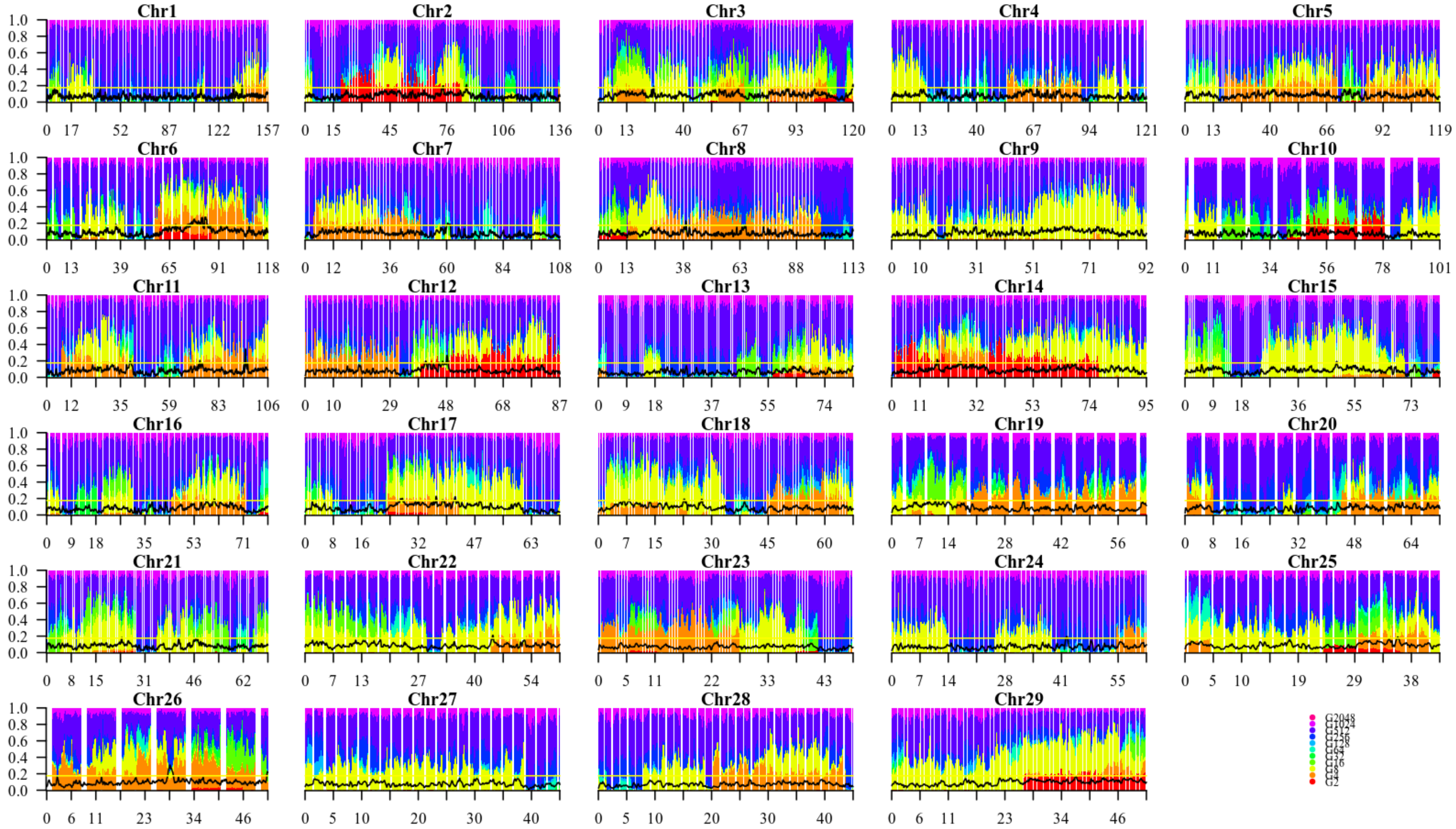

Supplementary Figure 1-24: Local homozygosity-by-descent (HBD) state probabilities for each chromosome for Small East African. The black line is the mean HBD state probability at each marker. The yellow line is the 99th percentile of marker HBD state probabilities across the genome for the breed.

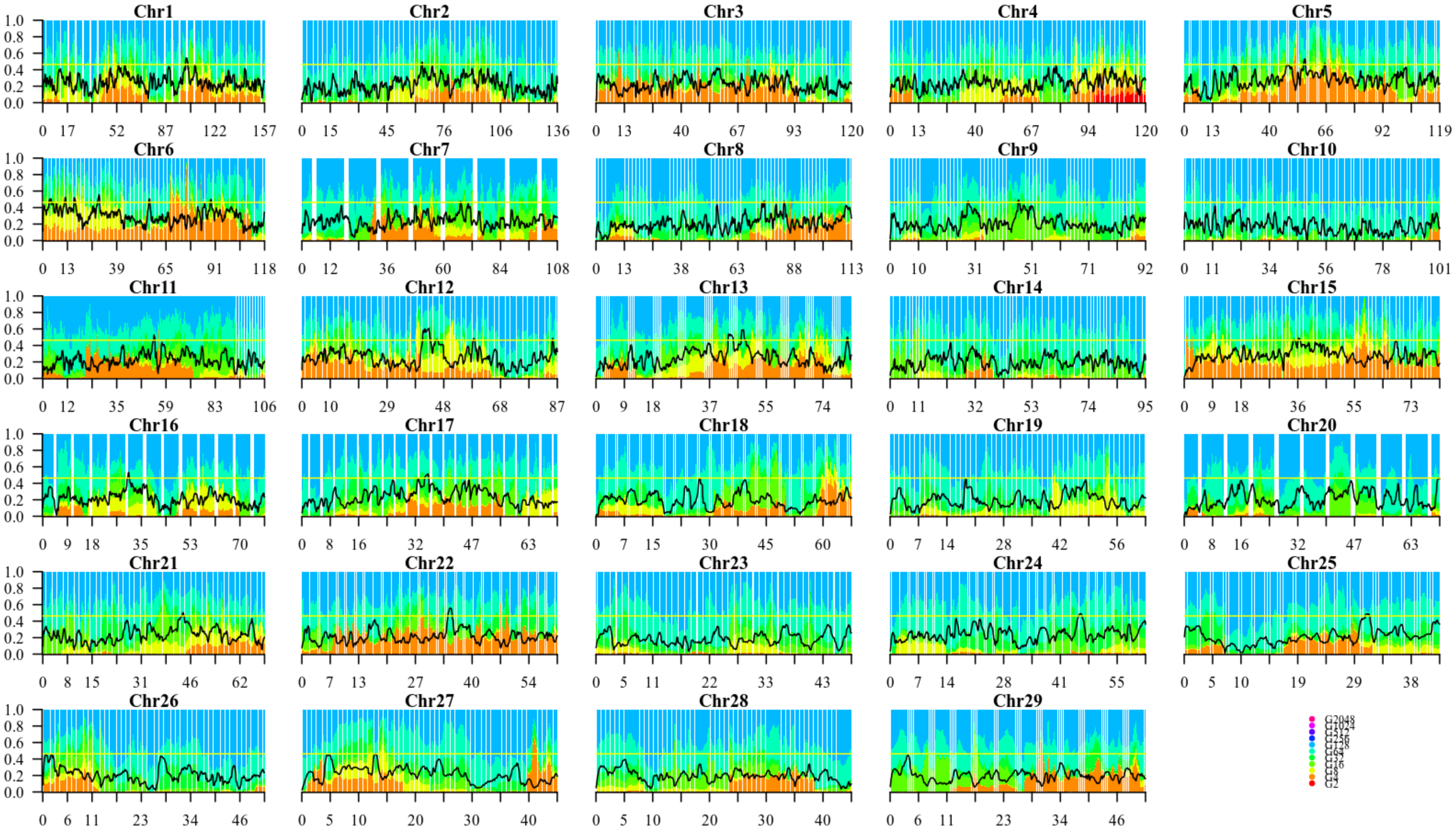

Supplementary Figure 1-25: Local homozygosity-by-descent (HBD) state probabilities for each chromosome for Sofia. The black line is the mean HBD state probability at each marker. The yellow line is the 99th percentile of marker HBD state probabilities across the genome for the breed.

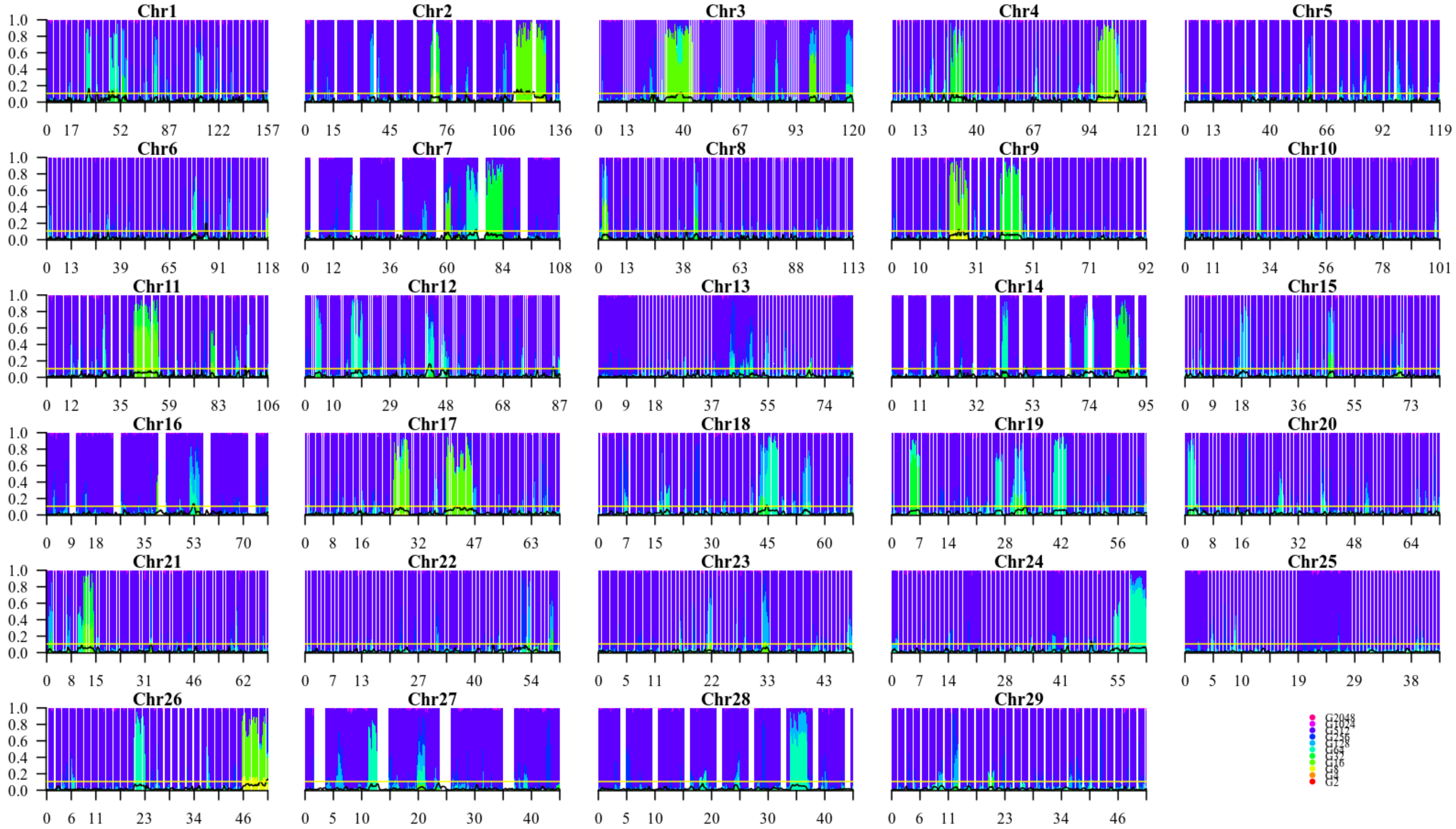

Supplementary Figure 1-26: Local homozygosity-by-descent (HBD) state probabilities for each chromosome for Sonjo. The black line is the mean HBD state probability at each marker. The yellow line is the 99th percentile of marker HBD state probabilities across the genome for the breed.

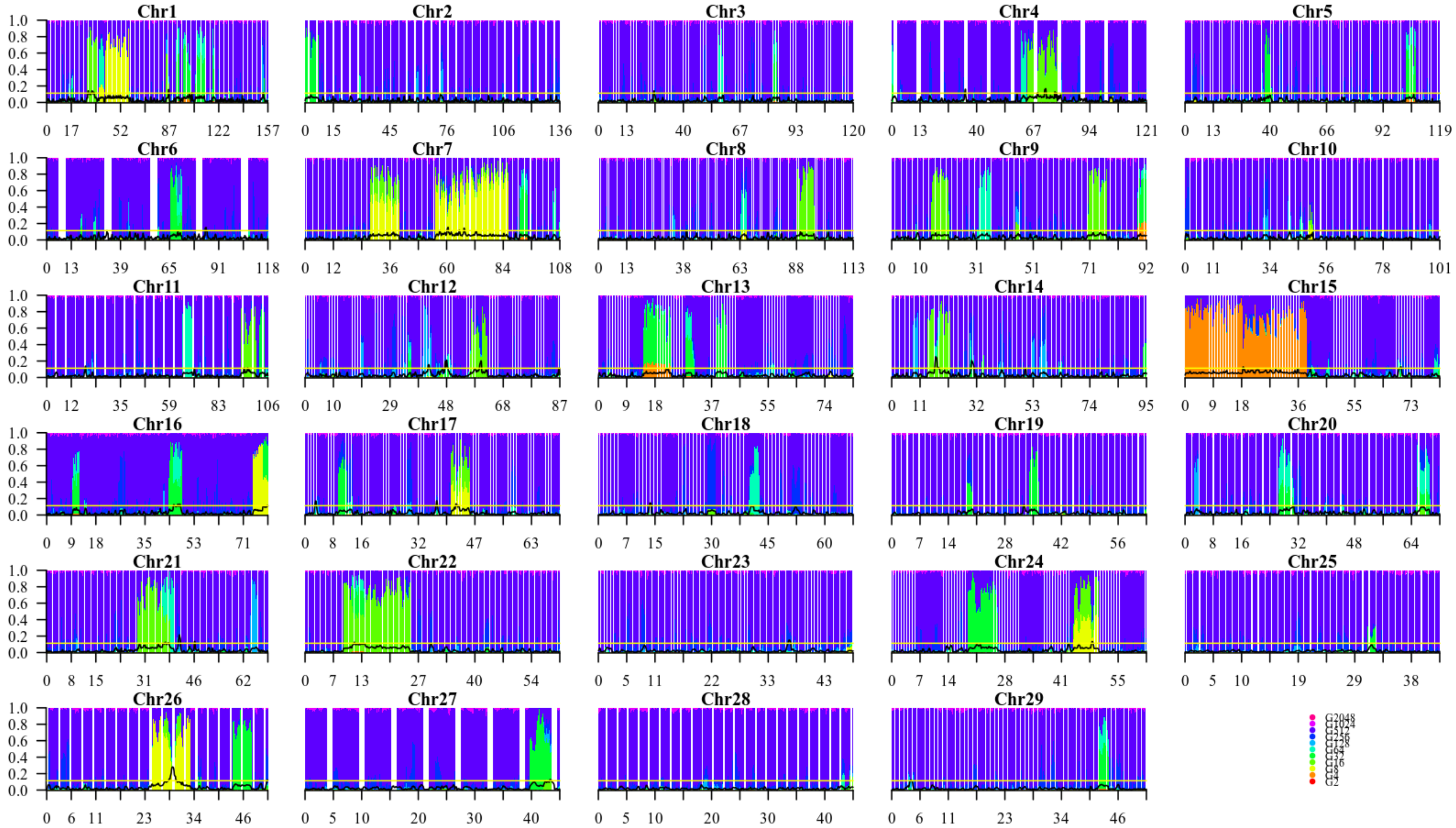

Supplementary Figure 1-27: Local homozygosity-by-descent (HBD) state probabilities for each chromosome for Soudanaise. The black line is the mean HBD state probability at each marker. The yellow line is the 99th percentile of marker HBD state probabilities across the genome for the breed.

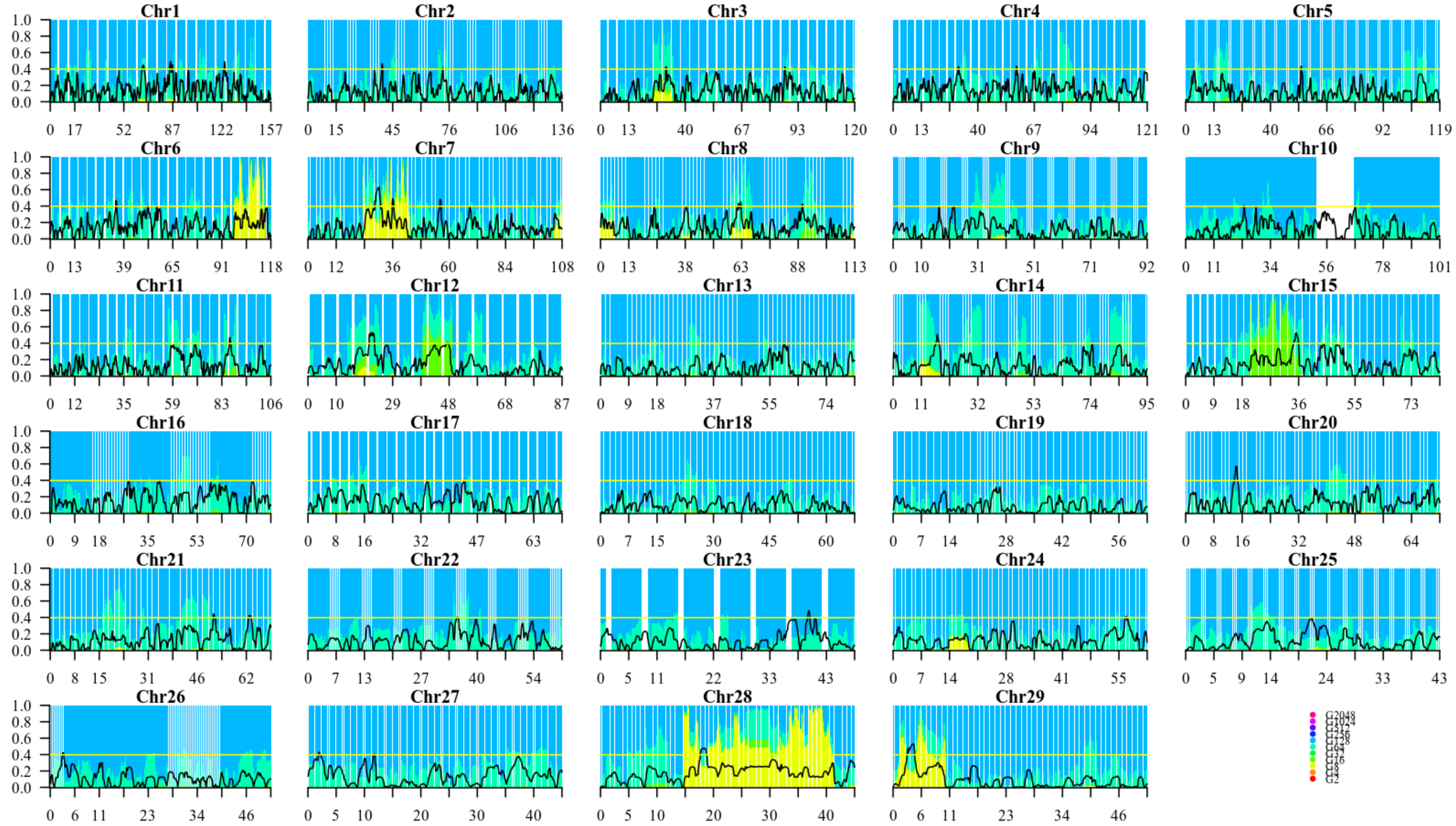

Supplementary Figure 1-28: Local homozygosity-by-descent (HBD) state probabilities for each chromosome for SudOuest. The black line is the mean HBD state probability at each marker. The yellow line is the 99th percentile of marker HBD state probabilities across the genome for the breed.

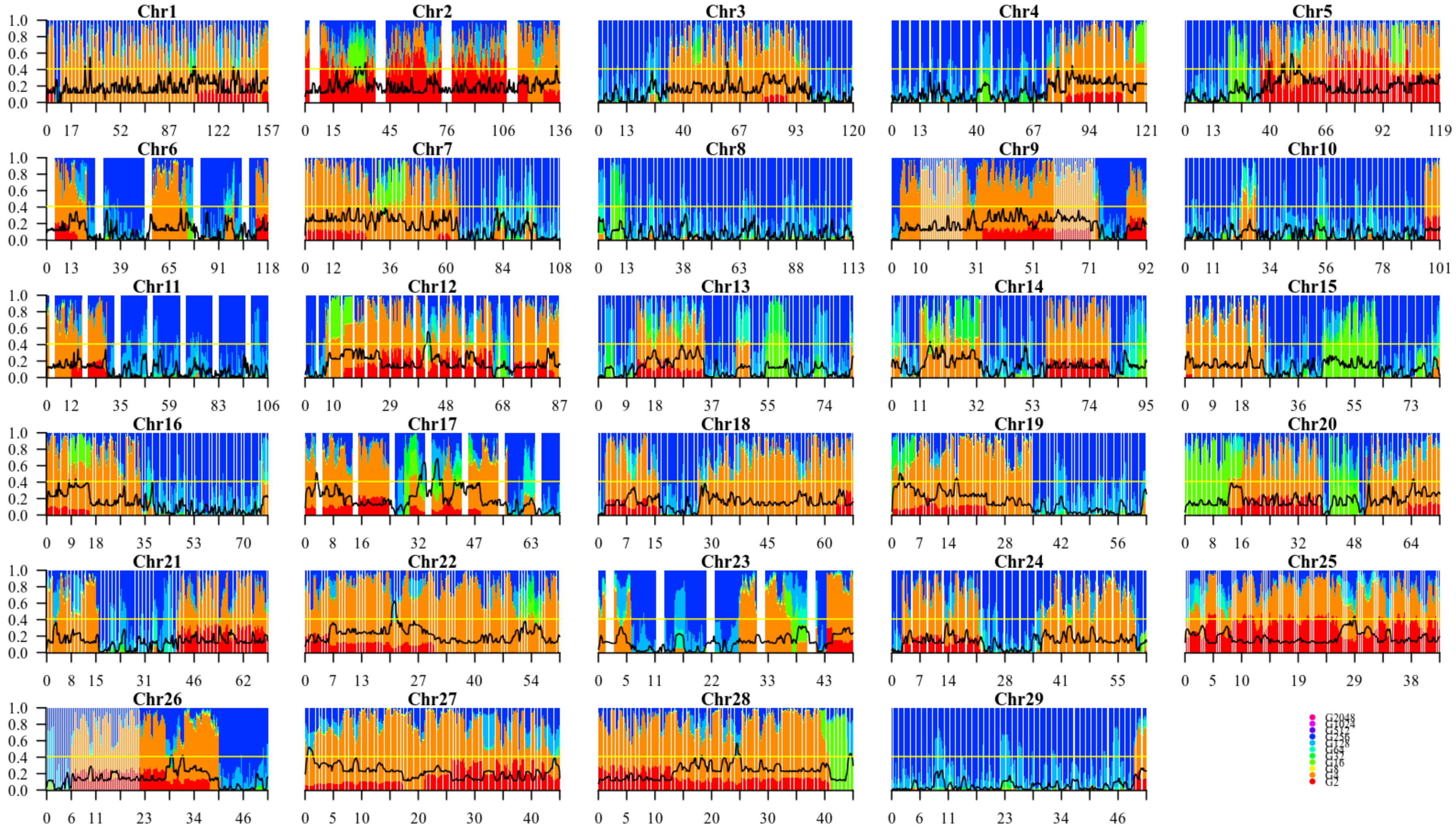

Supplementary Figure 1-29: Local homozygosity-by-descent (HBD) state probabilities for each chromosome for Thyolo. The black line is the mean HBD state probability at each marker. The yellow line is the 99th percentile of marker HBD state probabilities across the genome for the breed.

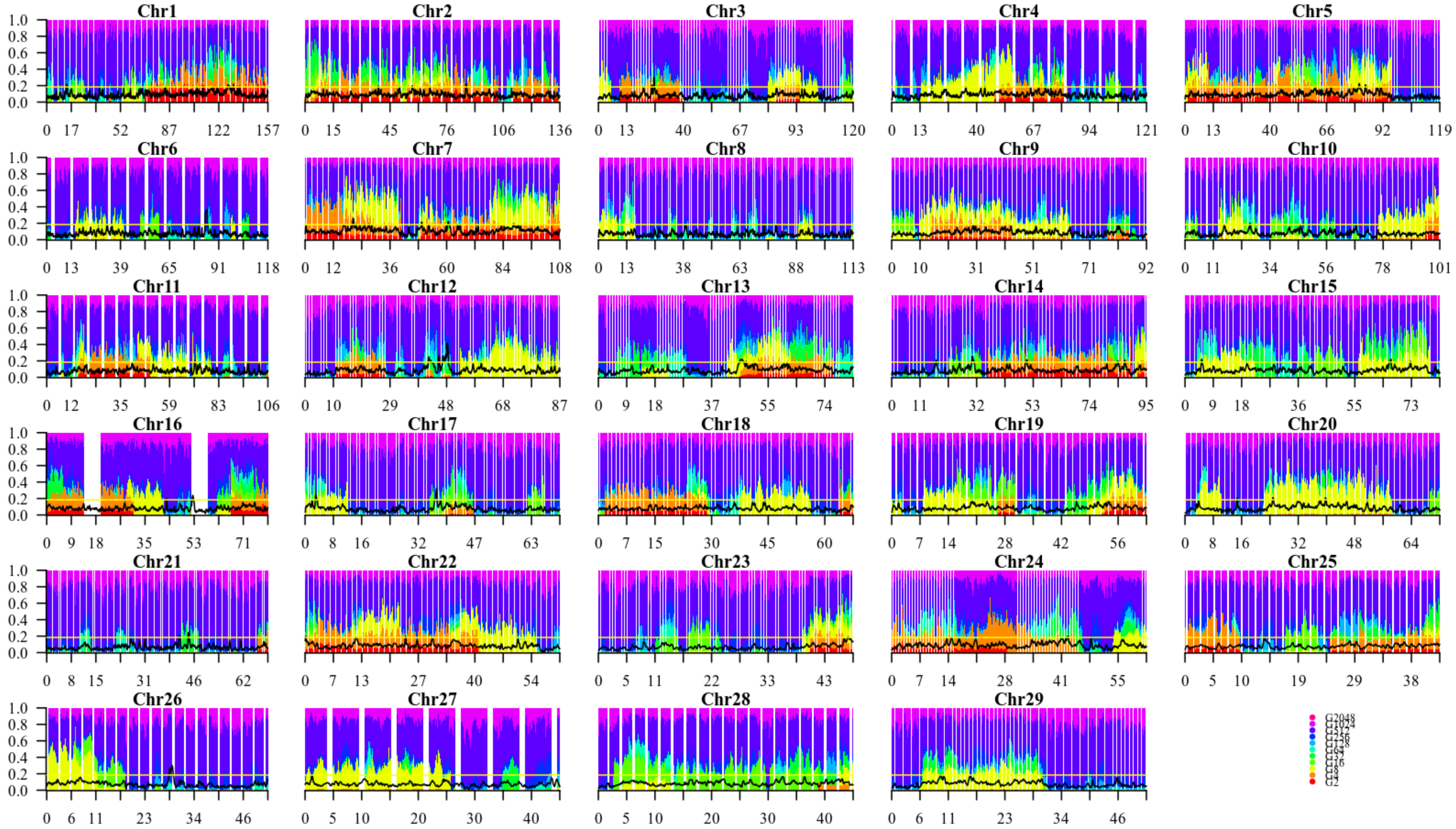

Supplementary Figure 1-30: Local homozygosity-by-descent (HBD) state probabilities for each chromosome for West African Dwarf. The black line is the mean HBD state probability at each marker. The yellow line is the 99th percentile of marker HBD state probabilities across the genome for the breed.

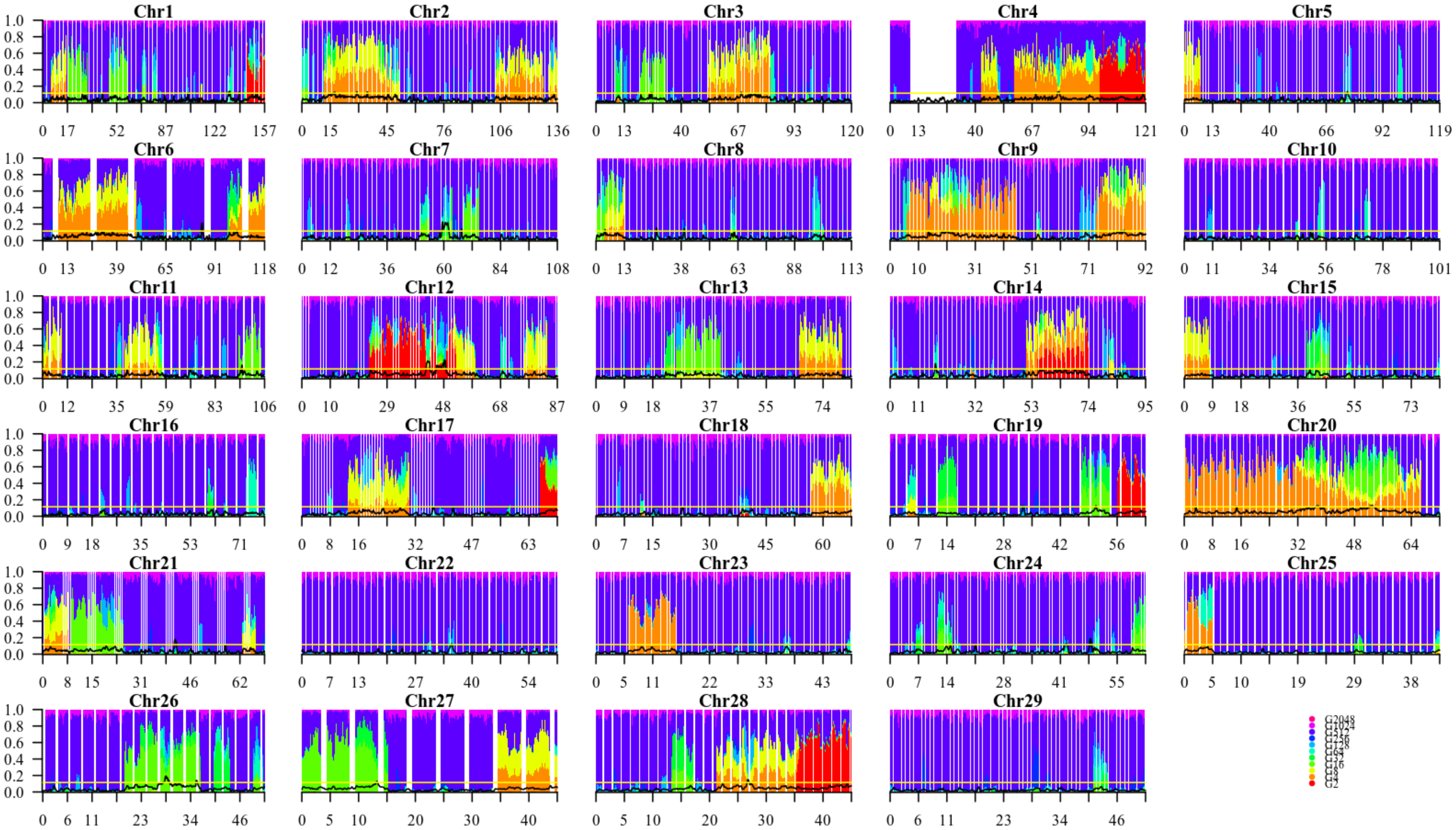

Supplementary Figure 1-31: Local homozygosity-by-descent (HBD) state probabilities for each chromosome for Woyitoguji. The black line is the mean HBD state probability at each marker. The yellow line is the 99th percentile of marker HBD state probabilities across the genome for the breed.

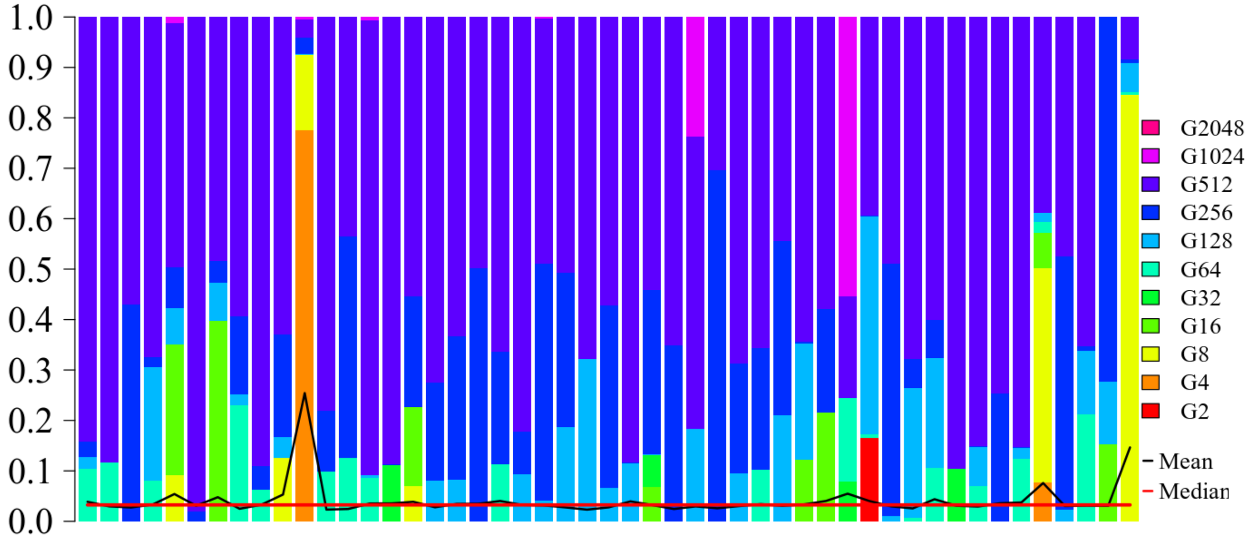

Supplementary Figure 2-1: Global inbreeding levels for each animal for Abergelle

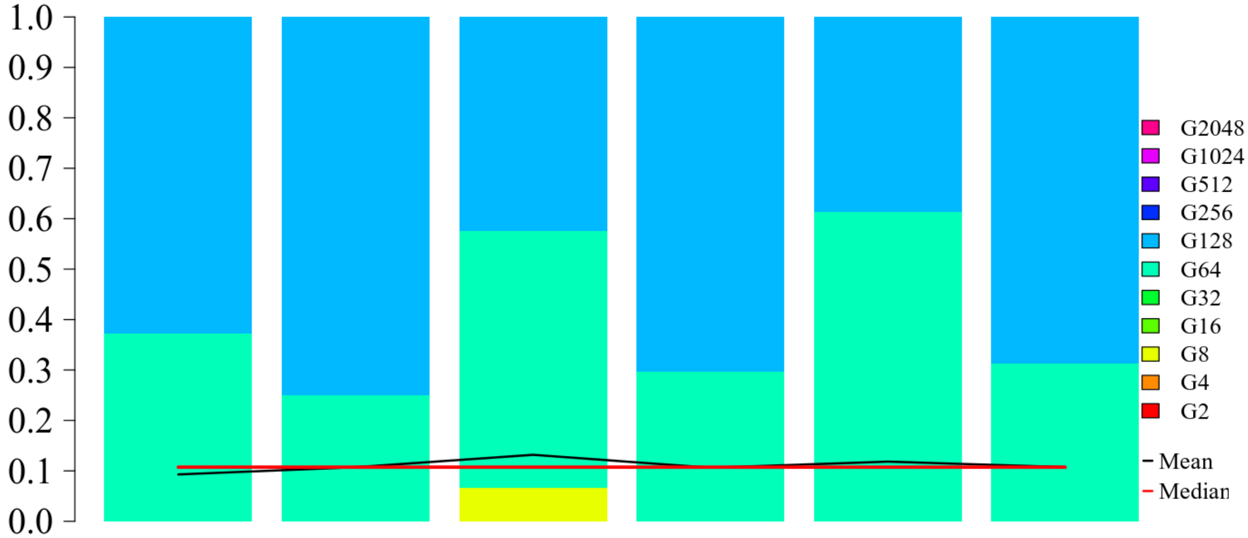

Supplementary Figure 2-2: Global inbreeding levels for each animal for Androy

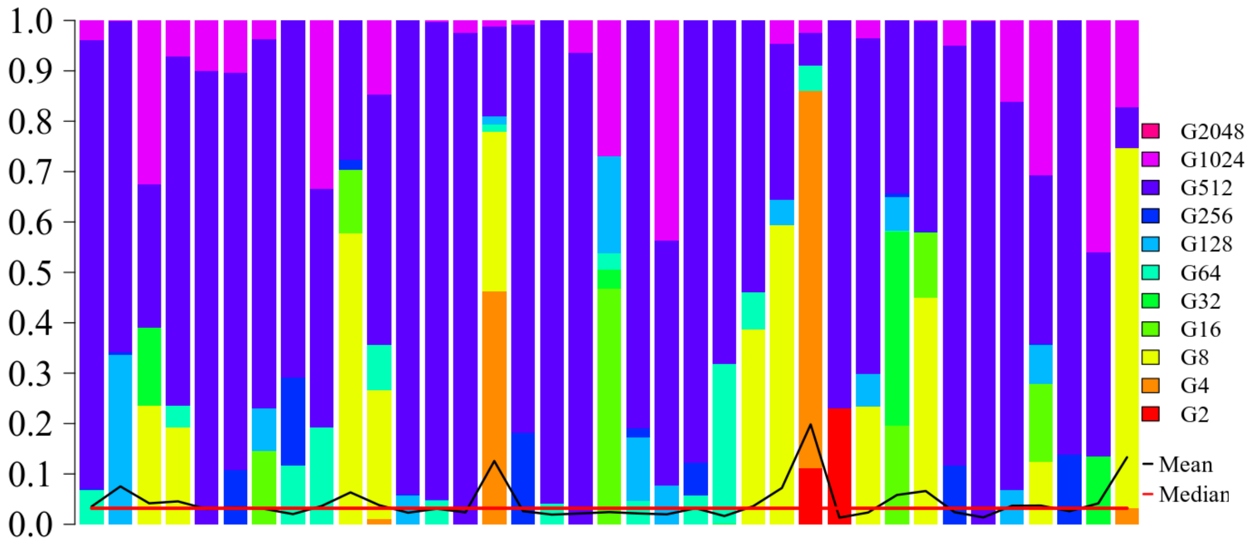

Supplementary Figure 2-3: Global inbreeding levels for each animal for Cameroon

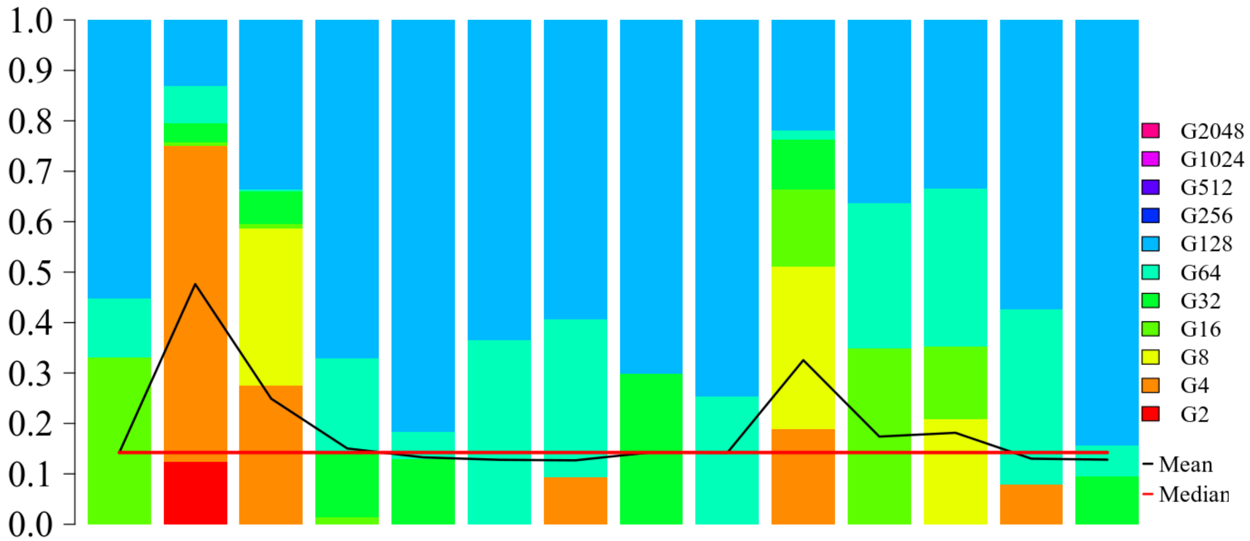

Supplementary Figure 2-4: Global inbreeding levels for each animal for Diana

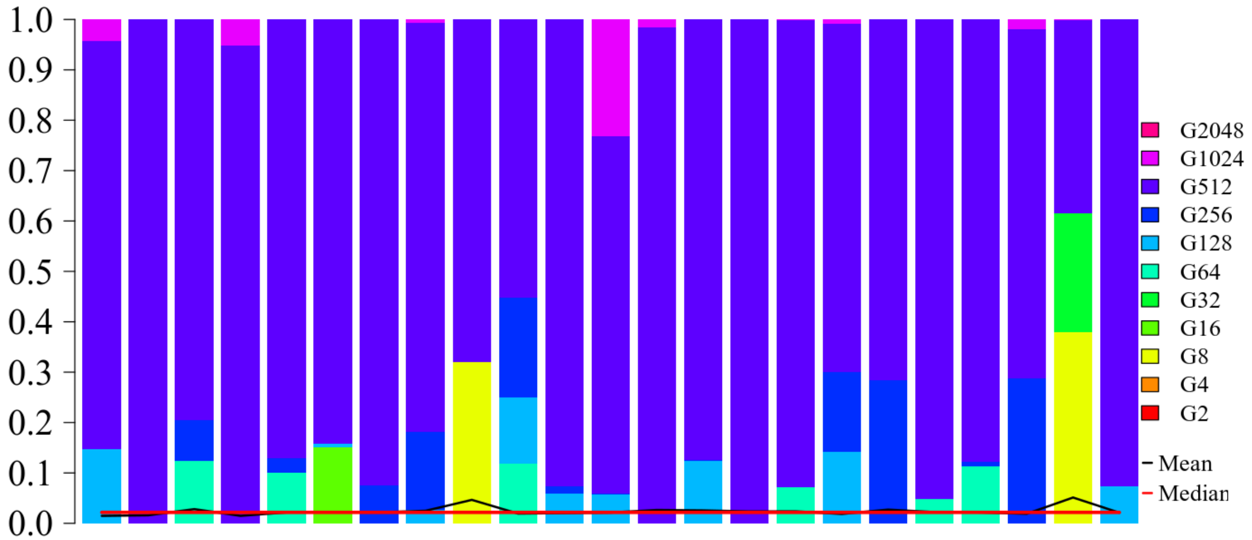

Supplementary Figure 2-5: Global inbreeding levels for each animal for Galla

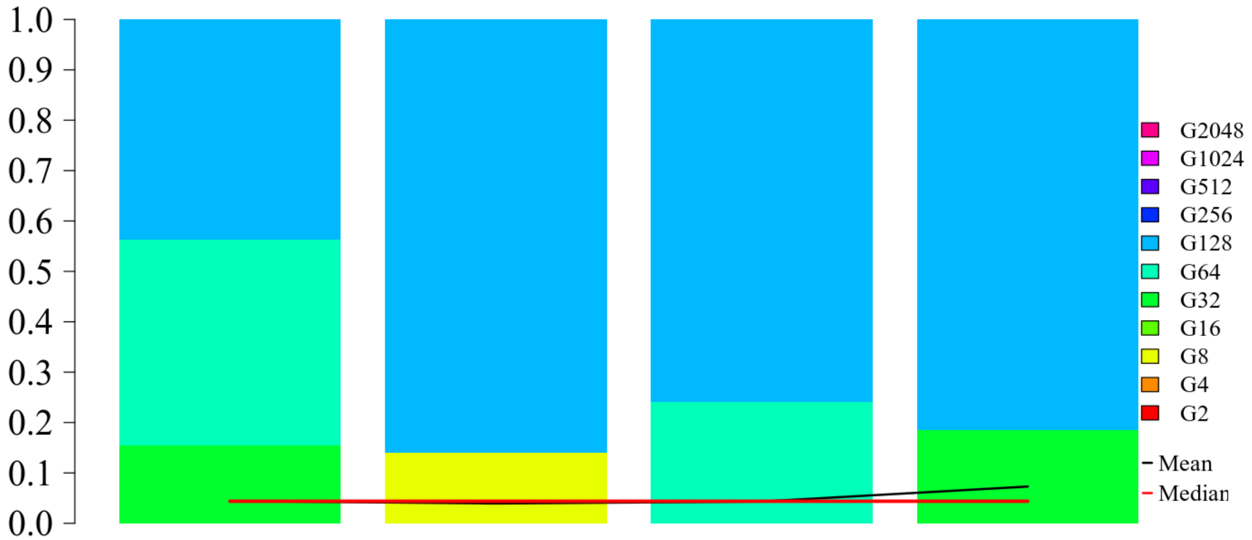

Supplementary Figure 2-6: Global inbreeding levels for each animal for Gaza

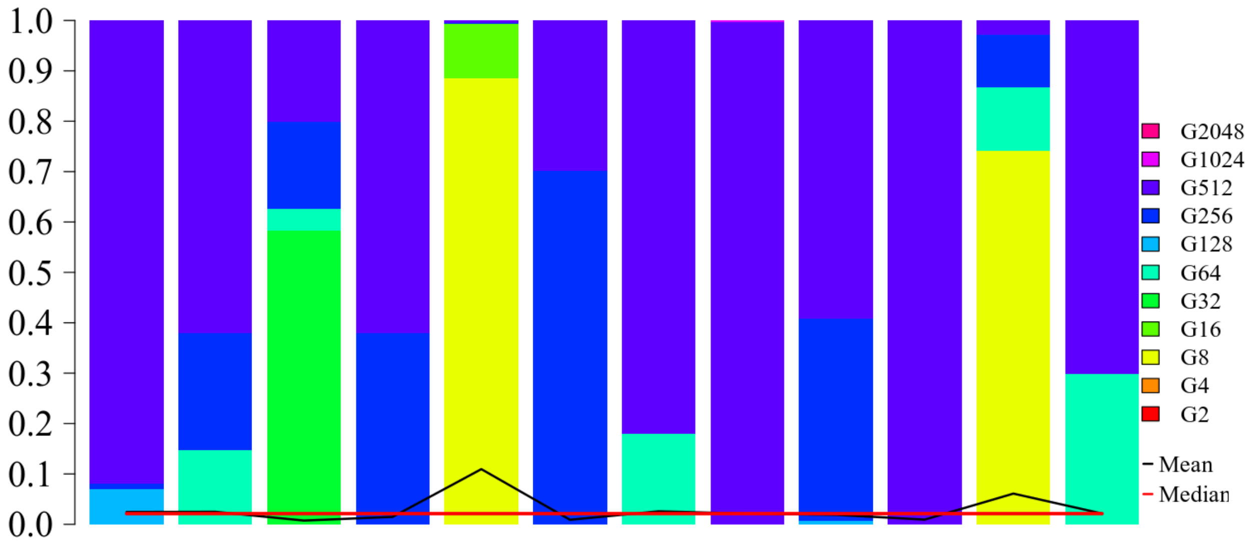

Supplementary Figure 2-7: Global inbreeding levels for each animal for Gogo

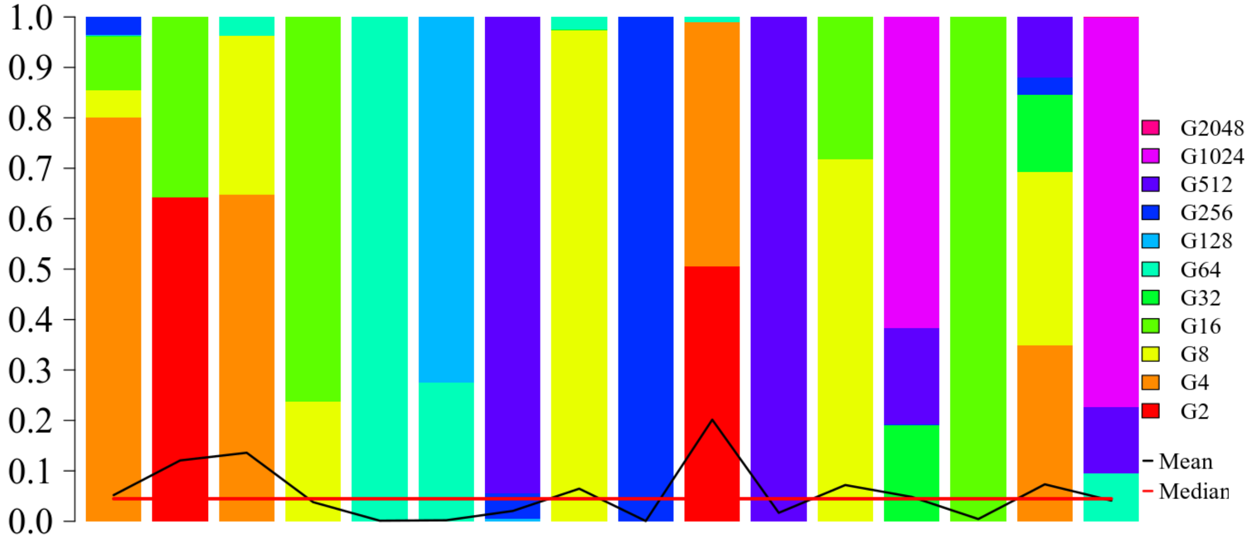

Supplementary Figure 2-8: Global inbreeding levels for each animal for Guera

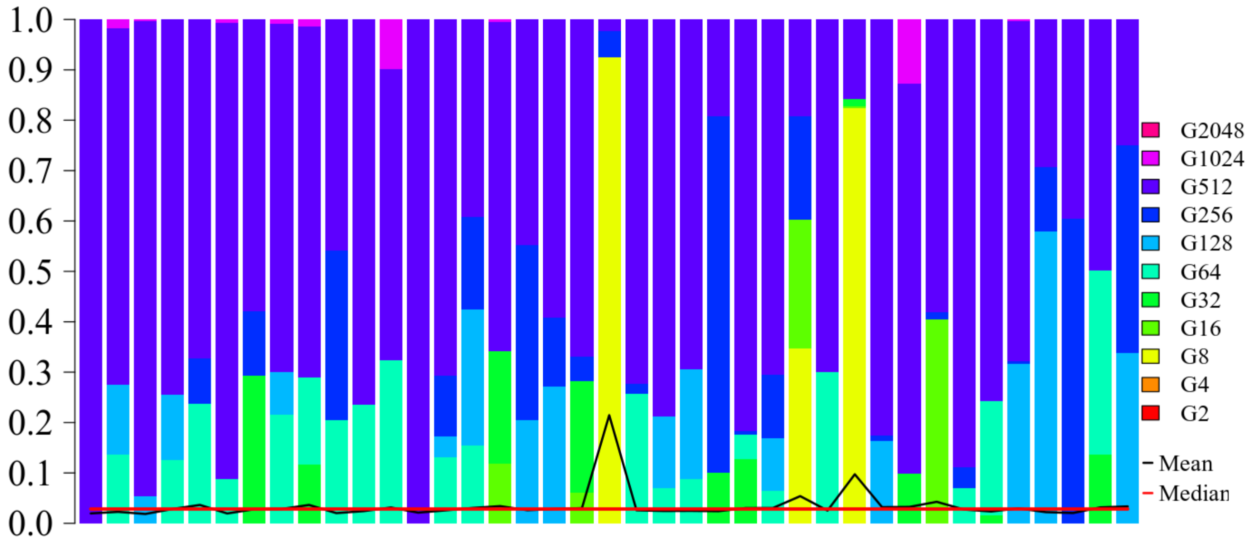

Supplementary Figure 2-9: Global inbreeding levels for each animal for Gumez

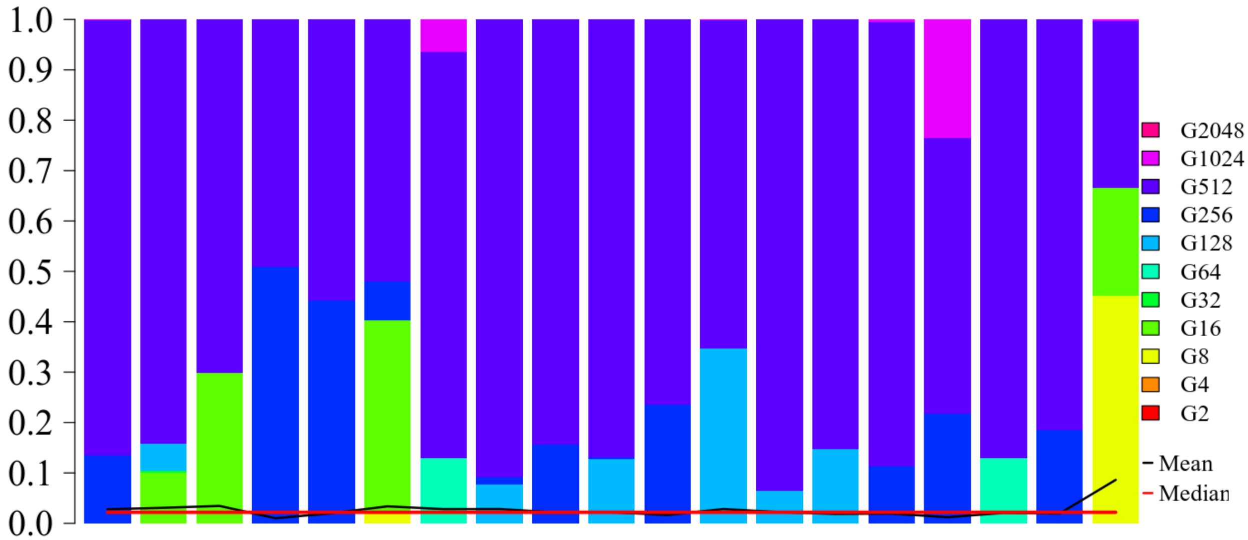

Supplementary Figure 2-10: Global inbreeding levels for each animal for Karamoja

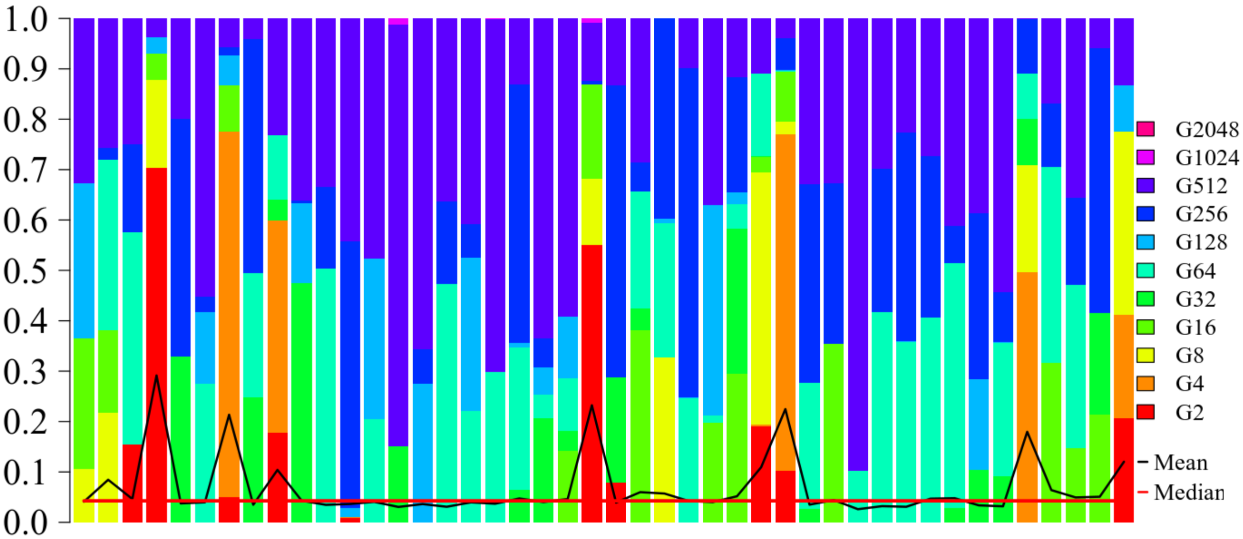

Supplementary Figure 2-11: Global inbreeding levels for each animal for Keffa

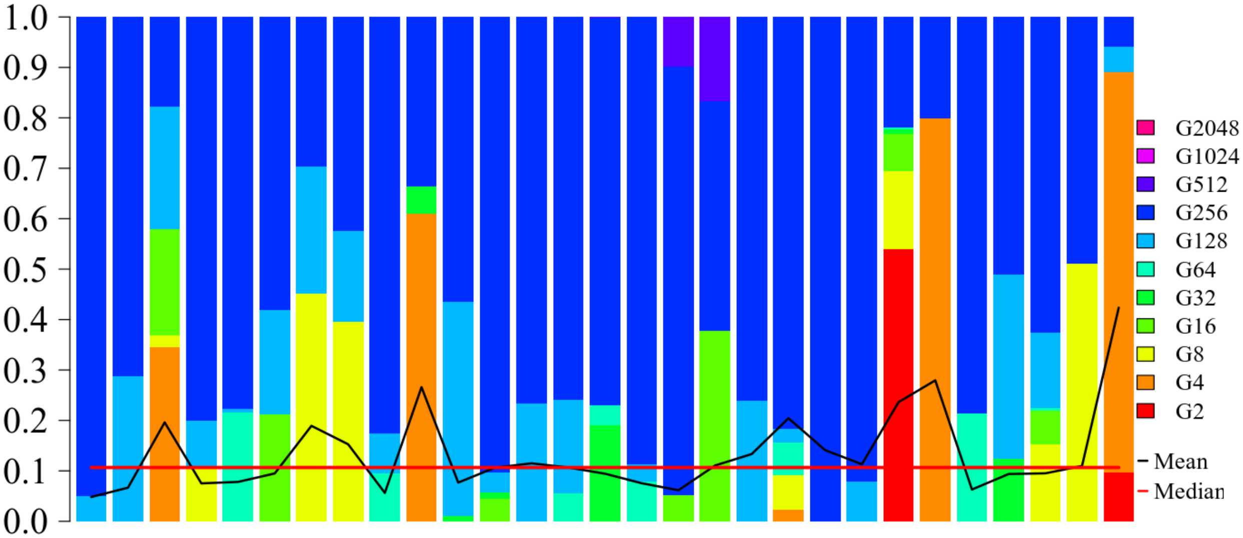

Supplementary Figure 2-12: Global inbreeding levels for each animal for Landin

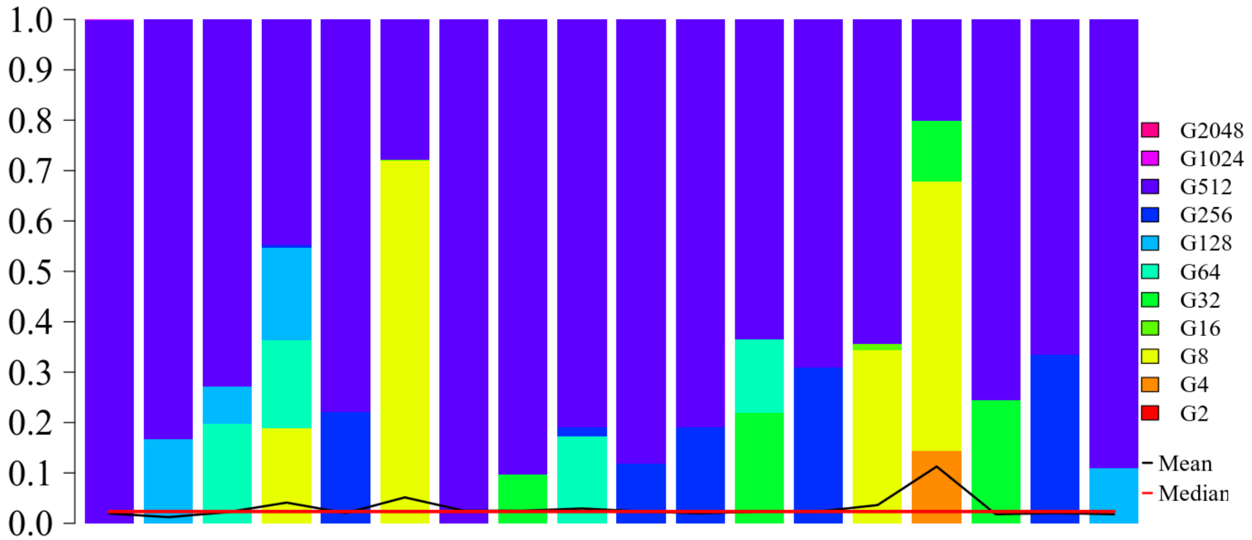

Supplementary Figure 2-13: Global inbreeding levels for each animal for Maasai

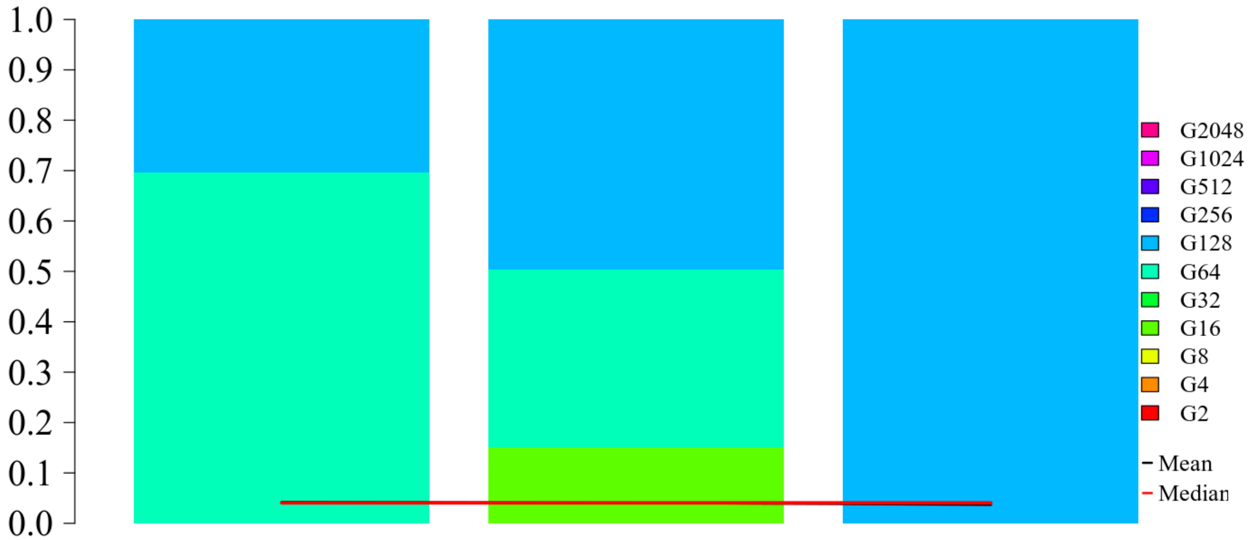

Supplementary Figure 2-14: Global inbreeding levels for each animal for Manica

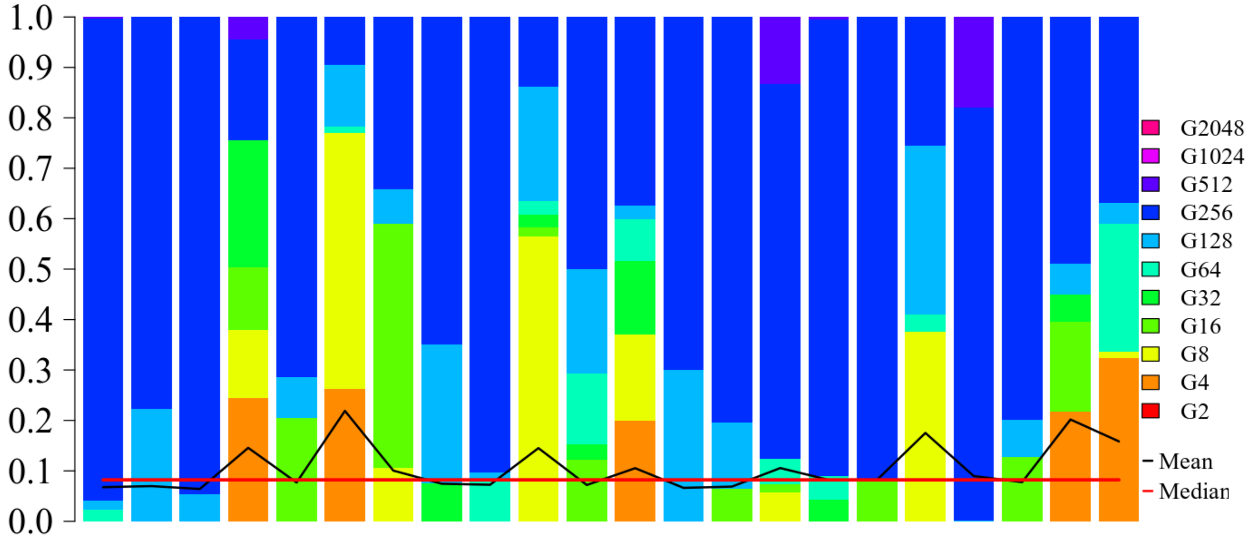

Supplementary Figure 2-16: Global inbreeding levels for each animal for Mashona

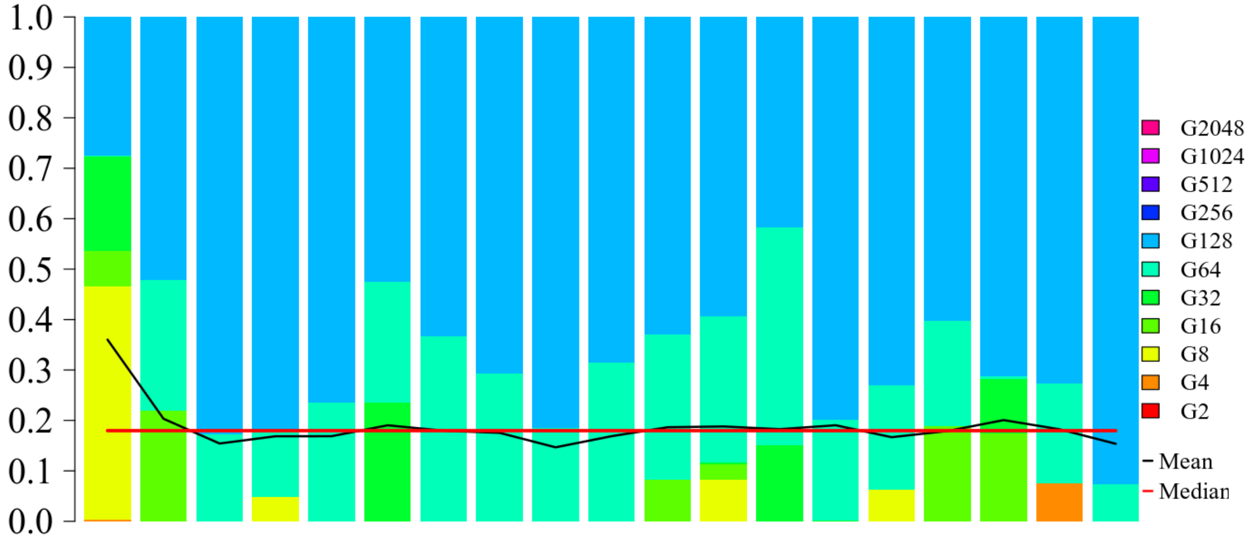

Supplementary Figure 2-15: Global inbreeding levels for each animal for Menabe

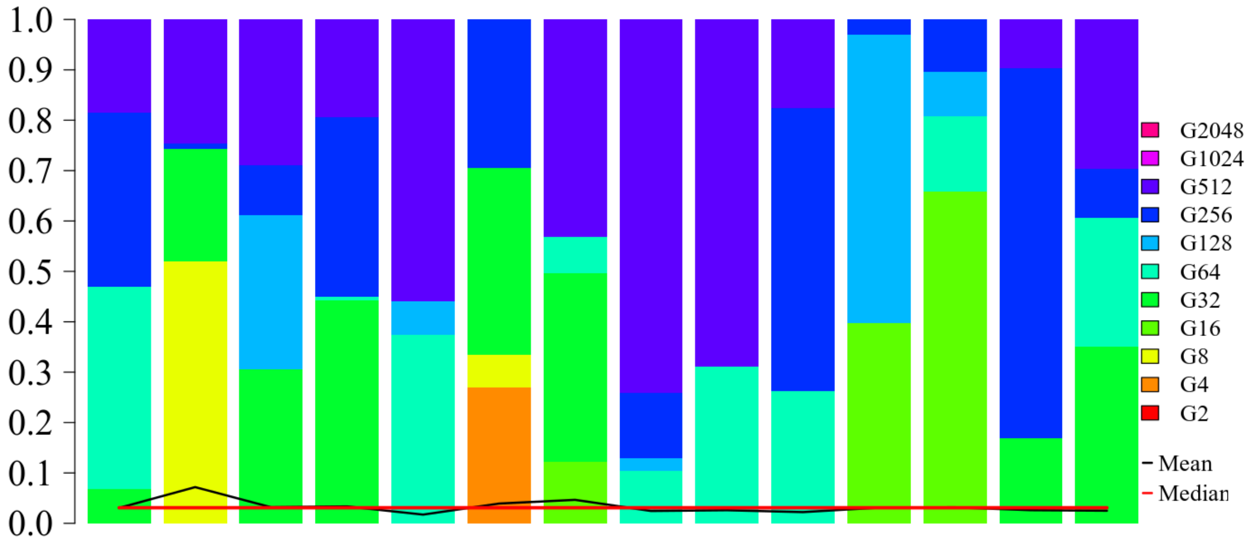

Supplementary Figure 2-17: Global inbreeding levels for each animal for Naine

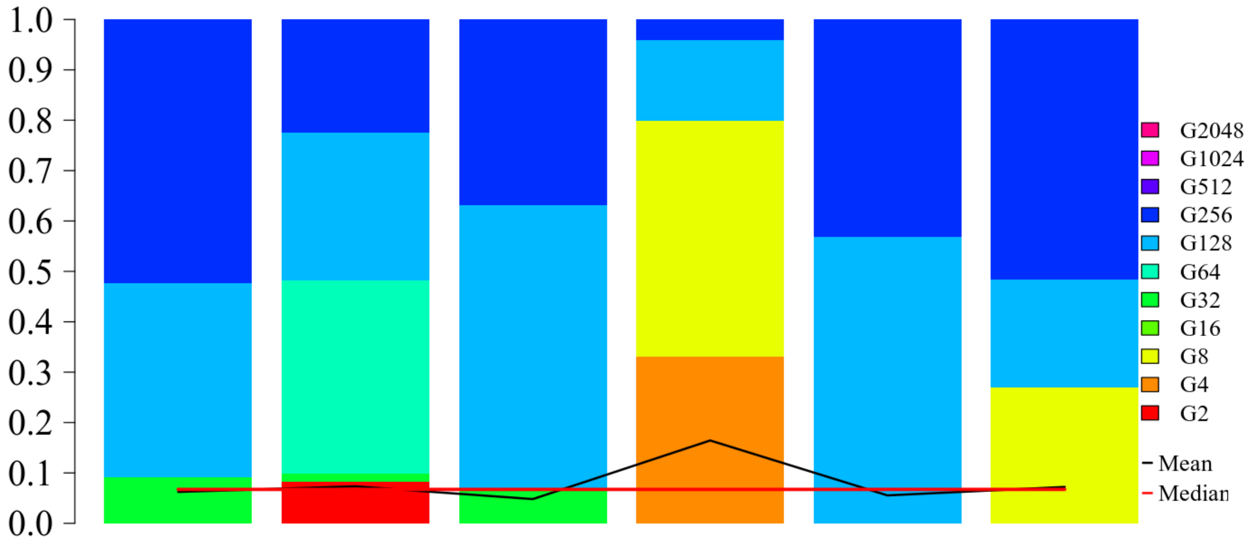

Supplementary Figure 2-18: Global inbreeding levels for each animal for Nsanje

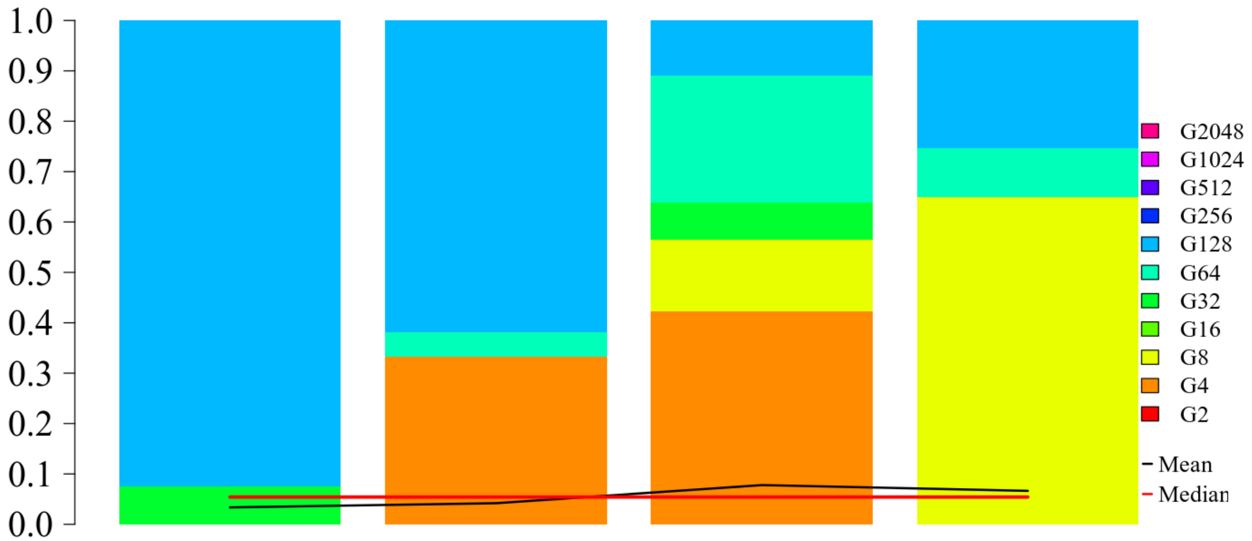

Supplementary Figure 2-19: Global inbreeding levels for each animal for Pafuri

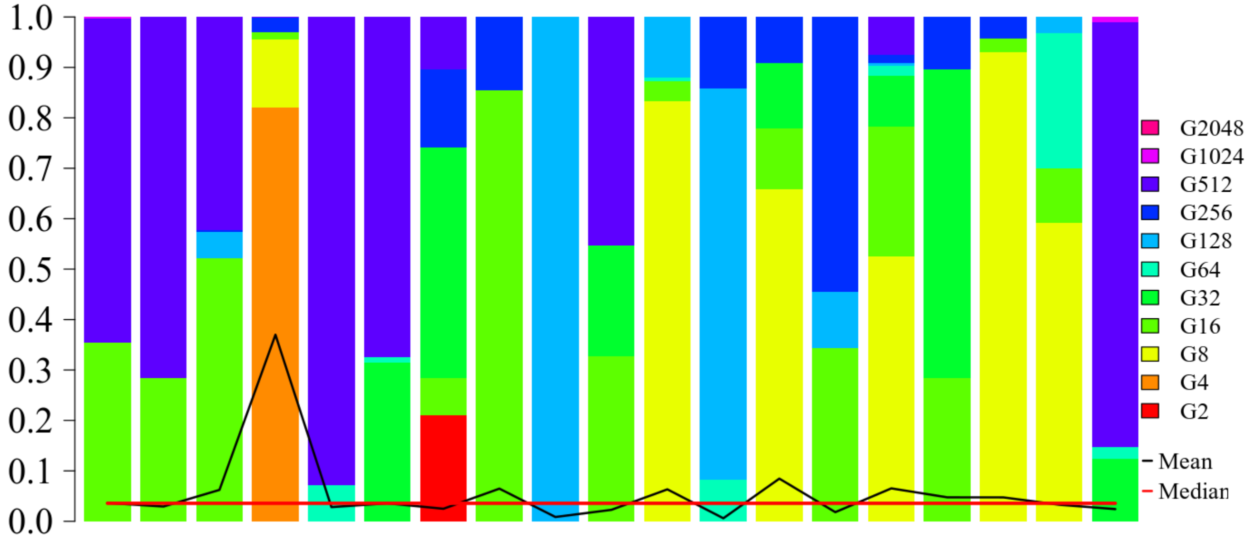

Supplementary Figure 2-20: Global inbreeding levels for each animal for Pare White

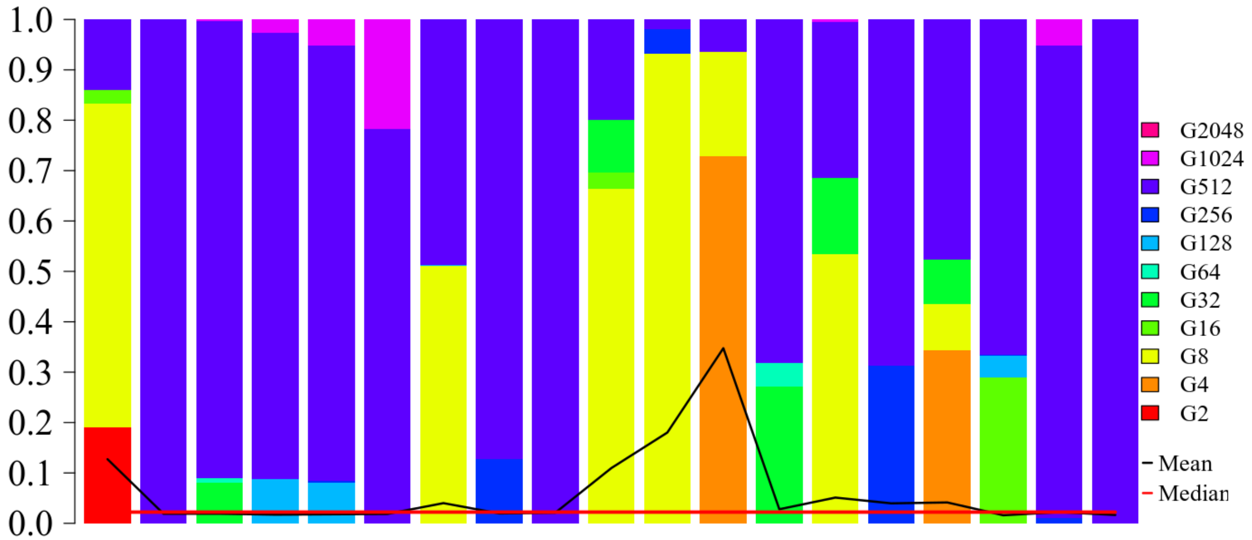

Supplementary Figure 2-21: Global inbreeding levels for each animal for Red Sokoto

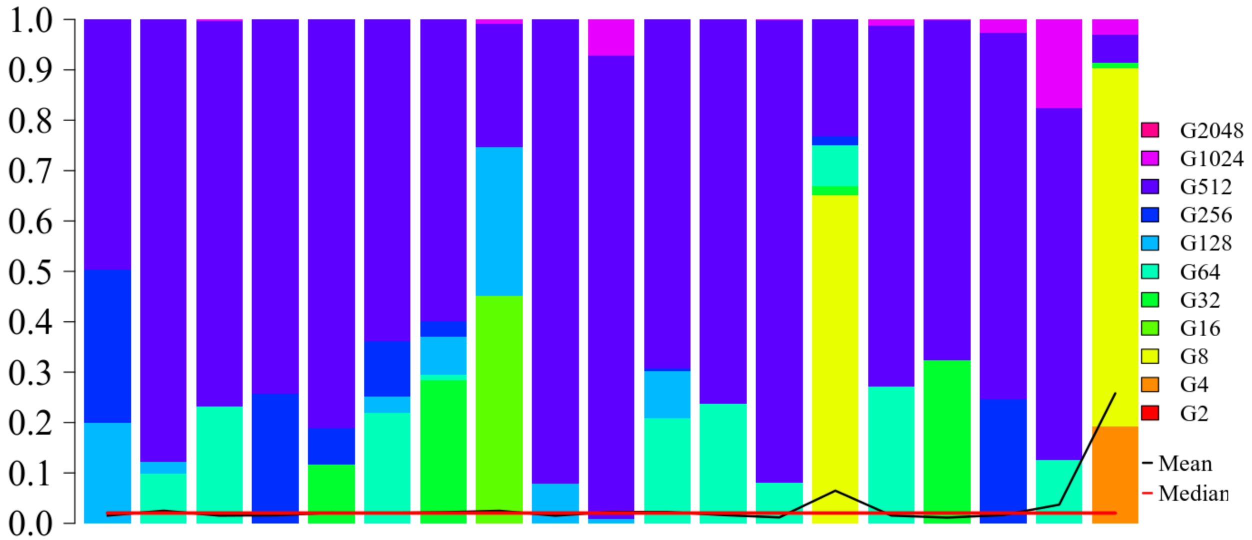

Supplementary Figure 2-25: Global inbreeding levels for each animal for Sahel

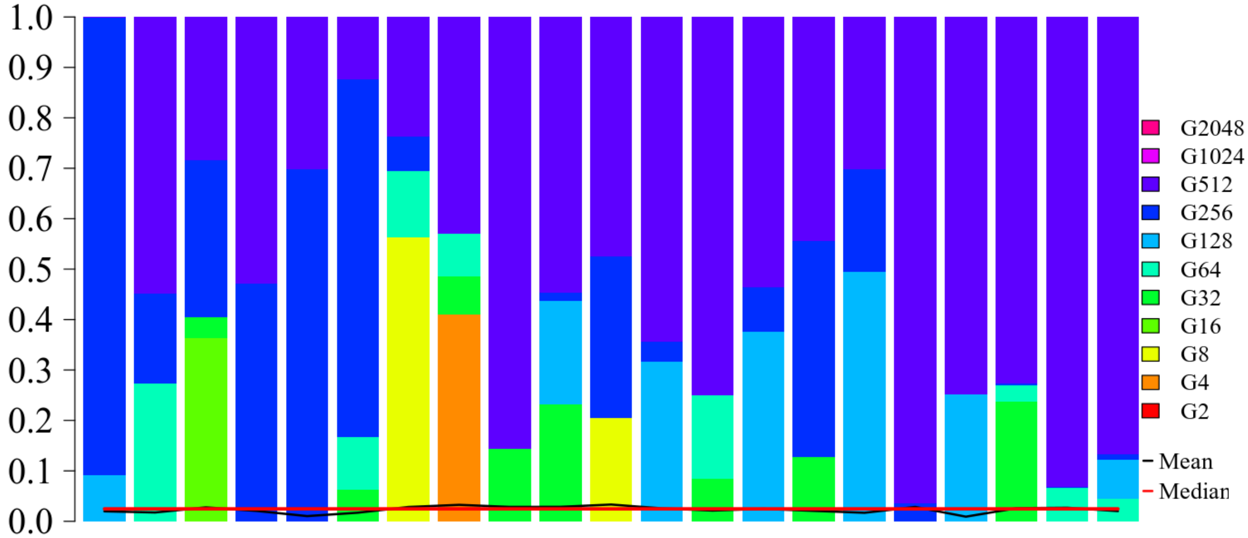

Supplementary Figure 2-24: Global inbreeding levels for each animal for Sebei

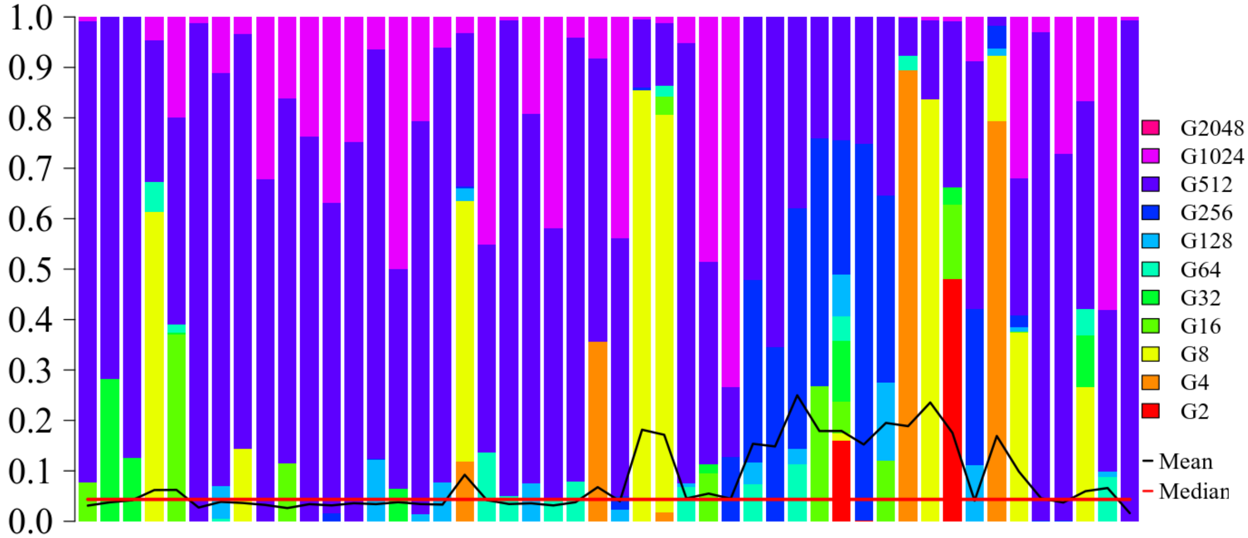

Supplementary Figure 2-23: Global inbreeding levels for each animal for Small East African

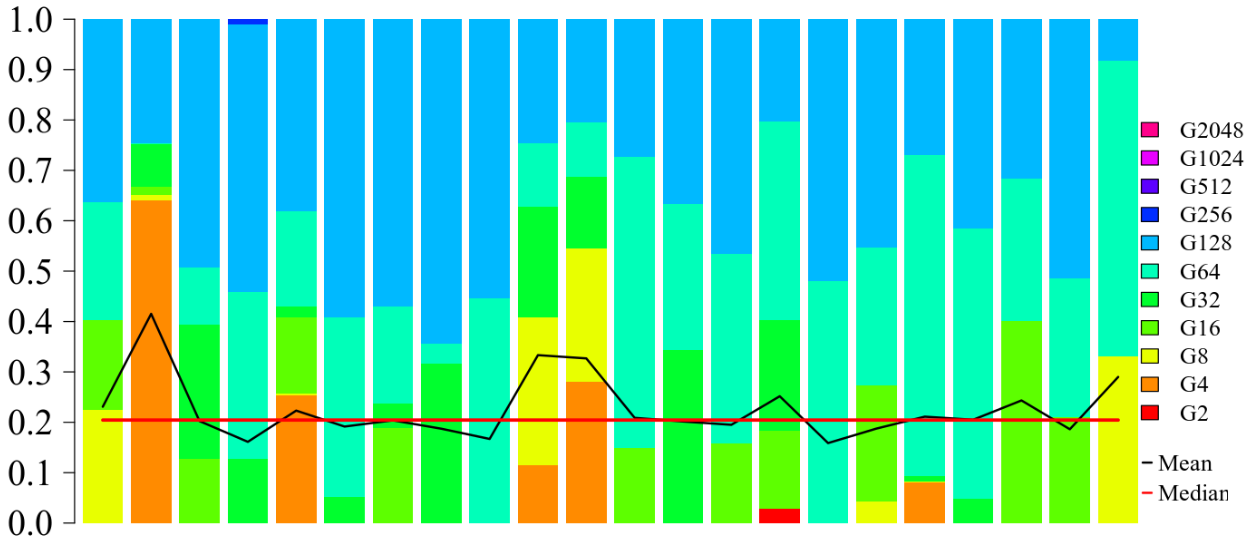

Supplementary Figure 2-27: Global inbreeding levels for each animal for Sofia

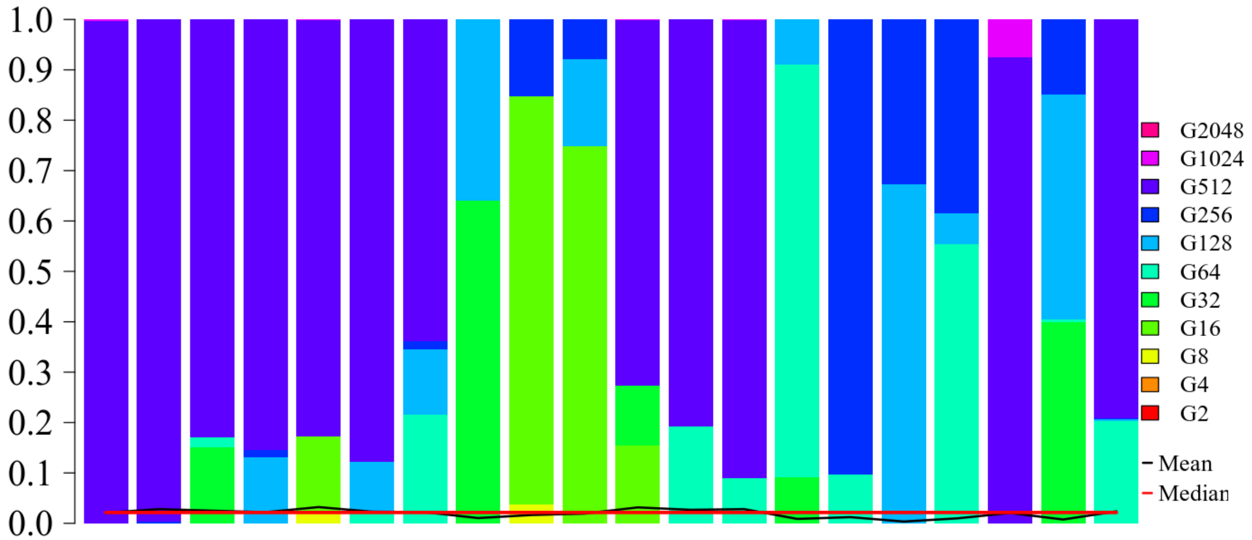

Supplementary Figure 2-26: Global inbreeding levels for each animal for Sonjo

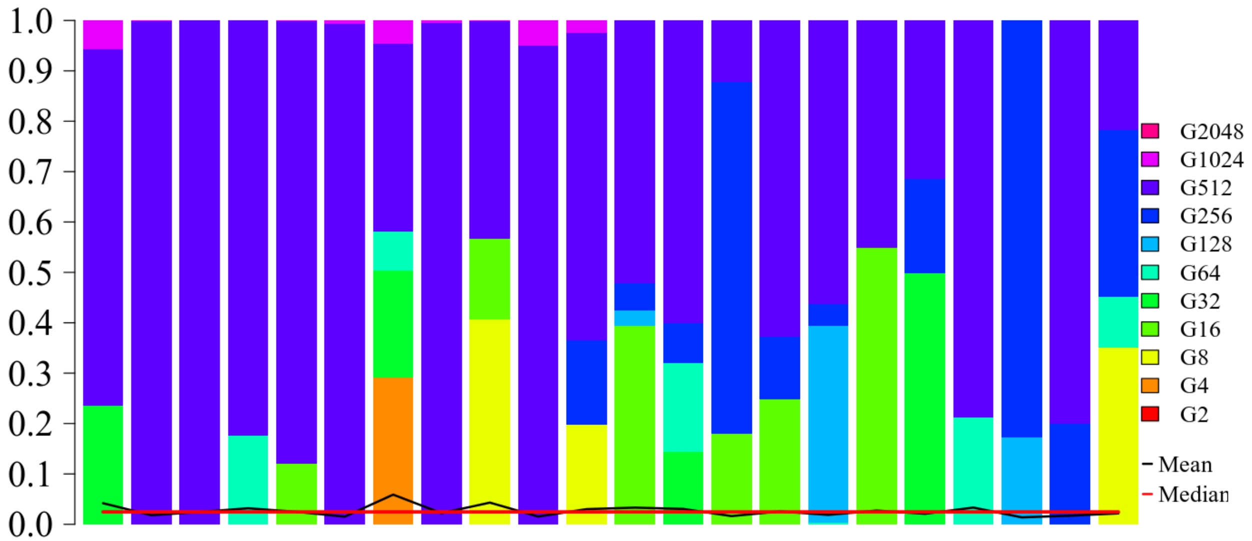

Supplementary Figure 2-22: Global inbreeding levels for each animal for Soudanaise

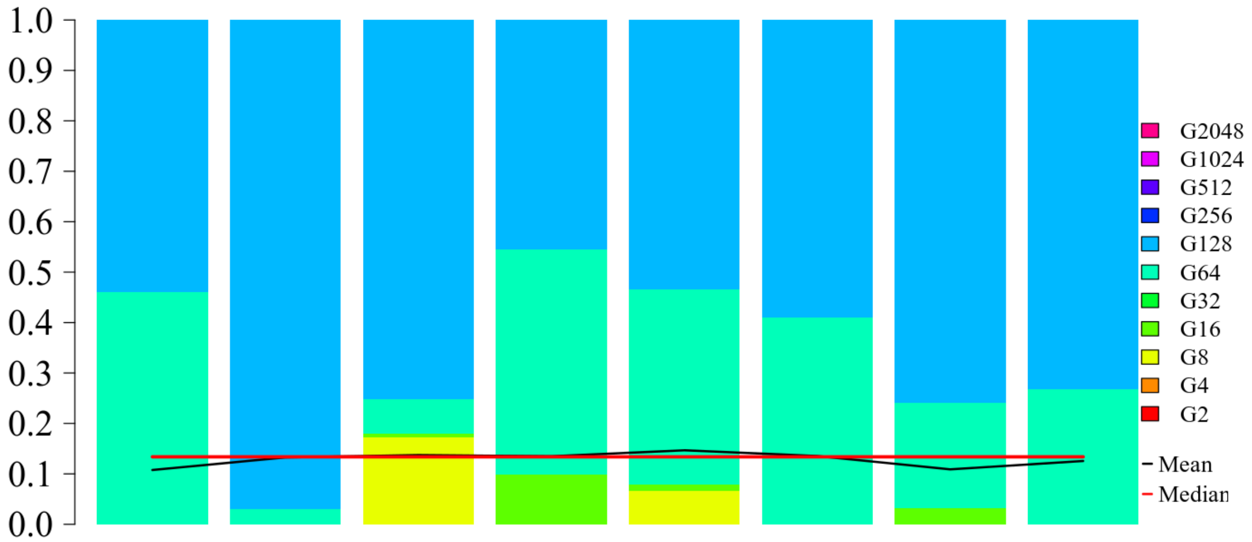

Supplementary Figure 2-28: Global inbreeding levels for each animal for SudOuest

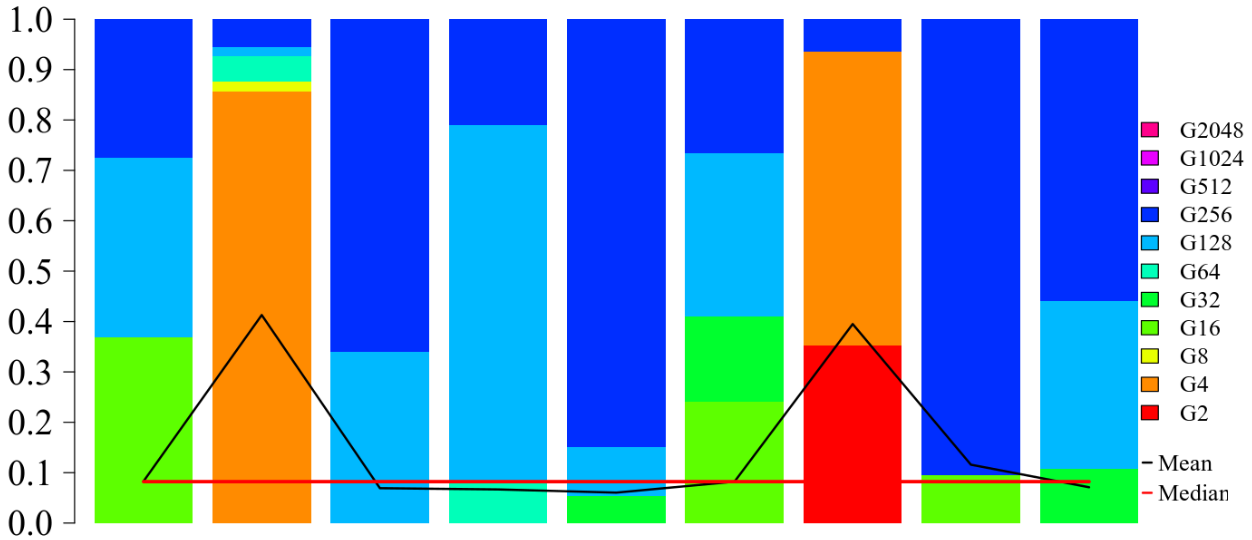

Supplementary Figure 2-29: Global inbreeding levels for each animal for Thyolo

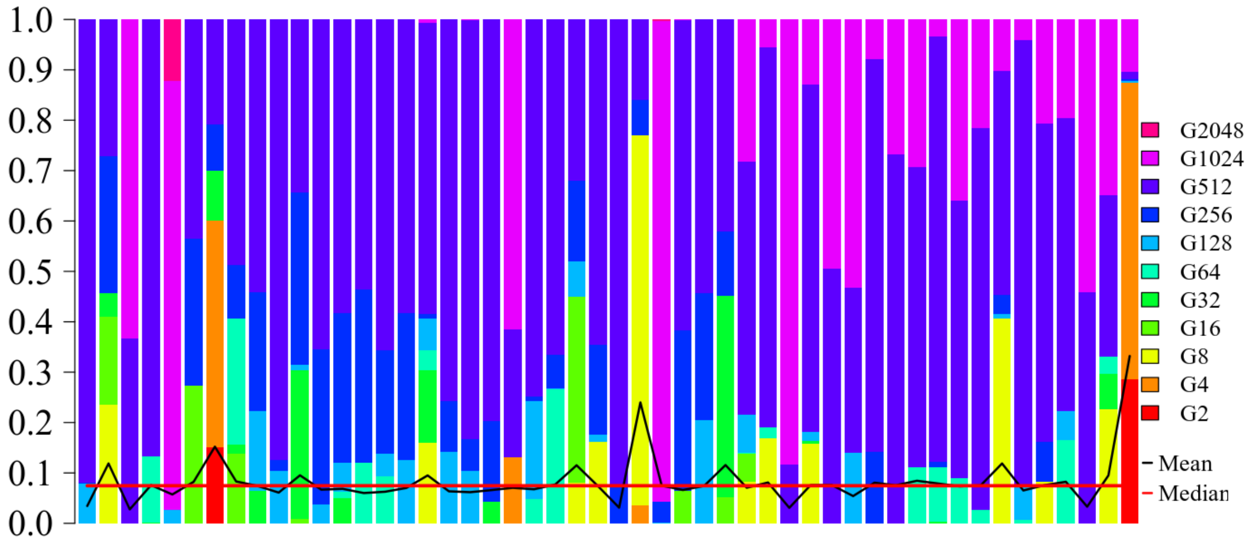

Supplementary Figure 2-30: Global inbreeding levels for each animal for West African Dwarf

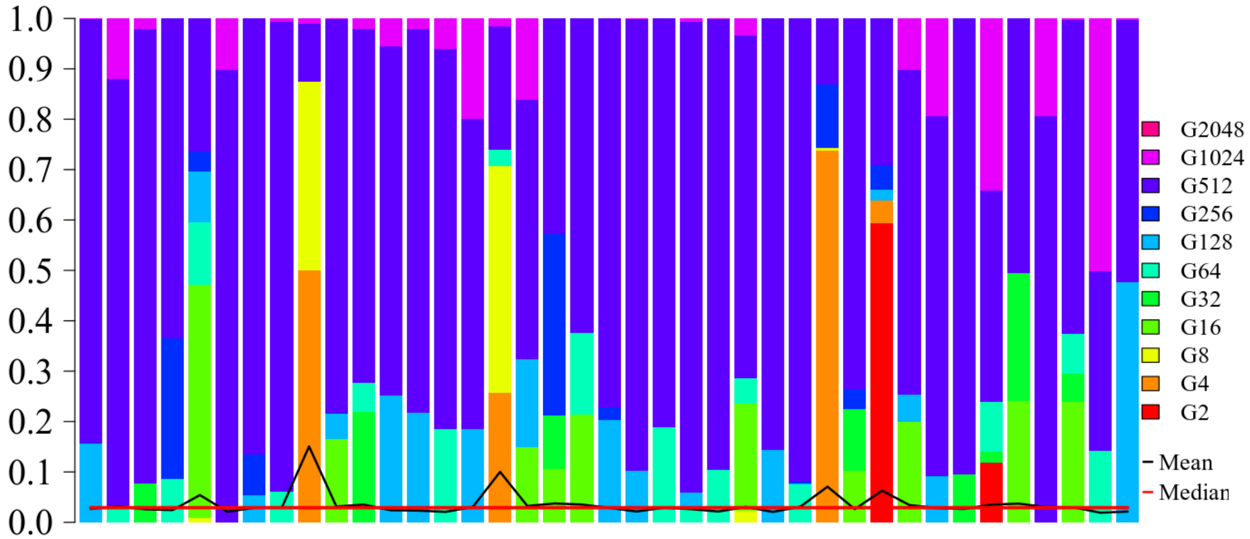

Supplementary Figure 2-31: Global inbreeding levels for each animal for Woyito Guji
